# Extreme pathway analysis reveals the organizing rules of metabolic regulation

**DOI:** 10.1371/journal.pone.0210539

**Published:** 2019-02-05

**Authors:** Yanping Xi, Fei Wang

**Affiliations:** 1 Shanghai Key Lab of Intelligent Information Processing, Fudan University, Shanghai, China; 2 School of Computer Science and Technology, Fudan University, Shanghai, China; 3 Shanghai Ji Ai Genetics & IVF Institute, Obstetrics and Gynecology Hospital of Fudan University, Shanghai, China; Christian Albrechts Universitat zu Kiel, GERMANY

## Abstract

Cellular systems shift metabolic states by adjusting gene expression and enzyme activities to adapt to physiological and environmental changes. Biochemical and genetic studies are identifying how metabolic regulation affects the selection of metabolic phenotypes. However, how metabolism influences its regulatory architecture still remains unexplored. We present a new method of extreme pathway analysis (the minimal set of conically independent metabolic pathways) to deduce regulatory structures from pure pathway information. Applying our method to metabolic networks of human red blood cells and *Escherichia coli*, we shed light on how metabolic regulation are organized by showing which reactions within metabolic networks are more prone to transcriptional or allosteric regulation. Applied to a human genome-scale metabolic system, our method detects disease-associated reactions. Thus, our study deepens the understanding of the organizing principle of cellular metabolic regulation and may contribute to metabolic engineering, synthetic biology, and disease treatment.

## Introduction

Organisms are constantly faced with variable internal physiological states and environmental conditions. The ability to rapidly shift between phenotypes to deal with these challenges is essential for the competitive fitness and survival of an organism [1–3]. It is well known that cellular response to internal and environmental perturbations is often reflected and/or mediated through changes in metabolism [3]. Such metabolic changes are often accomplished through both genetic and post-transcriptional controls, such as transcriptional regulation of gene expression and allosteric regulation of enzymes [4, 5]. Understanding the interaction between regulatory and metabolic processes is therefore a fundamental problem in biology.

The mechanisms controlling metabolic processes, including regulatory circuits and logics, have been intensively studied [6], especially since the emergence of massive ‘omics’ datasets in the post-genomic era [4]. Incorporating regulatory rules into constraint-based metabolic models allows researchers to more accurately predict the metabolic phenotype under different environmental and genetic perturbations [7–16]. However, these methods seldom provide insight into the regulatory architecture, i.e. the key control reactions, of the metabolic networks. Understanding how the regulatory architecture is designed, how it evolves, and what role the structure of the metabolic network plays in regulatory processes is an ongoing research challenge [17–20].

Previously, some studies [4, 21–24] derived regulatory patterns of a metabolic pathway with limited scale and complexity by optimizing an objective function that incorporates multiple criteria of metabolic processes, such as benefit and cost, under given conditions. Some investegated the contribution of metabolic network connectivity in metabolic control or regulation [17, 19, 20, 25, 26]. Some integrated gene-expression data and topological information of the genome-scale metabolic network to uncover transcriptional regulation under certain perturbations [3]. The research above, although still in its infancy, has reported promising findings that the organizing principles of metabolic control may be deduced from the connection structure of a metabolic system.

Metabolic pathway analysis, such as elementary mode analysis and extreme pathway analysis, has provided a new outlet to understand topological structures of metabolic networks and pathway regulations [27, 28]. Elementary modes [29] consists of the minimum number of reactions that exist as a functional unit [30]. By introducing the term ‘control-effective fluxes’, elementary mode analysis succeeded in predicting the gene expression ratios of central carbon metabolism in the growth of *Escherichia coli* and *Saccharomyces cerevisiae* on two alternative substrates [31–33], estimating the significance of links between metabolic processes and levan biosythesis in *Halomonas smyrnensis* [34], and describing the behavior of folate-related processes in human placenta [35]. As another widely used and highly relative concept of network-based pathways, extreme pathways form the unique set of systemically independent and non-decomposable steady-state flux distributions based on the system’s stoichiometry and thermodynamic constraints of a given metabolic network [36]. Extreme pathway analysis has already been used to hunt for regulation of metabolic systems by the approaches such as grouping and interpretation [37], Singular value decomposition (SVD) [38, 39], reaction participation analysis [40], feasible extreme pathway analysis [41] and alpha-specturm calculation [42, 43]. The approach of grouping and interpretation divides extreme pathways into groups based on some pre-set criteria and iterprete the metabolic and regulatory function of pathways in each group [37]. SVD produces eigenpathways by decomposing the extreme pathway matrix and shows that the eigenpathways correspond to the key control points in the network [38, 39]. Reaction participation analysis considers correlated reactions and the reactions that participate in a large number of extreme pathways as good targets for regulation [40]. Feasible extreme pathway analysis eliminates the extreme pathways that are inconsistent with regulatory constraints or physico-chemical constraints and shows that regulation forces a particular set of phenotypic behaviors to be expressed [41]. The alpha-spectrum defines the allowable range of extreme pathway contributions to a given steady state [42, 43].

However, the previous works also have their own shortcomings. Approaches of control-effective fluxes, feasible extreme pathways and alpha-spectrum are condition dependent. Namely, although they reveal important regulatory reactions in the given condition, they neglect regulatory reactions which function in other conditions. Grouping and interpretation is not available when the number of extreme pathway is large. Reaction participation analysis prefers the reactions with higher participation frequency, therefore it will miss out the reactions which participate less frequently in extreme pathways but still regulatory important. The approach of SVD is not intuitive enough since eigen pathways may not necessarily be biochemically feasible.

Above all, the role of regulatory reactions can be interpreted as reducing the uncertainty of the metabolic system from the perspective of information theory, because regulatory reactions put further constraints on metabolic system, reduce the space of steady sates and lead the metabolic system to a objective state [41]. Therefore, in order to hunt for the potential regulators, it is crucial to measure the role on average a reaction plays in eliminating the uncertainty of the metabolic system. To the best of our knowledge, no approach of metabolic pathway analysis had attempted such a measurement. Here, we developed a new method to address the issue.

Since any steady-state flux vector describing a metabolic phenotype of the cell can be thought of as a non-negative combination of these extreme pathways [44], it is logical to assume that a cell ‘switches’ on and off its extreme pathways by metabolic control to reach a particular metabolic steady-state [36]. In other words, although the regulation of particular reactions is ongoing within a cell, the cell’s ultimate aim may be to regulate the set of extreme pathways [36]. When the states of the extreme pathways are set, the information, what the target metabolic state should be, is passed from regulatory control to metabolism. Under this hypothesis, we evaluate the regulatory importance of a metabolic reaction by the role it plays in determining the “on/off” states of extreme pathways. This term takes into account both efficiency and flexibility, which are believed to be two major objectives of the evolutionary optimization process of metabolic regulation. Efficiency is the ability to fulfill the cellular demands of metabolic regulation at a minimal cost. For simplicity, supposing that equal investment is made to control each reaction in a given cell, then the cost of metabolic regulation can be estimated by the number of regulated reactions. Flexibility refers to the ability to maintain a quick and robust response against internal and enviromental perturbations. Generally, flexibility increases when more reactions are utilized for regulation. These two criteria impose challenges that are obviously contradictory; therefore, optimal regulatory architecture for metabolism needs to balance a trade-off between efficiency and flexibility.

In this study, we introduce a new method of extreme pathway analysis to deduce regulatory reactions of metabolic networks. Our method has the following advantages: 1) it is condition independent since all the extreme pathways consistent with mass balance constraints and reaction reversibility constraints are included in the analysis. 2) it can be applied on arbitrarily large number of extreme pathways as long as they can be enumerated. 3) it has no obvious preference for reaction participation. 4) rather than interpreting the regulatory effects by eigenpathways, we treated every metabolic reaction as a candidate regulator, which makes our method more intuitive. Our goal is to shed light on the universal organizing rules of regulated reactions in metabolic networks from the perspective of information theory and evolution.

We applied our method to the metabolic networks of the human red blood cell (hRBC) and *Escherichia coli* to study the architecture of allosterical and transcriptional regulation, respectively. The reactions of high regulatory importance show good agreement with findings from previous research. The results demonstrate a significant correlation between the topology of a metabolic network and its regulatory architecture. With the assumption that regulated reactions are likely associated with disease processes, we applied our method to a genome-scale human metabolic network to predict disease-associated reactions. Our computational results agree reasonably well with experimental results. Overall, extreme pathway analysis allows us to systematically investigate the organizing principle of metabolic regulation. Our findings suggest that the regulatory architecture of metabolism has evolved to put extreme pathways under more efficient and flexible controls. Our method may also be helpful in metabolic engineering and drug target discovering by recommending the reactions that are worthier of control.

## Methods

### Assigning exchange fluxes to a target subsystem

A *target subsystem* consists of a subset of internal reactions in the whole metabolic network. The complement of a target subsystem constitutes another subsystem called the *surrounding subsystem*. The metabolite set of a subsystem contains all the metabolites that appear as substrates or products of at least one reaction in it.

The process to assign exchange fluxes starts by identifying the shared metabolites between the target and surrounding subsystems. For each shared metabolite M, a duplicate metabolite $M^{\prime }$ is introduced to substitute M in reactions of the surrounding subsystem. Meanwhile, a bidirectional reaction $M↔M^{\prime }$ is added to the original metabolic network and an exchange flux associated with M is introduced into the target subsystem. Obviously, the reaction $M↔M^{\prime }$ describes the exchange of metabolite M between the target and surrounding subsystems. Thus, the flux of reaction $M↔M^{\prime }$ should be equal to the exchange flux associated with M in the target subsystem. Next, the maximum and minimal fluxes of $M↔M^{\prime }$ are determined through flux variability analysis [45] by setting the flux of reaction $M↔M^{\prime }$ as the objective function. These values indicate the possible production and/or consumption of M in the target subsystem. Table 1 covers all 4 possible input and output combinations between the maximum and minimal values. Based on this information, flux constraints can be specified for each exchange reaction in the target subsystem. With all the exchange fluxes being properly added and constrained, the target subsystem is ready for extreme pathway computation.

**Table 1:** Logic table for determining the constraints of the exchange reaction that transports metabolite M in and out of the target subsystem based on flux variability analysis of the reaction $M↔M^{\prime }$. The forward direction of $M↔M^{\prime }$ is defined as from M to $M^{\prime }$. And the forward direction of the exchange reaction is defined as taking M away from the target subsystem. Min, minimum; Max, maximum.

Case	Exchange flux of a subnetwork		Available direction (Flux constraint)
	Min.	Max.	
1	< 0	> 0	Out and in (unconstrained)
2	= 0	> 0	Out (positive)
3	< 0	= 0	In (negative)
4	= 0	= 0	None (zero)

Although adding a reaction $M↔M^{\prime }$ is equivalent to specifying a compartment for each target subsystem, unlike the previous study [46], the procedure will not change the predicted behaviors of the whole metabolic network. Because the added reaction has only one substrate and one product, it is proven in Statement G in S1 Text that adding such a reaction does not change the constraint of the fluxes of steady states.

The process described above is illustrated by Fig A in S1 Text. This protocol works effectively for any metabolic network and any delimitation of the target subsystems.

### Calculating extreme pathways

The extreme pathways of the hRBC metabolic network were derived straightforwardly from a published open-source bioinformatic software program, EXPA [47]. As for the target subsystems of *E. coli* and human metabolism, we applied the above protocol for the exchange flux assignment and then computed the extreme pathways with EXPA [47]. The extreme pathways of type I and II that comply with Kirchhoff’s second law [48] were passed to the following analysis. Each pair of elements corresponding to a reversible internal reaction in an extreme pathway was merged together by subtracting the reverse flux from the forward one. The resulting vector is termed *compact extreme pathway*. Elements in a compact extreme pathway have a one-to-one correspondence to reactions in the metabolic network. It is proven in Statement D in S1 Text that converting from an extreme pathway to its compact form does not change the set of reactions it utilized.

### Sorting internal EqSets by their regulatory importance

A greedy algorithm was built to sort internal EqSets of internal reactions by their regulatory importance. Briefly, it begins with a pool P of all the EqSets and an empty queue of predicted regulatory EqSets, Q. Then, it iteratively picks up the EqSet with the highest regulatory importance (defined in the following sections) from P and moves it to the end of Q. The iteration stops when P is empty or the regulatory importance of any EqSet remaining in P equals zero. At last, the algorithm removes the EqSets of exchange reactions from Q and outputs Q as the resulting sequence. More details about the algorithm are provided in Algorithm A in S1 Text.

### Shannon entropy and conditional entropy of reactions

Shannon entropy and conditional entropy are used to measure the role on average of a reaction in eliminating the uncertainty of a metabolic system. Suppose a metabolic network of *n* reactions, $X=R_{1},R_{2},\cdots ,R_{n}$, has *l* compact extreme pathways, [**e**^1^, **e**^2^, ⋯, **e**^*l*^]. Then, a reaction $R_{i}$ is employed by a compact extreme pathway **e**^*j*^ if and only if $e_{i}^{j}\neq 0$, where $e_{i}^{j}$ is the *i*th element of **e**^*j*^, 1 ≤ *i* ≤ *n* and 1 ≤ *j* ≤ *l*. We define **e**^*j*^ as an *employer extreme pathway* of $R_{i}$. Further, we consider $R_{i}$ to be ‘on’ if any of its employer extreme pathway is utilized by the metabolic system to form the current steady-state, and ‘off’ otherwise. Assume that each extreme pathway is utilized with equal opportunity. The probability of the on/off state of $R_{i}$ is defined as follows: $P(R_{i}\;is\;\text{on})$ equals the fraction of extreme pathways in which $R_{i}$ is employed and $P(R_{i}\;is\;\text{off})$ equals $1-P(R_{i}\;is\;\text{on})$. The joint probability distribution can be similarly derived by counting the number of compact extreme pathways that consist of certain reactions.

Based on the above probability distributions, the Shannon entropy $H(R_{i})$, combination entropy $H(X_{1})$ as well as conditional entropy $H(X_{1}|X_{2})$ can be defined according to the information theory [49], where $X_{1}$ and $X_{2}$ are two subsets of X. In particular, if reactions $R_{i}$ and $R_{j}$ satisfy that $H\left(R_{i}|R_{j}\right)=H\left(R_{j}|R_{i}\right)=0$, they form an *equivalent reaction couple on Shannon entropy*.

### Regulatory distance between reactions/EqSets

A weighted graph *G* was built for each metabolic system or subsystem in which individual reactions are nodes and the weight of the arc connecting two nodes, $R_{i}$ and $R_{j}$, equals the *local regulatory distance*, $d(R_{i},R_{j})$, between the two reactions.

$$\begin{array}{c} d\left(R_{i},R_{j}\right)=\{\begin{array}{cc} 0 & \text{if}\;i=j,\\ \infty & \text{if}\;M_{c}=\emptyset ,\\ \min_{M\;\in \;M_{c}}\left(C\left(M\right)\right) & \text{otherwise}, \end{array} \end{array}$$ (1)

where $M_{c}$ is the intersect of the metabolites of $R_{i}$ and $R_{j}$, and C(M) equals the number of reactions that use M as a substrate. Note that any metabolite involved in a reversible reaction is considered to be both a substrate and a product. Without considering the change of enzyme activity, the flux variation of a reaction will change the fluxes of the adjacent reactions by alter the concentration of shared metabolites. If the concentration of the shared metabolite can also be altered by other reactions, then the flux change of the adjacent reaction will be diminished. Therefore, the local regulatory distance between two reactions measures the direct impact on the flux of one reaction caused by a flux change of the other.

The *global regulatory distance*, $D(R_{i},R_{j})$, between $R_{i}$ and $R_{j}$ equals the length of the shortest route between the corresponding nodes on the weighted graph, *G*. Intuitively, the global regulatory distance measures the indirect impact of flux change between two reactions.

The regulatory distance of two EqSets $D(X_{1},X_{2})$ is defined as the average of $D(R_{i},R_{j})$, where $X_{1}$ and $X_{2}$ are two EqSets, $R_{i}\in X_{1}$, and $R_{j}\in X_{2}$.

### Equivalent reaction set on Shannon entropy

First, each exchange reaction forms an individual *equivalent reaction set on Shannon entropy* (EqSet). Second, an EqSet of *k* (*k* ≥ 1) internal reactions $X=\{R_{1},R_{2},\cdots ,R_{k}\}$ has the properties that

1. ∀*i*, *j* = 1, ⋯, *k*, $R_{i}$ and $R_{j}$ forms an equivalent reaction couple on Shannon entropy, i.e. $H\left(R_{i}|R_{j}\right)=H\left(R_{j}|R_{i}\right)=0$;
2. ∀*i* = 1, ⋯, *k*, $\min_{j=1,\cdots ,k,j\neq i}D\left(R_{i},R_{j}\right)\leq \rho_{e}$, where *ρ*_*e*_ is the effective radius within an EqSet that represents the maximum regulatory distance of any reaction to its nearest counterpart in the same EqSet.

### Measuring the regulatory importance of an EqSet

Given a metabolic system with *k* EqSets, $P=\{X_{1},X_{2},\cdots ,X_{k}\}$, and a queue of EqSets $Q=[X_{1},X_{2},\cdots ,X_{i}]$ (0 ≤ *i* < *k*) that are predicted to be regulatory, the regulatory importance of an EqSets is determined by the nearby EqSets within the distance of *ρ*_*s*_ because of the constraint that the regulatory influence of an EqSet $X_{j}$ (*i* < *j* ≤ *k*) exists locally, where *ρ*_*s*_ is the effective radius between EqSets that designates the maximum regulatory distance that an EqSet influences. We built two group of EqSets with the nearby EqSets of $X_{j}$: First, the regulatory neighbor set $N_{j}$ consists of at most *τ* nearby EqSets standing at last of the queue Q, where *τ* is the size of a window sliding on Q. And second, the competitor set $C_{j}$ consists of the nearby EqSets that are not in Q.

The regulatory importance, $val(X_{j})$ (*i* < *j* ≤ *k*), of $X_{j}$ is measured from two aspects: one is the average information it provides to the metabolic system conditioned on the regulatory neighbor set and the other is its non-substitutability among the competitor set. Formally, the former equals $H(X_{j}|{\hat{N}}_{j})$, and the latter equals $\min_{X_{u}\in C_{j}}\left(H\left(X_{j}|{\hat{N}}_{j}\cup X_{u}\right)\right)$, where ${\hat{N}}_{j}$ is the set of reactions participating in any regulatory neighbor EqSets of $X_{j}$, i.e., ${\hat{N}}_{j}=\underset{X\in N_{j}}{⋃}X$. Lastly the summation of the two aspects is adjusted by the weight $1+\mu (|X_{j}|-1)$, which gives more bonus to larger EqSets. The amount of bonus for an extra reaction in $X_{j}$ is modulated by bonus ratio, *μ*. To sum up, $val(X_{j})$ is represented as

$$\begin{array}{c} val\left(X_{j}\right)=\left[1+\mu (\right|X_{j}\left|-1)\right]\left[H\left(X_{j}|{\hat{N}}_{j}\right)+\min_{X_{u}\in C_{j}}H\left(X_{j}|{\hat{N}}_{j}\cup X_{u}\right)\right]. \end{array}$$ (2)

### EqSet sequence evaluation and the corresponding *p*-value

The output EqSet sequence Q ($Q=[X_{1},X_{2},\cdots ,X_{s}]$) of the above algorithm is evaluated by summing up the ranks of the EqSets that contain regulatory or disease-related (for the human metabolic network) reactions, where *s* is the length of Q. If this sequence covers only a part of the internal EqSets, a penalty is added to the rank summation, representing the missing EqSets that contain regulatory or disease-related reactions. The final evaluation score *σ* is represented as

$$\sigma =\sum_{\begin{array}{c} i=1,\cdots ,s\\ X_{i}\cap {\mathbb{R}}^{bm}\neq \emptyset \end{array}}rank(X_{i})+d(s+\frac{l+1}{2}),$$ (3)

where $R^{bm}$ is the set of biologically meaningful reactions, i.e., the regulatory or disease-related reactions, $rank(X_{i})$ is the rank of $X_{i}$ in Q, *l* is the number of lost internal EqSets and *d* is the number of the missing internal EqSets that contain biologically meaningful reactions. It is proven in Statement H in S1 Text that the penalty part $d(s+\frac{l+1}{2})$ equals the expectation of the rank summation of the lost EqSets that contain biologically meaningful reactions when all the missing EqSets are placed at the tail of the output sequence in a random order.

For an output sequence of the sorting algorithm whose evaluation score is *σ*_0_, the corresponding *p*-value is defined as the probability that a randomly arranged sequence of internal EqSets has an evaluation score no higher than *σ*_0_. Although the *p*-value can be estimated by stochastic simulation, we provide a dynamic algorithm in Algorithm B in S1 Text to meet the demands for precise computation.

### Heuristically searching for the optimal setting of the parameters

A heuristic search algorithm was built in order to quickly find the nearly optimal values for the four parameters (i.e., *μ*, *ρ*_*e*_, *ρ*_*s*_ and *τ*) used in the EqSet sorting algorithm. Assume that the ranges of *μ*, *ρ*_*e*_, *ρ*_*s*_ and *τ* are limited and discrete, the algorithm starts from a random point $\left(\mu^{0},\rho_{e}^{0},\rho_{s}^{0},\tau^{0}\right)$, which are randomly selected from their respective ranges. While keeping three of the parameters at fixed values, the search strategy sequentially updates one parameter at a time to its optimum value, which results in a sorted EqSet sequence with the lowest *p*-value. When all four parameters have been adjusted, the search algorithm moves on to the next iteration by updating from the first one again. If a local optimal setting of the parameters is found, the algorithm will start another try to search for the local optimal parameters from another randomly picked starting point. The algorithm will stop and return the best parameter values among all the tries when it reaches the maximum number of search steps. The search algorithm described above is illustrated in Fig D in S1 Text.

### Evaluating the capability of the sorting algorithm for disease-related EqSet prediction

Since the EqSets of a certain metabolic network vary with the parameter *ρ*_*e*_, we first divided the range of *ρ*_*e*_ into several separate subsets so that values in a same subset will result in exactly the same EqSets. In each subset, we randomly selected *x*% of the internal EqSets as a training set and left the other (100 − *x*)% as a test set, where *x* ∈ {10, 20, 30, 40, 50, 60, 70, 80, 90}. Given a sequence of internal EqSets, we extracted the subsequence that consists of the EqSets in the training set, on which the *p*-value for the training set is computed. We found the optimal setting of the parameters and the corresponding *p*-value for the training set. We repeated this process 100 times, which provided the mean of the *p*-values on the training sets. After that, we selected the subset of *ρ*_*e*_ with the lowest average *p*-values and counted the average rate of the disease-related EqSets being in the top 10% of the test set sequences. This rate shows the capability of the sorting algorithm for predicting disease-related EqSets.

## Results

Reactions that play equal roles in determining the states of extreme pathways are merged as an equivalent reaction set of Shannon entropy (EqSet), in which each reaction pair satisfies the assumption that the conditional entropy of one reaction, given the other, equals zero. An EqSet is treated as a potential entity for metabolic regulation. The EqSet that contains at least one regulated reaction is defined as a regulatory EqSet. A greedy algorithm is used to organize the EqSets in a sequence according to their regulatory importance. The algorithm starts from an empty sequence. Under the assumption that the EqSets in the current sequence are regulatory, the algorithm iteratively picks up the EqSet from the rest that is most important for regulation and adds it to the end of the sequence. The EqSets ranked higher in the sequence are expected to have greater likelihood of containing strictly regulated reactions. The resulting EqSet sequence is evaluated by a *p*-value, which is the probability that the rank summation of the regulatory EqSets on a random sequence is lower than that on the sequence given by our algorithm. (See Methods and Statement I in S1 Text).

The regulatory importance of an EqSet is defined by regulatory efficiency and flexibility. The regulatory efficiency of an EqSet is measured by the conditional entropies that depend on the reactions’ participation ratios on the extreme pathways. It characterizes the EqSet’s average effect on determining the “on/off” state of each extreme pathway, as well as the degree to which its regulatory function cannot be replaced by other EqSets. The flexibility of an EqSet is modeled in two aspects: First, the regulatory distances between reactions and between EqSets are defined according to the topology of the metabolic network. And the regulatory influence of a reaction is restricted to a local scope in order to speed up the shift of a cell’s metabolic states. Therefore, an EqSet is restriced further to include only reactions within certain regulatory distance given by a parameter, intra-EqSet effective radius (denoted as *ρ*_*e*_). And the regulatory influence of an EqSet is restricted to other EqSets within the regulatory distance given by a parameter, inter-EqSet effective radius (denoted as *ρ*_*s*_). Second, a sliding window of size *τ* is employed on the sequence to define the available regulatory EqSets. The Eqset that leaves the window is considered to be out of control, which simulates a disturbance in the regulatory system. Thus, the EqSet that best compensates for the disturbance will be considered to be of high flexibility in this case. Briefly, the values of *ρ*_*e*_, *ρ*_*s*_ and *τ* have an inverse relation with the degree of flexibility, and consequently reflect a trade-off between efficiency and flexibility.

The regulatory importance of an EqSet calculated above is then adjusted according to the size of the EqSet for the reasons listed below: Assuming that each metabolic reaction has an equal probability of obtaining a regulatory function because of gene mutations, it is more likely that a mutation that brings a regulatory function to a reaction occurred earlier in a bigger EqSet than in a smaller one. If the reaction happened to be of regulatory importance, the mutation is more likely to increase in frequency within the population by means of natural selection. Thus, an EqSet that contains more members will gain some advantages in acquiring regulated reactions compared to smaller counterparts with reactions that have similar degrees of regulatory importance. Therefore, we give a bonus for each extra reactions in an EqSet. The amount of the bonus is controlled by a parameter, bonus ratio (denoted as *μ*).

We use the metabolic reconstructions of the human red blood cell (hRBC) [37], *E. coli* (iJR904) [50] and global human cells (H. sapiens Recon 1) [51] as examples to explore the utility of our method. These models were obtained from http://systemsbiology.ucsd.edu. As network properties and regulation demands differ between metabolic systems, the parameters *ρ*_*e*_, *ρ*_*s*_, *τ* and *μ* have to be optimized case by case.

### Identifying allosterically regulated reactions in hRBC metabolism

The human erythrocyte is a complete cell with a simple metabolic system. It has been well studied, which makes it an attractive case for our research. The hRBC metabolic model used here consists of 39 metabolites and 51 reactions, of which 19 are exchange reactions (See Tables A and B in S1 Text). It contains four classical pathways: glycolysis, the pentose pathway, adenosine nucleotide metabolism, and the Rapoport-Luebering shunt [37]. The computation of the extreme pathways of this model resulted in 36 type I, 3 type II, and 16 type III extreme pathways. (See Figs F, G and H in S1 Text for type I and II extreme pathways.) Type III extreme pathways are reported to be thermodynamically infeasible [48], so they are neglected in the remaining analysis. Metabolism in the hRBC is mainly regulated by allosteric enzymes that control the cell’s production of the cofactors necessary to maintain osmotic balance and electroneutrality, and to fight oxidative stresses [37, 52, 53]. We obtained 10 regulated reactions from the literature [54–63]. Lists of the reactions and regulatory mechanisms are detailed in Table C in S1 Text.

The relatively simple structure of the hRBC metabolic network ensures that the influence of any regulated reaction may quickly spread throughout the network. In addition, the human body employs huge numbers of erythrocytes to fulfill the task of transporting and exchanging oxygen and carbon dioxide; thus, cells with appropriately functioning regulatory processes could likely compensate for regulatory deficiencies in other cells. Both factors, a simple network structure and redundancy through a large quantity, may reduce the evolutionary demands on flexibility for the hRBC regulatory architecture. Therefore, we set parameters *ρ*_*e*_, *ρ*_*s*_, and *τ* to infinity so that the regulatory importance measurement focuses on the regulatory efficiency of each reaction.

**Fig 1:**
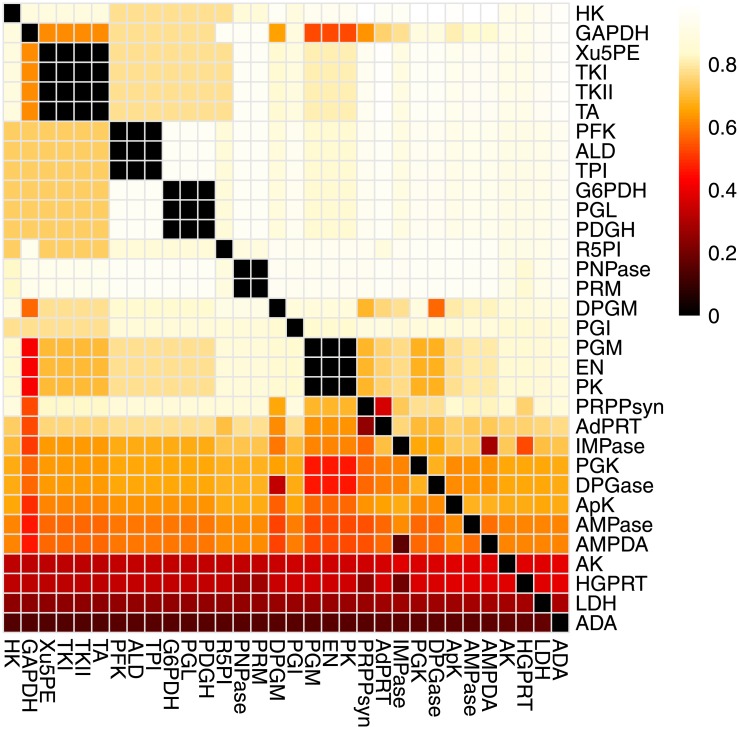
Heat map showing conditional entropy of internal reaction pairs of the hRBC metabolic network. The heat map colors represent the conditional entropy of each reaction at the beginning of each line, given the reaction listed at the bottom of each column. The black blocks on the diagonal represent the internal EqSets.

For each pair of internal reactions, we calculated the conditional entropy of one participant’s distribution given the other’s and vice versa (Fig 1). The black blocks on the diagonal show 22 internal EqSets, of which 5 have more than one member reaction. There are 8 regulatory EqSets in the hRBC metabolic network. The entropies of the regulatory EqSets are higher than those of the non-regulatory EqSets (Wilcoxon rank sum test, *p*-value = 0.0121, Table D in S1 Text).

**Fig 2:**
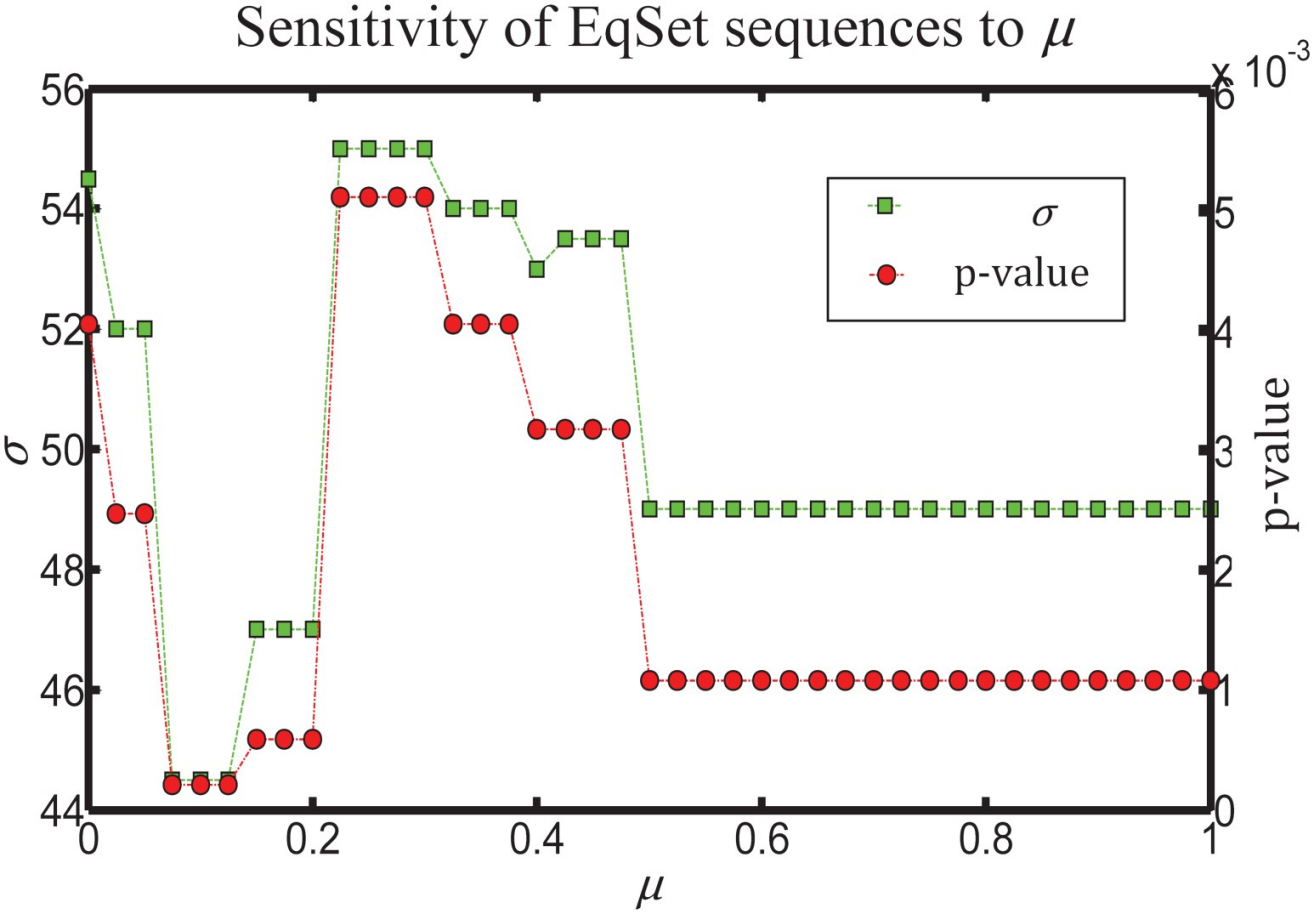
*P*-value (the line with circles) and evaluation score *σ* (the line with squares) of the resulting EqSet sequence of hRBC metabolic network as a function of the parameter *μ*. As *μ* ranges from 0 to 1 with an increasing step of 0.025, *p*-value varies between 2.10 × 10^−4^ and 5.1 × 10^−3^, and *σ* varies between 44.5 and 55.

**Fig 3:**
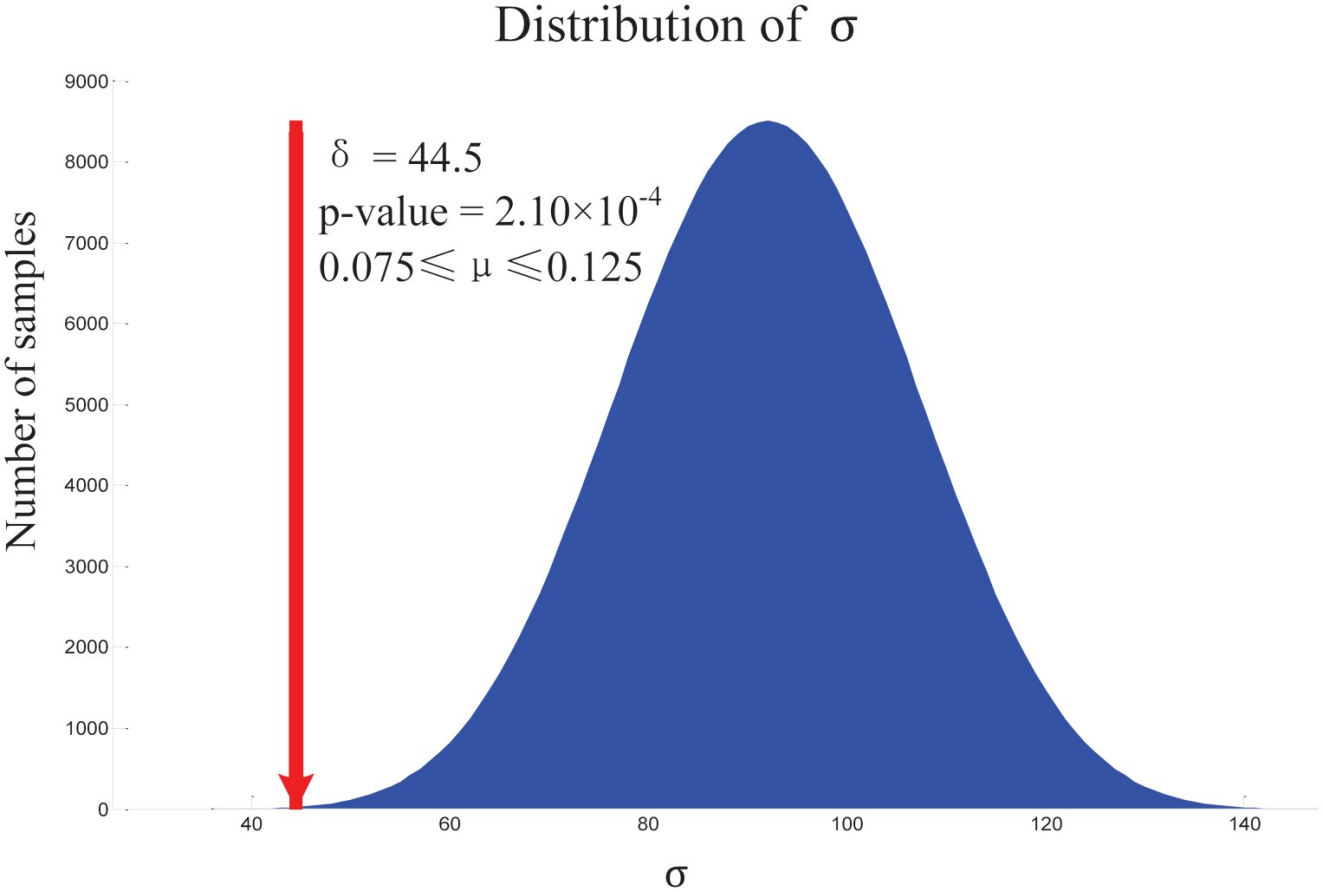
Evaluation score distribution of randomly organized EqSet sequences of hRBC metabolic network. The arrow denotes the EqSet sequence shown in Table 2.

By plotting *p*-values of resulting sequences against the values taken by the parameter *μ*, we found that *μ* plays a minor role in the output sequence in general (Fig 2). However, the *p*-value decreases when *μ* increases from 0 to 0.125, and then turns back when the bonuses are overestimated, i.e., when *μ* continuously increases to 1. The relatively low *p*-values indicate that our estimation of the regulatory importance of the EqSets agrees well with estimations from previous research. The lowest *p*-value, i.e. the lowest rank summation *σ*, is achieved when *μ* is set between 0.075 and 0.125 (*σ* = 44.5, *p*-value = 2.10 × 10^−4^, Fig 3). The corresponding sequence contains 8 internal EqSets (Table 2), seven of which are regulatory EqSets. The reactions in the other internal EqSets are considered to be less important for regulation. The only missing regulated reaction in the sequence is ‘HK’, catalyzed by hexokinase. It is known that the subtypes of hexokinase in hRBCs are HK-I and HK-R [64, 65], which are inhibited by Glucose-6-phosphate dehydrogenase (G6PDH) and ADP [54]. However, the inhibition could be eliminated by a minimal amount of inorganic phosphate [54]. Thus, the control response of ‘HK’ in hRBC metabolism is not significant, which is consistent with our results.

**Table 2:** The internal EqSet sequence of hRBC metabolic network in descending order of regulatory importance. The regulated reactions are denoted in boldface type. Full names for the abbreviations are listed in Table B in S1 Text.

EqSet	Rank
**TKI**, **TKII**, Xu5PE, TA	**1**
**DPGM**	**2**
**PRPPsyn**	**3**
**PFK**, ALD, TPI	**4**
**G6PDH**, **PDGH**, PGL	**5**
**PK**, PGM, EN	**6**
AMPase	7
**AdPRT**	**8**

**Fig 4:**
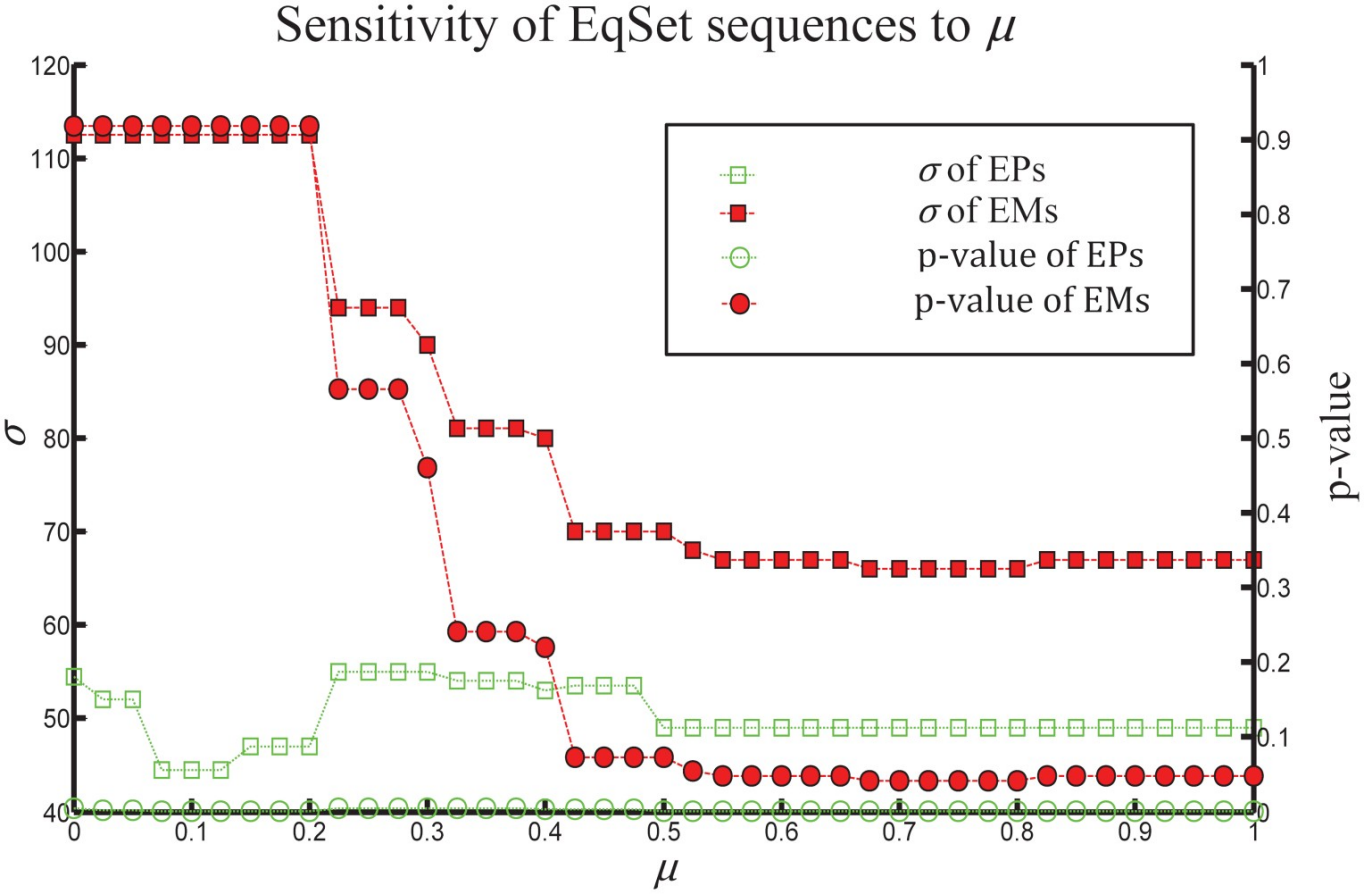
The resulting EqSet sequences calculated from extreme pathways (EPs; the lines with soft dots) versus those calculated from elementary modes (EMs; the lines with hard dots) in p-value (the lines with circles) and evaluation score *σ* (the lines with squares). As *μ* ranges from 0 to 1, *p*-value of the EqSet sequences calculated from EMs varies between 0.0408 and 0.9173, and the corresponding *σ* varies between 66 and 112.5. These values are always higher than those of the sequences calculated from EPs.

As a comparison, we applied our method to elementary modes (Fig 4). The result shows that our method performed worse on elementary modes than on extreme pathways, no matter the value given to the parameter *μ*. The difference in performance may be caused by the fact that extreme pathways are systemically independent (i.e., it is impossible to describe any extreme pathway as the sum of the others) whereas elementary modes are not.

**Fig 5:**
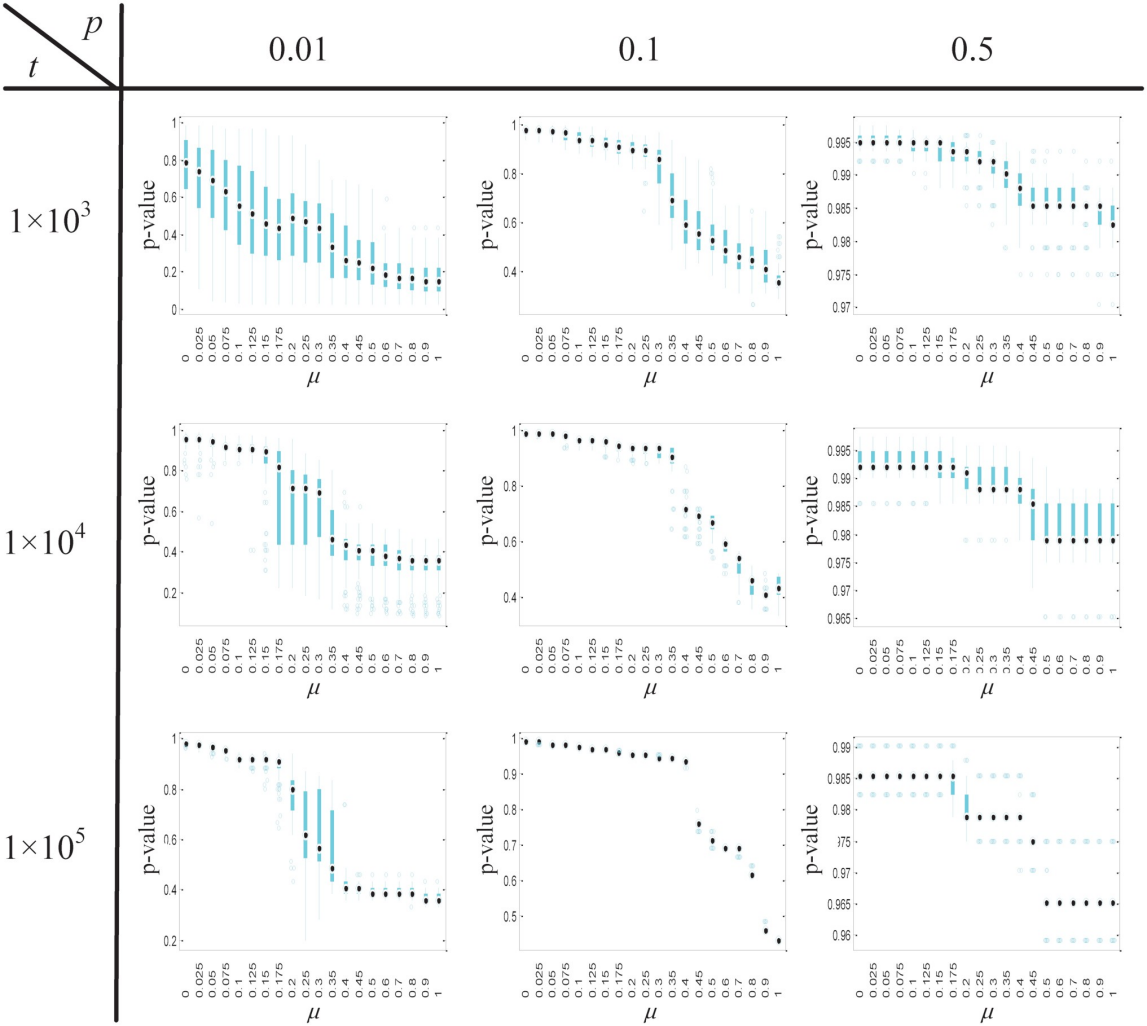
*P*-value distribution of the EqSet sequences calculated from artificial metabolic pathways of hRBC metabolic network. A set of artificial metabolic pathways are built as follows: (1) An artificial pathway is a summation of several randomly selected EPs. (2) The probability an EP being selected is *p*. (3) Altogether, *t* artificial pathways are generated, and the unique ones form the set. One hundred different artificial pathway sets are built for certain values of *p* and *t*. The distributions of the number of artificial pathways and the number of EPs contained in an artificial pathway in the sets generated at different values of *p* and *t* are shown in Fig I in S1 Text.

We further compared extreme pathways and artificial metabolic pathways, which are generated by adding up randomly selected extreme pathways (Fig 5 and Fig J in S1 Text). There are two parameters, *p* and *t*, that affect the number and complexity of the artificial pathways (see Methods and Algorithm C in S1 Text). Briefly, the higher the values of *p* and *t*, the greater are the number and complexity of the artificial pathways (Fig I in S1 Text). As *p* or *t* increase, the artificial pathways are less similar to the extreme pathways, and the *p*-values of the resulting EqSet sequences increase substantially.

In summary, the results imply that extreme pathways are likely to be the real targets of metabolic regulation.

### Predicting transcriptionally regulated reactions in the *E. coli* metabolic network

The model iJR904 of *E. coli* accounts for 904 genes, 761 metabolites and 1,075 reactions, including 931 internal reactions and 143 exchange reactions [50]. Each reaction has a subsystem label indicating its metabolic function. A regulatory model originally designed for iJR904, namely iMC1010v1 [9], makes iJR904 a suitable object of our study.

The metabolic network of *E. coli* is so complex that it is impossible to enumerate all the extreme pathways in a reasonable time period. Therefore, we defined three subnetworks that represent amino acid metabolism, hydrocarbon metabolism and lipid metabolism, respectively. The internal reactions of a subnetwork are composed of the reactions functionally related subsystems (Table 3). Some reactions participate in more than one subnetwork. Exchange reactions were assigned to each subnetwork and then the extreme pathways were computed (see Methods). Table 4 summarizes the number of internal, exchange reactions and extreme pathways in each subnetwork, as well as the proportion of regulated reactions. The regulated reactions are identified by combining the gene-protein-reaction association [50] and the gene’s logical transcriptional regulatory rules of model iMC1010v1 [9], which resulted in 474 transcriptional regulated reactions (See S1 Table).

**Table 3:** Subsystems contained in each metabolic sub network of *E.coli*.

Subnetwork	Subsystems
Amino acid metabolism	Alanine and aspartate metabolism; alternate carbon metabolism; arginine and proline metabolism; cofactor and prosthetic group biosynthesis; cysteine metabolism; folate metabolism; glutamate metabolism; glycine and serine metabolism; histidine metabolism; methionine metabolism; threonine and lysine metabolism; tyrosine tryptophan and phenylalanine metabolism; unassigned; valine leucine and isoleucine metabolism
Hydrocarbon metabolism	Citric acid cycle; cofactor and prosthetic group biosynthesis; folate metabolism; glycolysis gluconeogenesis; glyoxylate metabolism; methylglyoxal metabolism; oxidative phosphorylation; pentose phosphate pathway; putative; unassigned
Lipid metabolism	Anaplerotic reactions; cell envelope biosynthesis; citric acid cycle; cofactor and prosthetic group biosynthesis; folate metabolism; membrane lipid metabolism; methylglyoxal metabolism; nitrogen metabolism; oxidative phosphorylation; pyruvate metabolism

**Table 4:** The number of internal reactions, exchange reactions, extreme pathways and regulatory or disease-associated reactions in each subnetwork. The values in the parentheses are the proportion of regulatory or disease-associated reactions among the internal reactions. EP, extreme pathway; RXN, reaction; Reg-, regulatory; Dis, disease-associated.

Subnetwork	No. internal RXNs	No. exchange RXNs	No. EPs	No. Reg- or Dis- RXNs (Rate)
Amino acid metabolism (*E. coli*)	255	141	385	167 (65.49%)
Hydrocarbon metabolism (*E. coli*)	114	87	396	54 (47.37%)
Lipid metabolism (*E. coli*)	127	83	239	65 (51.18%)
Hydrocarbon metabolism (human)	333	320	922	101 (30.33%)
Amino acid metabolism (human)	365	288	402	107 (29.32%)

The regulatory architecture of the metabolic network of *E. coli* is more flexible than that of hRBC. There are two reasons for this difference: first, the metabolic network of *E. coli* is much more complex so that a regulated reaction may have a relatively limited scope of influence. Second, in order to maintain survival, *E. coli* needs extra regulated reactions to compensate for the potential deficiency in metabolic control. This requires that the parameters *ρ*_*e*_, *ρ*_*s*_ and *τ* take appropriate values that will properly characterize the evolutionary demands of flexibility.

According to the size of each subnetwork and the regulatory distance between pairs of reactions in the subnetwork(Table 4 and **Fig K in** S1 Text), we expand the possible ranges for *μ*, *ρ*_*e*_, *ρ*_*s*_ and *τ* to a relatively wide ranges given in Table 5. Under the constraint that *ρ*_*e*_ is no higher than *ρ*_*s*_, we built a candidate set that consists of all 210,600 different values of the parameters that were used to make the EqSet sequences. For each subnetwork, we found that regulatory EqSets ranked significantly higher (*p* < 0.05) on most sequences (Table 6), which suggests that the major factor affecting the architecture of metabolic regulation is the reaction participation ratio on extreme pathways rather than any of the parameters. Moreover, the target subnetworks show diverse preferences for the candidate parameter values. For example: 1) The *p*-value < 0.05 candidate parameter values for human hydrocarbon drops substantially when compared to the E.coli model performance. 2) Lower *p*-values are achieved at certain parameter values for each target subnetwork. Differences in network properties and regulatory demands may be one reason, and the other may be due to the different biases which are introduced when dividing the genome-scale metabolic networks into target subsystems and surrounding subsystems (see Discussion). The sequences of internal EqSets with the lowest *p*-values are shown in S3, S4 and S5 Tables for the above three subnetworks, respectively. Most EqSets in the top part of the sequences include at least one regulated reaction.

**Table 5:** The span of the four parameters required in EqSet sequence calculation.

Parameter	Span
*μ*	0, 0.025, 0.05, 0.075, 0.1, 0.125, 0.15, 0.175, 0.2, 0.25, 0.3, 0.35, 0.4, 0.45, 0.5, 0.6, 0.7, 0.8, 0.9, 1
*ρ*_*e*_	1, 2, 3, 4, 5, 6, 7, 8, 9, 10, 11, 12, 14, 16, 18, 20, 22, 24, 28, 32, 36, 40, 44, 48, 52, ∞
*ρ*_*s*_	1, 2, 3, 4, 5, 6, 7, 8, 9, 10, 11, 12, 14, 16, 18, 20, 22, 24, 28, 32, 36, 40, 44, 48, 52, ∞
*τ*	1, 2, 3, 4, 5, 6, 7, 8, 9, 10, 11, 12, 13, 14, 15, 16, 17, 18, 19, 20, 22, 24, 26, 28, 30, 32, 34, 36, 38, 40

**Table 6:** Summary of the distribution of *p*-values corresponding to the EqSet sequences calculated on the candidate parameter values. The two right-most columns show the proportion of candidate parameter values that result in an EqSet sequence with *p*-value less than 0.05 or 0.01, respectively. Min, minimum; Max, maximum.

Subnetwork	Min.	Max.	Median	Average	Variance	*p* < 0.05	*p* < 0.01
Amino acid metabolism (*E. coli*)	3.10 × 10^−4^	0.9778	0.0266	0.1076	0.1924	62.07%	7.27%
Hydrocarbon metabolism (*E. coli*)	4.78 × 10^−6^	0.9943	0.0232	0.1207	0.2389	78.94%	29.90%
Lipid metabolism (*E. coli*)	2.08 × 10^−9^	0.7448	7.19 × 10^−6^	0.0098	0.0456	96.13%	90.83%
Amino acid metabolism (human)	1.41 × 10^−7^	0.7621	5.37 × 10^−4^	0.0185	0.0047	91.93%	86.83%
Hydrocarbon metabolism (human)	7.33 × 10^−6^	0.7892	0.0665	0.0830	0.0049	35.42%	5.73%

**Fig 6:**
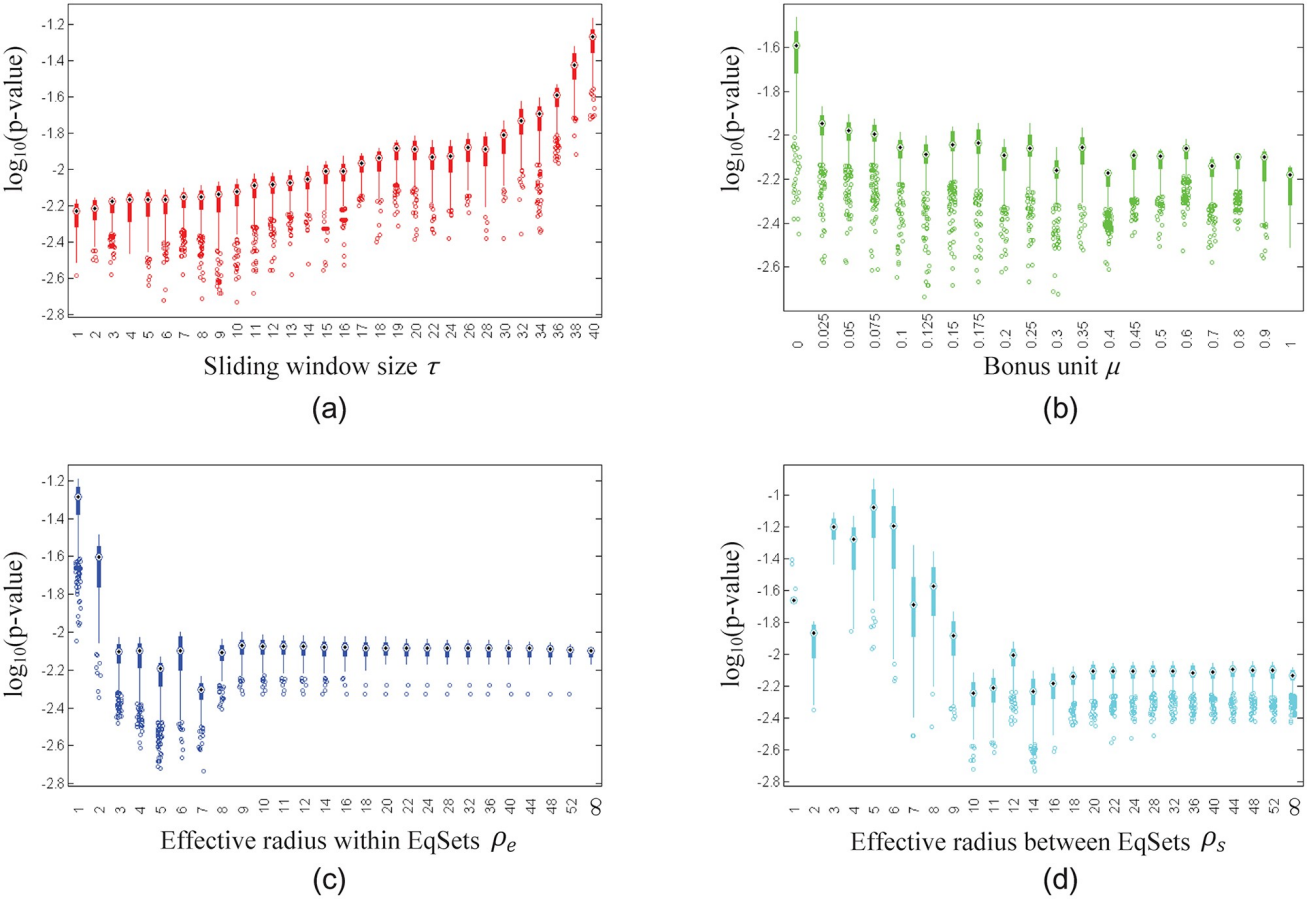
Box plots show the distribution of the lowest 5% of the *p*-values of the EqSet sequences of amino acid metabolism in *E. coli* when the parameter specified by the label of each subgraph is fixed to the value under the box. The ordinate axis is plotted in log scale.

**Fig 7:**
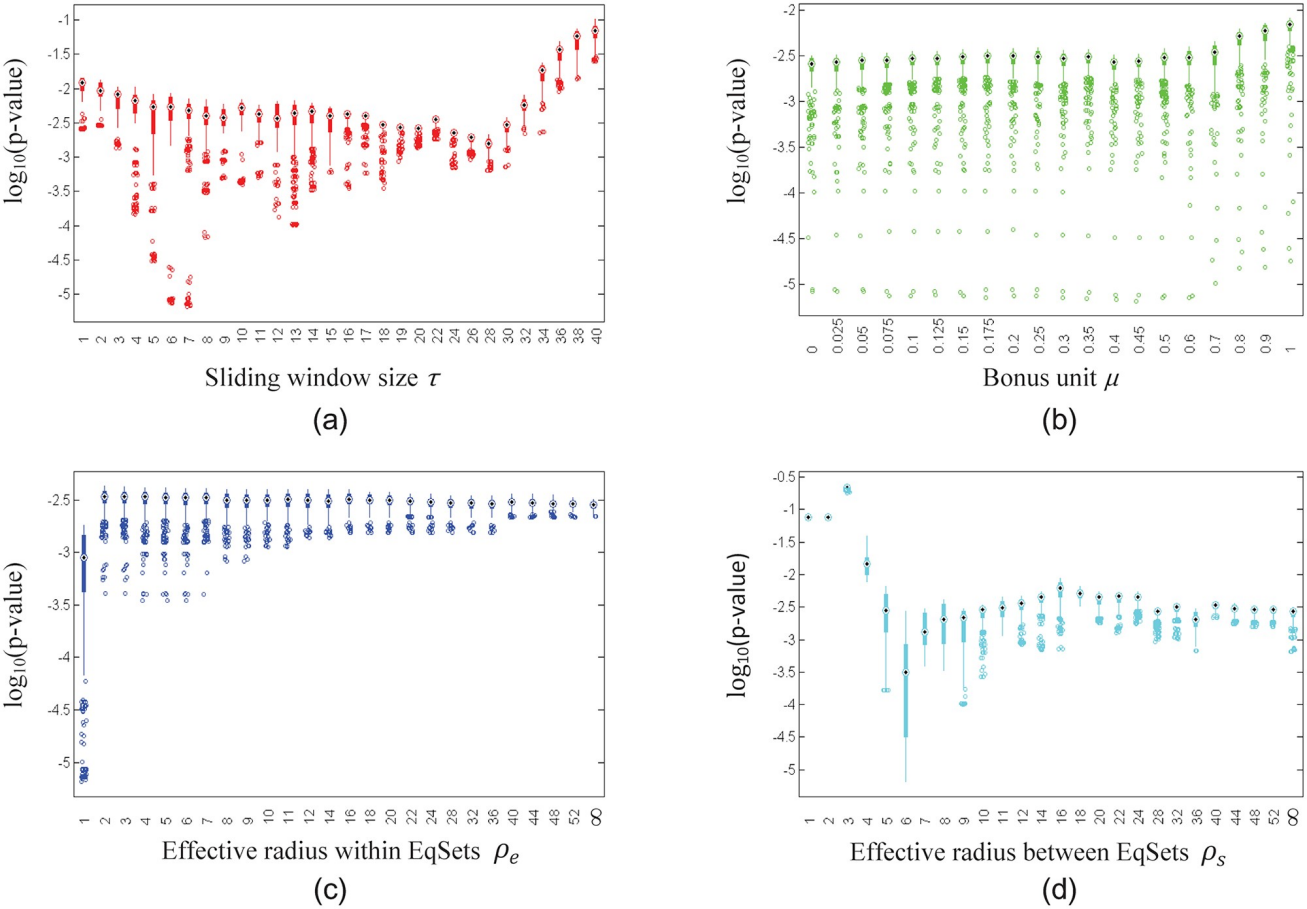
Box plots show the distribution of the lowest 5% of the *p*-values of the EqSet sequences of hydrocarbon metabolism of *E. coli* when the parameter specified by the label of each subgraph is fixed to the value under the box. The ordinate axis is plotted in log scale.

**Fig 8:**
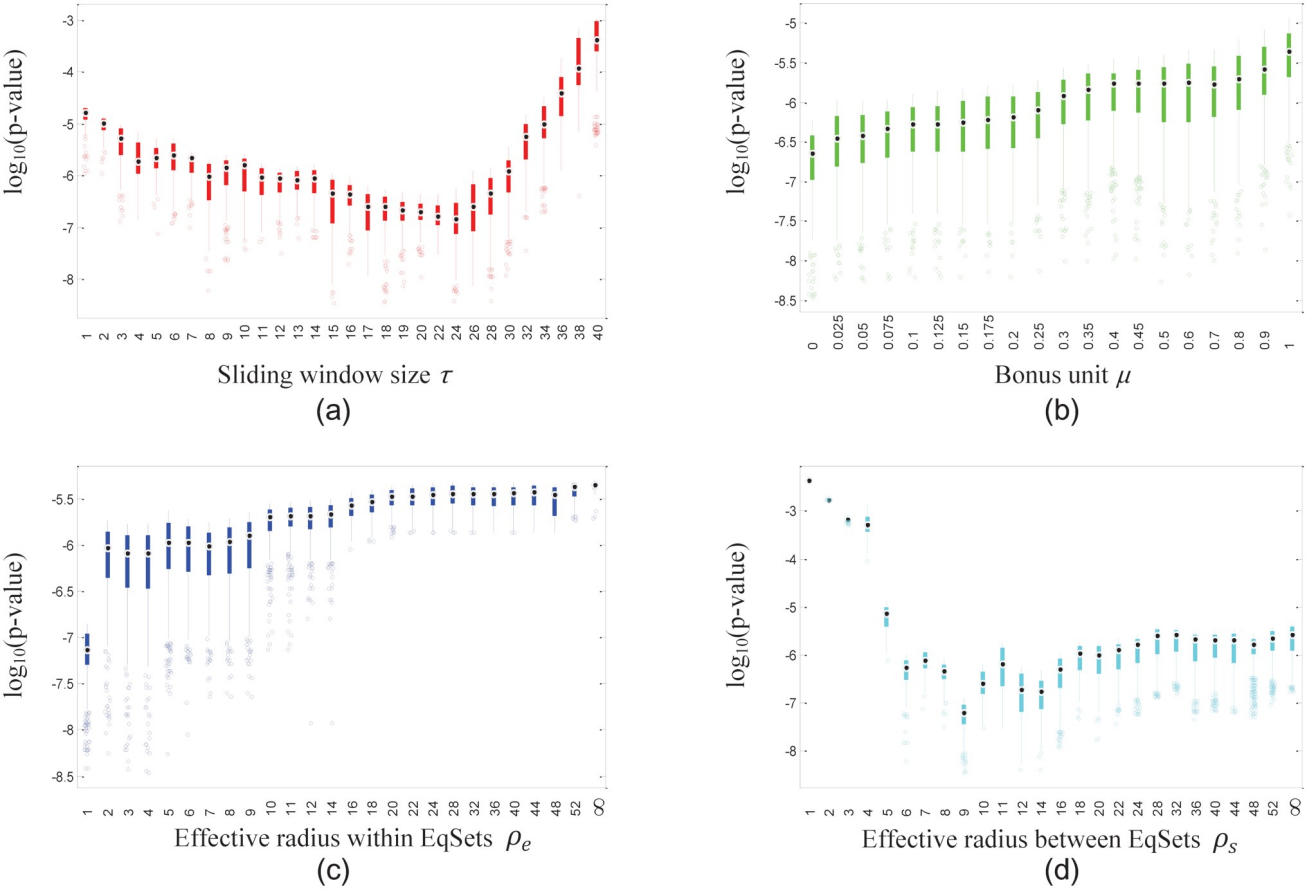
Box plots show the distribution of the lowest 5% of the *p*-values of the EqSet sequences of lipid metabolism of *E. coli* when the parameter specified by the label of each subgraph is fixed to the value under the box. The ordinate axis is plotted in log scale.

In order to take a closer look at the role of each parameter, we explored the distributions of the lowest 5% of the *p*-values when one of the parameters was fixed and the others were not (Figs 6, 7 and 8 for the subnetworks of amino acid metabolism, hydrocarbon metabolism and lipid metabolism, respectively). In general, the distribution of the lowest 5% of the *p*-values shows stable trends when the value of the fixed parameter ranges from the minimum to the maximum in the scope. The parameter *μ* has a slight effect on the *p*-values compared to the effects of parameters *ρ*_*e*_, *ρ*_*s*_ and *τ*. For different subnetworks, the *p*-values drop to a low value at different values of *ρ*_*e*_, *ρ*_*s*_ and *τ*, which implies that the evolutionary demands of metabolic regulation diverge slightly for different functional parts.

### Predicting disease-associated reactions in the human metabolic network

Metabolic disorders have been linked with chronic disease processes, including heart disease, cancer, diabetes, and obesity [66]. Since the reactions of high importance for metabolic regulation play a key role in controlling a phenotype shift, their malfunction may induce a morbid state of the organism. So it is reasonable to infer that the reactions with the most regulatory importance are more likely to be associated with disease processes. We applied our methods to the genome-scale human metabolic network (Recon 1) [51] to predict disease-associated reactions.

The model H. sapiens Recon 1 of human metabolism accounts for 1,496 genes and 3,742 reactions, of which 431 are exchange reactions [51]. The disease-associated reactions were obtained from the public data of Lee et al [67]. The number of disease-associated reactions is 779, details are listed in S2 Table. As with our analysis of the *E. coli* metabolic network, we defined two subnetworks that respectively represent amino acid metabolism and hydrocarbon metabolism. The subsystems contained in each subnetwork are listed in Table 7 and other related information is summarized in Table 4.

**Table 7:** Subsystems contained in each metabolic sub network of human.

Subnetwork	Subsystems
Amino acid metabolism	Alanine and aspartate metabolism; aminosugar metabolism; arginine and proline metabolism; citric acid cycle; CoA biosynthesis; CoA catabolism; cysteine metabolism; D-alanine metabolism; folate metabolism; glutamate metabolism; glutathione metabolism; glycine, serine, and threonine metabolism; heme biosynthesis; heme degradation; histidine metabolism; lysine metabolism; methionine metabolism; phenylalanine metabolism; salvage pathway; taurine and hypotaurine metabolism; tetrahydrobiopterin; tryptophan metabolism; Tyr, Phe, Trp biosynthesis; tyrosine metabolism; urea cycle/amino group metabolism; valine, leucine, and isoleucine metabolism; vitamin b6 metabolism; beta-alanine metabolism
Hydrocarbon metabolism	Ascorbate and aldarate metabolism; biotin metabolism; CYP metabolism; citric acid cycle; CoA biosynthesis; CoA catabolism; folate metabolism; miscellaneous; fructose and mannose metabolism; NAD metabolism; glycolysis/gluconeogenesis; galactose metabolism; glyoxylate and dicarboxylate metabolism; hyaluronan metabolism; IMP biosynthesis; keratan sulfate degradation; N-glycan biosynthesis; unassigned; N-glycan degradation; oxidative phosphorylation; pentose phosphate pathway; pyruvate metabolism; pentose and glucuronate interconversions; putative; propanoate metabolism; riboflavin metabolism; salvage pathway; starch and sucrose metabolism; thiamine metabolism; oxidative phosphorylation; pentose phosphate pathway

**Fig 9:**
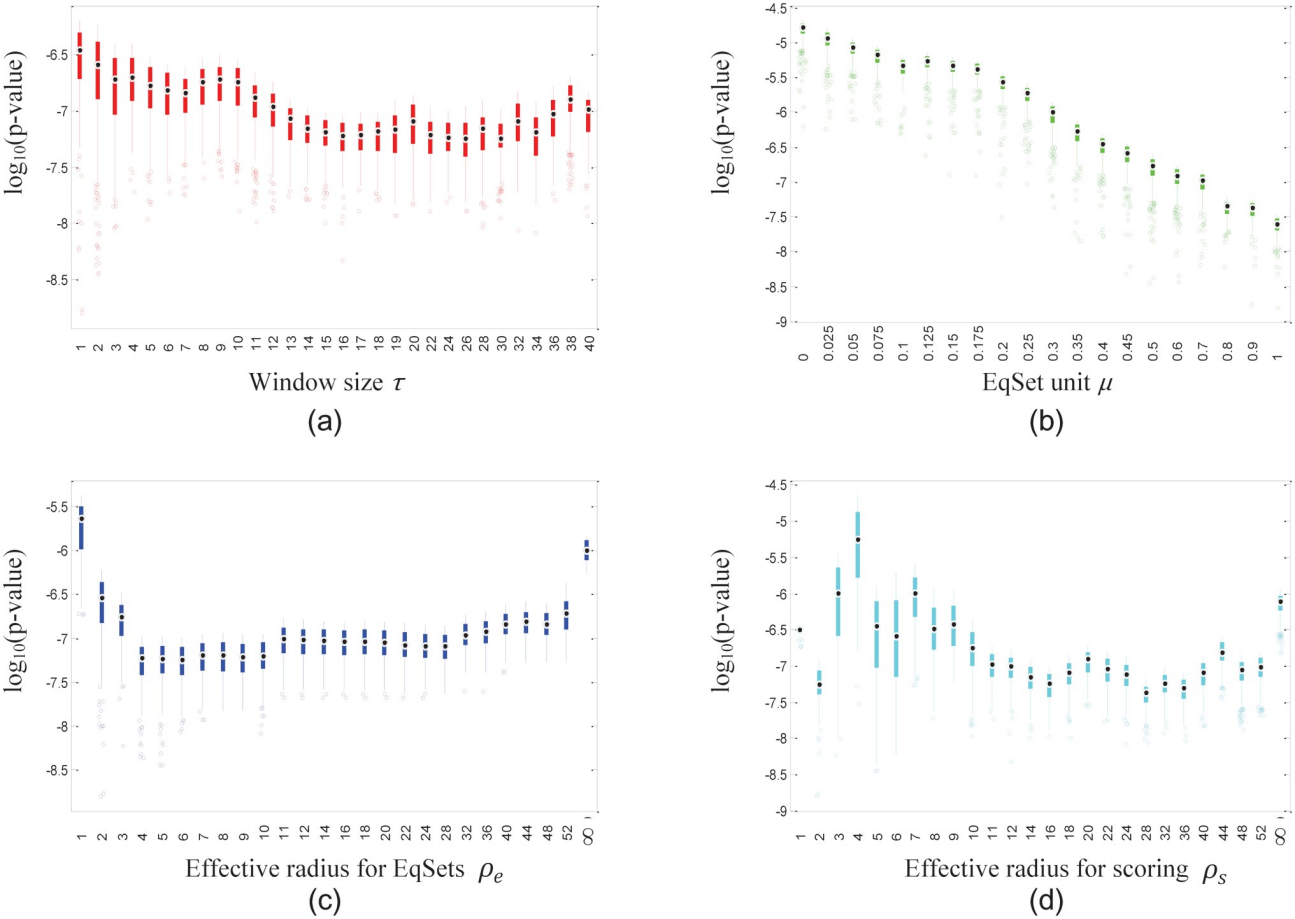
Box plots show the distribution of the lowest 5% of the *p*-values of the EqSet sequences of human amino acid metabolism when the parameter specified by the label of each subgraph is fixed to the value under the box. The ordinate axis is plotted in log scale.

**Fig 10:**
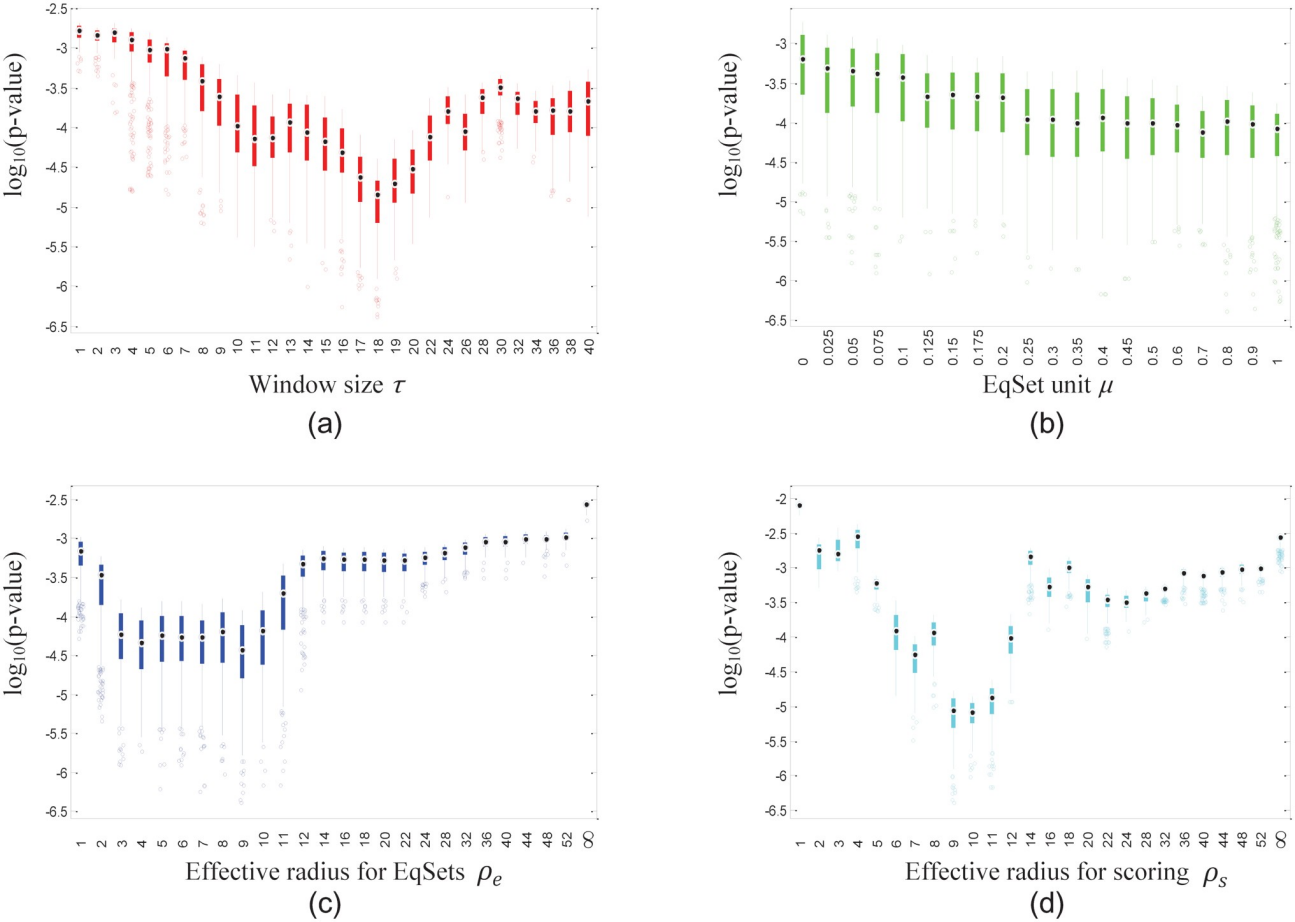
Box plots show the distribution of the lowest 5% of the *p*-values of the EqSet sequences of human hydrocarbon metabolism when the parameter specified by the label of each subgraph is fixed to the value under the box. The ordinate axis is plotted in log scale.

We made sequences of EqSets with the same candidate set of parameter values as that of the *E. coli* model, and then calculated the corresponding *p*-values to see whether the disease-associated reactions tend to rank higher than the reactions that are not associated with disease processes. That tendency is significant for a large section of EqSet sequences (Table 6), especially for the subnetwork of human amino acid metabolism, of which most sequences get *p*-values less than 0.05. Similar to the situations we found for hRBCs and *E. coli*, the distributions of the lowest 5% of the *p*-values also showed stable trends with changes in each fixed parameter (Figs 9 and 10). The results suggest that disease-associated reactions may be discovered by evaluating their regulatory importance.

The sequences with the lowest *p*-values are shown in S6 and S7 Tables for the above two subnetworks, respectively, whose front parts are enriched with disease-associated reactions. After a literature study of the internal EqSets which stand in the top 30 of the sequences but are not disease-associated according to Lee’s data [67], we found it worth mentioning that most of their member reactions were reported to be related to some diseases by other published papers (Table 8). Therefore, our approach of sorting reactions according to their regulatory importance is also capable of predicting the potential reactions which are related with some diseases.

**Table 8:** Non-disease-associated internal EqSets in top 30 of the sequences of the human amino acid or hydrocarbon metabolic networks with the lowest p-value. The reaction abbreviation and reaction name look-up table is listed in Table F in S1 Text.

EqSet	Rank	Disease-related reaction	Related diseases	References
*Human amino acid metabolism*				
3HAO, HKYNH, KYN3OX	13	3HAO	pellagra, olivopontocerebellar atrophy	[68, 69]
MTHFCm	24	MTHFCm	myelomeningocele, spina bifida	[70–72]
MTHFD2	26	MTHFD2	lung cancer, spina bifida, breast cancer	[73–76]
FKYNH, TRPO2	29	TRPO2	pellagra, haemophilus influenzae	[77]
GHMT2r	30	GHMT2r	adult acute lymphocytic leukemia, pediatric osteosarcoma	[78, 79]
*Human hydrocarbon metabolism*				
NMNS	9	NMNS	idiopathic recurrent pericarditis, gestational diabetes	[80–86]
NNDPR, EX_Sub_quln[c]	11	NNDPR	pellagra, follicular thyroid carcinoma	[87]
DOLGLCP_Lter, DOL_GPP_Ler, UDPDOLPT_L	12	-	-	-
DOLGLCP_Uter, DOL_GPP_Uer, UDPDOLPT_U	14	-	-	-
GAPD	19	GAPD	diffuse large B-cell lymphoma, obesity	[88, 89]
RBK, EX_Sub_rib-D[c]	27	-	-	-
FPGS4	28	FPGS4	Rheumatoid arthritis, psoriasis, colorectal cancer, non-small-cell lung cancer, non-Hodgkin lymphoma	[90–95]

**Fig 11:**
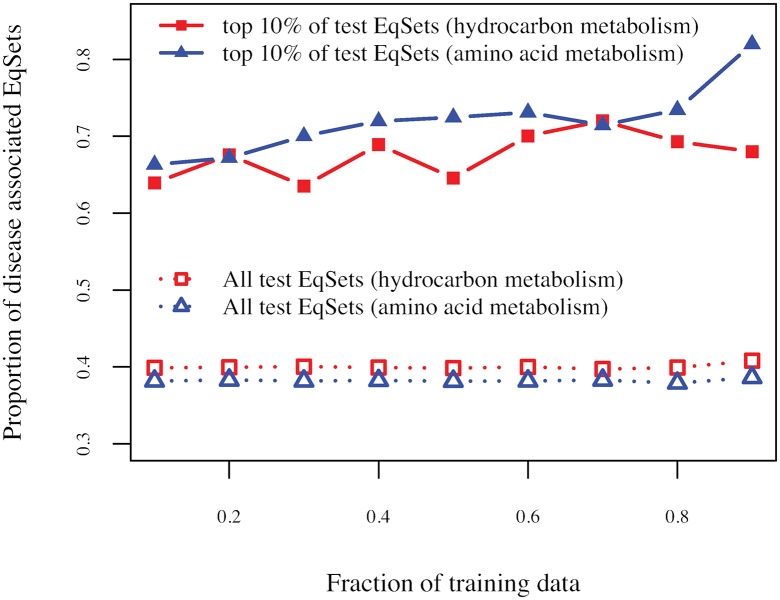
The average true positive rate versus the background proportion of a disease-associated EqSet. The average true positive rate equals the proportion of disease-associated EqSets among those that participate in the top 10% of the sequence composed of the test EqSets. The background proportion equals the ratio of disease-associated EqSets among all the test EqSets. The horizontal axis represents the fraction of EqSets that used as training data.

In order to test whether the regulatory importance is a informative feature in detecting the disease-associated reactions, we divided the reactions of a subnetwork into a training set and a test set. The parameter values that fit the training set best, i.e., minimized the average *p*-value of the training set, were used to predict the disease-associated reactions in the test set. In the top 10% of the resulting sequences consisting of test EqSets, the average ratio of internal EqSets containing disease-associated reactions increased by 23% to 33% compared with that of random guessing, depending on the proportion of the EqSets used as training data (Fig 11). The accuracy of the prediction is significantly higher than random guessing, even if fewer disease-associated reactions are known (i.e., the proportion of the training set is low). Thus, the regulatory importance determined from extreme pathways can be a valuable new feature that contributes to the prediction of disease-associated reactions.

### Quick search for suitable parameters

**Fig 12:**
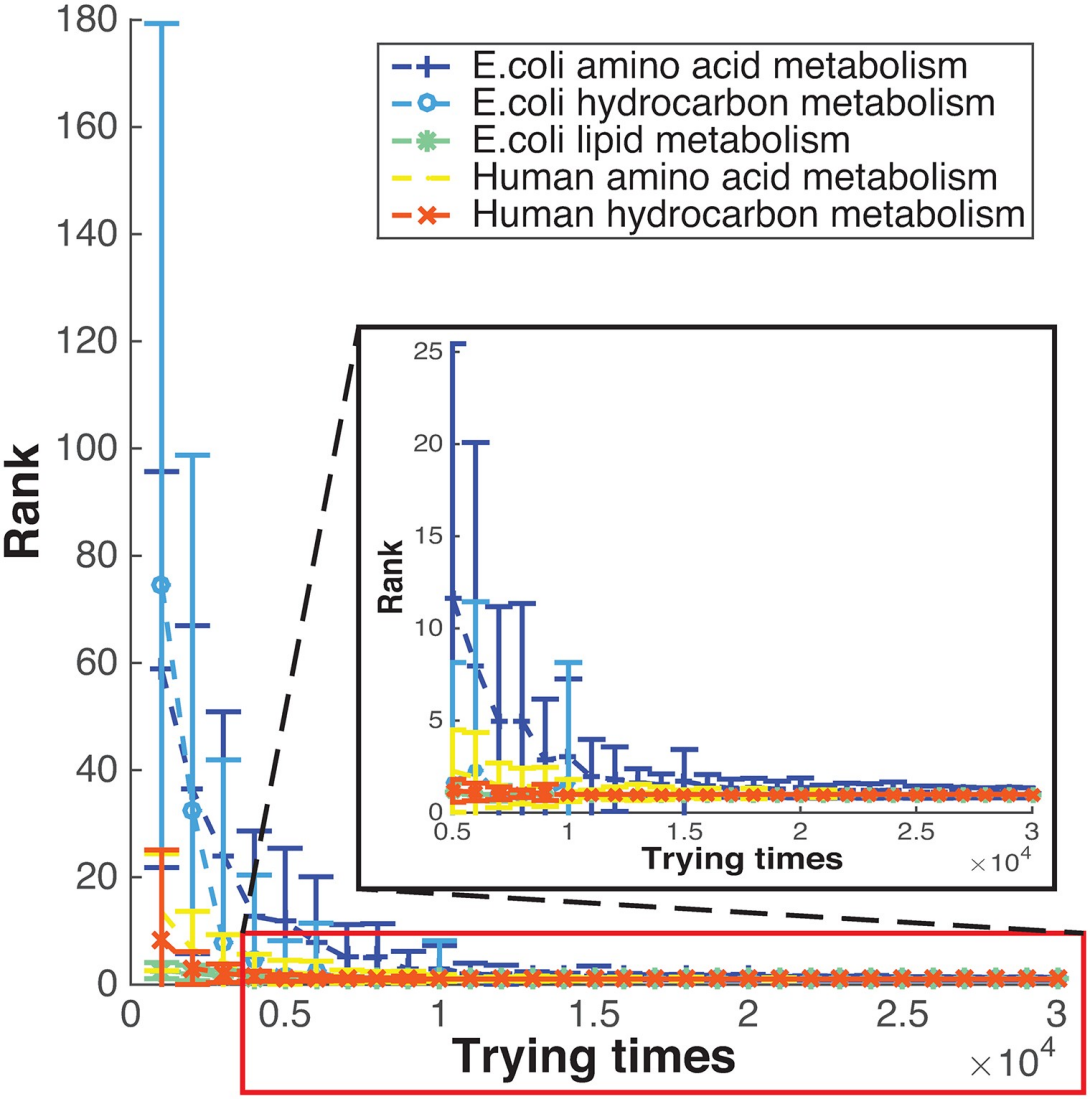
The heuristic parameter searching algorithm quickly identified the best or nearly best among all the candidates after a few attempts. The horizontal axis represents the number of attempts in which an EqSet sequence is calculated on certain values of the 4 parameters. The number of attempts ranges from 1,000 to 50,000, with a step of 1,000. The vertical axis represents the number of the candidate parameter values for which the resulting *p*-values are no higher than those of the parameters suggested by the search algorithm. The algorithm was repeated 100 times at each attempt. The average ranks are shown by the line marks and standard deviations are shown with the error bars. The boxed off portion of the graph is zoomed in as a separate one to show more details.

In the above examples, we tried over 200,000 different values of the parameters to find the best fit, which was so time-consuming that it may restrict the practical use of our methods. Therefore, an efficient approach to parameter optimization is extremely useful. Respecting the smooth and nearly single-trough distributions of the lowest *p*-values for each parameter, we improved the parameter search by use of a heuristic algorithm. The heuristic algorithm achieved the best or nearly best parameter values in the candidate set after trying 5,000 to 20,000 candidates (Fig 12); thereby providing a more than 90% time saving improvement compared with an exhaustive search.

## Conclusion and discussion

Metabolism provides cells with energy and building blocks needed to produce biological structures, maintain the cell as well as carry out various cellular functions [66]. An elaborate regulatory structure enables the cell to adapt to a variety of internal and external perturbations. The organizing principle of the regulation of cellular metabolism remains a fundamental problem in biology. Previous research has focused on the regulatory patterns of metabolic reactions under certain perturbations of a cell’s internal and environmental states. In this paper, we present a new approach for predicting the regulatory architecture of a cell’s metabolism. The prediction is done by sorting the reactions according to their regulatory importance, which is measured based on the ratios of regulatory extreme pathways. By applying the method to the metabolic networks of the human erythrocyte and *E. coli*, we found that the regulation of metabolism prefers reactions that are efficient and flexible in controlling the extreme pathways.

Our study sheds light on the possible mechanism whereby the regulatory architecture of a metabolic system is determined. It also supports the hypothesis that a cell transforms from one metabolic steady-state to another by switching “on/off” certain extreme pathways [36]. This implies that it is the extreme pathways, which are encoded in the stoichiometric relationship of a metabolic network, that are the real targets of metabolic regulation. In the process of evolution, metabolic reactions have acquired the ability to exert control by various means, such as the allosteric and transcriptional regulation of enzymes and genes, respectively. Reactions that play a key role in regulation are reserved as regulated reactions and form the evolutionary context in which the natural selection for the next regulated reaction occurs.

Our approach is context-free, meaning that it requires no information about the specific perturbations in the internal physiological states or environmental conditions of the cell. Rather, it concerns the whole space of valid metabolic steady-states and selects reactions that are important for regulation on average under various circumstances. Since the metabolic network and regulation strategies occurring in a cell are the result of an evolutionary process that arose under complicated and changeable internal and environmental conditions, our approach may provide general principles and closer approximations of the development of the regulatory structure of metabolism. The regulatory structure can be used as a blueprint, which provides the information about where metabolic regulation may occur, for context-dependent methods that aim to discover the regulatory strategies for certain perturbations. The combination of both context-free and context-dependent approaches may not only decrease the complexity of the problem, but also increase the quality of the answer.

Moreover, we demonstrated the ability of our approach to predict disease-associated reactions in human metabolic networks. According to the regulatory importance, we successfully detected the reactions whose mistuning or malfunction will lead to disease. In comparison with previous constraint-based reconstruction and analysis (COBRA) methods that predict lethal reactions, such as gene deletion analysis [96], the pathogenesis concerned in our study is not confined to the loss of enzymes, but also includes the loss of metabolic regulation. Gene deletion analysis requires the definitions of a cell’s growing condition as well as its metabolic objective as represented by an objective function. The metabolic objective in many situations is unknown and moreover changes for different organisms, cell types, environmental conditions, or internal physiological states. Therefore, it is difficult to define the objective function, which prevents the practical use of gene deletion analysis. In contrast, our approach avoids that obstacle by using extreme pathway analysis. The prediction of disease-associated reactions can be used with other data, such as gene function and sequence variation, to screen out mutations closely related to diseases. In addition to its application to study disease processes, our approach can also be used in metabolic engineering and synthetic biology to inform the optimal control nodes (reactions of most regulatory importance) in metabolic networks.

It is worth to note that the consideration of only subsystems of genome-scale metabolic networks is a trade-off between computational costs and accuracy. Although dividing the global metabolic network to a target subsystem and a surrounding subsystem does not change the space of steady states of the global system, constraining the analysis to the target subsystem does impact the extreme pathways detected [97, 98]. For example, some of the possible pathways of the global metabolic network will be missed [97] and interdependencies between pairs of fluxes may be lost [98]. Therefore the frequency of reaction participation in extreme pathways may be changed and some EqSets may be disrupted. As a result the predicted regulatory importance of reactions will be biased. In the previous study [97], Kaleta et al. suggested using another valuable concept of elementary flux patterns, which explicitly takes into account possible steady-state fluxes through the global metabolic network, instead of elementary modes or extreme pathways. However elementary flux patterns can not solve our problem because the factor that ‘each elementary flux pattern can be mapped to at leaset one elementary mode’ in the global system [97] may introduce even more dramatical bias on the frequency of reaction participation in metabolic pathways. In this study, we reduce the above bias by optimizing the partition of the target subsystem. Our practice shows that it is feasible to divide the global metabolic network by reactions’ functions. On the contrary, a target subsystem consisting of randomly selected reactions always resulted poor predictions of reactions’ regulatory importance. However, an obvious disadvantage of function based partition is that the resulted subsystems are usually biased by the partitioner’s knowledge of each reaction. An important part of the future work is to develop an algorithm to produce a reasonable partition of a genome-scale metabolic network. The aim of the algorithm is to introduce as few exchange reactions as possible while ensuring the complexity, i.e., the number of reactions, of the target subnetwork since abstracting the surrounding subsystem into exchange reactions is the main cause of the bias of extreme pathways detected in the target subsystem [97, 98].

Another challenge for the future work is to integrate omics data, such as transcriptomic and proteomic data, to refine our evaluation of the regulatory cost of each reaction, which will help improve the prediction quality of our approach. Valuable improvements will include determining the most possible regulated reactions in an EqSet, and increasing the agreement between the predicted EqSet sequence and the real sequence that occurs in nature. Furthermore, a quantitative estimation of the probability or confidence level that a reaction is regulatory or disease-associated is also in demand.

## Supporting information

S1 Text Supplementary methods and results. (PDF)

S1 Table The reaction abbreviations and reaction names of E.coli metabolic network and the information whether a reaction is regulatory or not. (XLSX)

S2 Table The reaction abbreviations and reaction names of human metabolic network and the information whether a reaction is disease-associated or not. (XLSX)

S3 Table The sequence of internal EqSets with the lowest p-value for the sub network of E.coli amino acid metabolism. P-value and the paramters for the sequence are listed at the end of the table.
(XLSX)

S4 Table The sequence of internal EqSets with the lowest p-value for the sub network of E.coli hydrocarbon metabolism. P-value and the paramters for the sequence are listed at the end of the table.
(XLSX)

S5 Table The sequence of internal EqSets with the lowest p-value for the sub network of E.coli lipid metabolism. P-value and the paramters for the sequence are listed at the end of the table.
(XLSX)

S6 Table The sequence of internal EqSets with the lowest p-value for the sub network of human amino acid metabolism. P-value and the paramters for the sequence are listed at the end of the table.
(XLSX)

S7 Table The sequence of internal EqSets with the lowest p-value for the sub network of human hydrocarbon metabolism. P-value and the paramters for the sequence are listed at the end of the table.
(XLSX)
