# Supplementary material for: Extreme pathway analysis reveals the organizing rules of metabolic regulation
Source: PLoS One. 2019 Feb 5;14(2):e0210539. doi: 10.1371/journal.pone.0210539 (PMC6363282; doi:10.1371/journal.pone.0210539)
Supplement: S1 Text — (PDF) [file pone.0210539.s001.pdf]

Supplement to: **Extreme Pathways Affect the Regulatory Topology of Metabolic Systems**

Yanping Xi[1,2,3] and Fei Wang[1,2*]

[1]Shanghai Key Lab of Intelligent Information Processing, Fudan University, Shanghai, China, [2]School of Computer Science and Technology, Fudan University, Shanghai, China, [3]Shanghai Ji Ai Genetics & IVF Institute, Obstetrics and Gynecology Hospital of Fudan University, 588 Fangxie Rd, Shanghai, China

Supplementary Notes

Converting an extreme pathway to a compact extreme pathway

Given a metabolic network composed of $u^{irr}+u^{rev}+w^{irr}+w^{rev}$ reactions, where u^{irr}, u^{rev}, $\mathrm{w}^{\mathrm{irr}}$ and w^{rev} are the respective number of irreversible internal reactions, reversible internal reactions, irreversible exchange reactions and reversible exchange reactions in the metabolic network, suppose the sequence of the reactions occurs in the order given above, then the network's stoichiometric matrix $\mathbf{S}$ can be augmented to $\hat{\mathbf{S}}$ as follows: [1]

$$\hat{\mathbf{s}}^i=\mathbf{s}^i,\ \ i=1,2,\cdots,u^{\mathrm{irr}}$$
$$\hat{\mathbf{s}}^{u^{\mathrm{irr}}+2(i-u^{\mathrm{irr}})-1}=\mathbf{s}^i,\ \ \hat{\mathbf{s}}^{u^{\mathrm{irr}}+2(i-u^{\mathrm{irr}})}=-\mathbf{s}^i,\ \ i=u^{\mathrm{irr}}+1,\cdots,u$$
$$\hat{\mathbf{s}}^{u^{\mathrm{rev}}+i}=\mathbf{s}^i,\ \ i=u+1,\cdots,u+w,$$

where $\mathbf{s}^i$ and $\hat{\mathbf{s}}^i$ denote the ith column of $\mathbf{S}$ and $\hat{\mathbf{S}}$, respectively, $u=u^{irr}+u^{rev}$, and $w=w^{irr}+w^{rev}$.

The extreme pathways of the metabolic network are the extreme rays of the polyhedral cone: [1]

$$\hat{\mathbb{V}}=\{\hat{\mathbf{v}}\,|\,\hat{\mathbf{S}}\hat{\mathbf{v}}=0 \text{ and } \hat{v}_i\geq 0, i=1,\cdots,u^{irr}+2u^{rev}+w^{irr}\},$$

where $\hat{\mathbf{v}}=[\,\hat{v}_1,\hat{v}_2,\cdots,\hat{v}_{\mathrm{u+u^{rev}+w}}]$, $\hat{v}_i$ represents the flux of the ith irreversible internal reaction on the extreme pathway when $\mathrm{i}=1,2,\cdots,u^{irr}$, $\hat{v}_{u^{irr}+2(i-u^{irr})-1}$ and $\hat{v}_{u^{irr}+2(i-u^{irr})}$ represent the flux in the forward and backward directions of the $(\mathrm{i}-u^{irr})$th reversible internal reaction when $i=u^{irr}+1,\cdots,u^{\mathrm{irr}}+u^{\mathrm{rev}}$, and $\hat{v}_{u^{rev}+i}$ represents the flux of the $(i-u^{\mathrm{irr}}-u^{\mathrm{rev}})$th exchange reaction when $i=u^{\mathrm{irr}}+u^{\mathrm{rev}}+1,\cdots,u^{\mathrm{irr}}+u^{\mathrm{rev}}+w^{\mathrm{irr}}+w^{\mathrm{rev}}$. The extreme pathways generate the polyhedral cone, meaning that any vector in the cone can be represented as a convex combination of the extreme pathways. And they are systematically independent, meaning that they cannot be decomposed into a non-trivial convex combination of any other vectors residing in the cone. The set of extreme pathways of $\hat{\mathbb{V}}$ is unique.

Given an extreme pathway $\mathbf{p}^k=[p_1^k,p_2^k,\ldots,p_{u^{irr}+2u^{rev}+w^{irr}+w^{rev}}^k]$, the reaction r_i is part of $\mathbf{p}^k$, denoted as $\mathrm{r}_i\in\mathbf{p}^{\mathrm{k}}$, if and only if

$$\begin{cases} p_i^k > 0 & i = 1,\cdots,u^{irr} \\ p^k_{u^{irr}+2(i-u^{irr})-1} > 0 \; or \; p^k_{u^{irr}+2(i-u^{irr})} > 0 & i = u^{irr}+1,\cdots,u^{irr}+u^{rev} \\ p^k_{u^{rev}+i} \neq 0 & i = u^{irr}+u^{rev}+1,\cdots,u^{irr}+u^{rev}+w^{irr}+w^{rev} \end{cases}$$

The two elements respectively representing the fluxes along the forward and backward directions of a reversible internal reaction in $\mathbf{p}^k$ are merged together by subtracting the reverse flux from the forward one, which results in the compact extreme pathway $\mathbf{y}^k$. Formally, $\mathbf{y}^k = [\mathbf{y}_1^k, \mathbf{y}_2^k, \ldots, \mathbf{y}^k_{u^{\mathrm{irr}}+u^{\mathrm{rev}}+w^{\mathrm{irr}}+w^{\mathrm{rev}}}]$, where

$$y_i^k = \begin{cases} p_i^k & i = 1,\cdots,u^{irr} \\ p^k_{u^{irr}+2(i-u^{irr})-1} - p^k_{u^{irr}+2(i-u^{irr})} & i = u^{irr}+1,\cdots,u^{irr}+u^{rev} \\ p^k_{u^{rev}+i} & i = u^{irr}+u^{rev}+1,\cdots,u^{irr}+u^{rev}+w^{irr}+w^{rev} \end{cases}$$

The elements in y^k have a one-to-one correspondence with the reactions in the metabolic network. The reaction $\mathrm{r_i}$ is part of $\mathbf{y}^k$, which is denoted as $\mathrm{r}_i \in \mathbf{y}^k$, if and only if $y_i^k \neq 0$.

Statement A. Let $\hat{\mathbf{S}}$ be a matrix of $\boldsymbol{k}$ rows and $(\boldsymbol{n}+\boldsymbol{l})$ columns, and $\widehat{\mathbb{V}}$ be a polyhedral cone such that $\widehat{\mathbb{V}} = \{\hat{\mathbf{v}} | \hat{\mathbf{S}}\hat{\mathbf{v}} = \mathbf{0} \text{ and } \hat{\boldsymbol{v}}_i \geq \mathbf{0}, \boldsymbol{i} = \mathbf{1},\cdots,\boldsymbol{n}\}$. The zero elements of a vector $\hat{\mathbf{v}}$ are denoted as $\mathbb{E}(\hat{\mathbf{v}})$, formally $\mathbb{E}(\hat{\mathbf{v}}) = \{\boldsymbol{i} | \mathbf{1} \leq \boldsymbol{i} \leq \boldsymbol{n},\ \hat{\boldsymbol{v}}_i = \mathbf{0}\}$. If the nonzero vectors $\mathbf{u} = [\boldsymbol{u}_1,\cdots,\boldsymbol{u}_n,\boldsymbol{u}_{n+1},\cdots,\boldsymbol{u}_{n+l}]^\mathbf{T}$ and $\hat{\mathbf{v}} = [\hat{\boldsymbol{v}}_1,\cdots,\hat{\boldsymbol{v}}_n,\hat{\boldsymbol{v}}_{n+1},\cdots,\hat{\boldsymbol{v}}_{n+l}]^\mathbf{T}$ belong to $\widehat{\mathbb{V}}$ and $\mathbb{E}(\mathbf{u}) \subseteq \mathbb{E}(\hat{\mathbf{v}})$, then there exists a vector $\breve{\mathbf{u}} \in \widehat{\mathbb{V}}$ fulfilling the characteristic that $\mathbf{u}$ can be decomposed into a convex combination of $\breve{\mathbf{u}}$ **and** $\hat{\mathbf{v}}$, and $\mathbb{E}(\mathbf{u}) \subsetneq \mathbb{E}(\breve{\mathbf{u}})$, $\mathbb{E}(\breve{\mathbf{u}}) \nsubseteq \mathbb{E}(\hat{\mathbf{v}})$.

Proof. Without loss of generality, assume that there exists an integer m between 1 and n such that when $1 \leq i \leq m$, $\hat{v}_i > 0$ and when $m < i \leq n$, $\hat{v}_i = 0$. Then obviously there exists a positive number M such that when $\forall 1 \leq i \leq m$, $M \times \hat{v}_i > u_i$. Let $\check{\mathbf{v}}$ be the vector defined as $\check{\mathbf{v}} = M \times \hat{\mathbf{v}} = [M \times \hat{v}_1, \cdots, M \times \hat{v}_m, 0, \cdots, 0, M \times \hat{v}_{n+1}, \cdots, M \times \hat{v}_{n+l}]$; then obviously $\check{\mathbf{v}} \in \widehat{\mathbb{V}}$. Let $f(t)$ be the function such that $f(t) = t \times \mathbf{u} + (1-t) \times \check{\mathbf{v}} = t \times (\mathbf{u} - \check{\mathbf{v}}) + \check{\mathbf{v}}$, then $f(0) = \check{\mathbf{v}} \in \widehat{\mathbb{V}}, f(1) = \mathbf{u} \in \widehat{\mathbb{V}}$ and $f(t) \in \widehat{\mathbb{V}}$ where $t \in [0,1]$. Since $f(t)$ is a continuous function, $\exists\, t_0 > 1$ such that $f(t) \in \widehat{\mathbb{V}}$ when $1 < t \leq t_0$. Moreover, $\exists 1 \leq i_0 \leq m$ such that $t_0 \times (u_{i_0} - M \cdot \check{v}_{i_0}) + \check{v}_{i_0} = 0$. Let the vector $\breve{\mathbf{u}} = f(t_0)$, then $i_0 \in \mathbb{E}(\breve{\mathbf{u}})$. However, $i_0 \notin \mathbb{E}(\check{\mathbf{v}})$, where $\mathbb{E}(\check{\mathbf{v}}) = \mathbb{E}(\hat{\mathbf{v}})$, therefore $\mathbb{E}(\breve{\mathbf{u}}) \nsubseteq \mathbb{E}(\hat{\mathbf{v}})$ and $\mathbb{E}(\mathbf{u}) \subsetneq \mathbb{E}(\breve{\mathbf{u}})$。On the other hand, $\breve{\mathbf{u}} = f(t_0) = t_0 \times \mathbf{u} + (1-t_0) \times \check{\mathbf{v}} = t_0 \times \mathbf{u} + (1-t_0) \times M \times \hat{\mathbf{v}}$, so $\mathbf{u} = (1/t_0) \times \breve{\mathbf{u}} + (1 - 1/t_0) \times M \times \hat{\mathbf{v}}$, meaning that $\mathbf{u}$ can be decomposed into a convex combination of $\breve{\mathbf{u}}$ and $\hat{\mathbf{v}}$.□

Statement B. Let $\widehat{\mathbb{V}}$ be the polyhedral cone defined in Statement A. $\mathbb{A}$ is a set of a finite number of vectors in $\widehat{\mathbb{V}}$ and contains all the extreme pathways of $\widehat{\mathbb{V}}$. If the vectors $\mathbf{u}$ **and** $\mathbf{v}$ satisfy the conditions that $\mathbf{u}, \mathbf{v} \in \mathbb{A}$, $\mathbf{v} \neq \mathbf{0}$ **and** $\mathbb{E}(\mathbf{u}) \subseteq \mathbb{E}(\mathbf{v})$, then the set $\mathbb{A} - \{\mathbf{u}\}$ still contains all the extreme pathways of $\widehat{\mathbb{V}}$.

Proof. 1) By Statement A, $\exists \breve{\mathbf{u}} \in \mathbb{V}$ such that $\mathbb{E}(\mathbf{u}) \subsetneq \mathbb{E}(\breve{\mathbf{u}})$ and $\mathbf{u}$ can be decomposed into a convex combination of $\breve{\mathbf{u}}$ and $\mathbf{v}$. 2) As $\mathbb{A}$ contains all the extreme pathways of $\widehat{\mathbb{V}}$, $\breve{\mathbf{u}}$ can be decomposed into the convex combination of the vectors in $\mathbb{A}$. Since $\mathbb{E}(\mathbf{u}) \subsetneq \mathbb{E}(\breve{\mathbf{u}})$, $\breve{\mathbf{u}}$ can also be decomposed into the convex combination of the vectors in $\mathbb{A} - \{\mathbf{u}\}$. Combining 1) and 2) with the fact that $\mathbf{v} \in \mathbb{A} - \{\mathbf{u}\}$, it is obvious that $\mathbf{u}$ can be decomposed into the convex combination of the vectors in $\mathbb{A} - \{\mathbf{u}\}$. Therefore, $\mathbb{A} - \{\mathbf{u}\}$ still contains all the extreme pathways of $\widehat{\mathbb{V}}$. □

Statement C. Let $\mathbb{P}=\{\mathbf{p}^1,\mathbf{p}^2,\cdots,\mathbf{p}^{l_1},\mathbf{p}^{l_1+1},\mathbf{p}^{l_1+2},\cdots,\mathbf{p}^{l_2}\}$ be the set of extreme pathways of the polyhedral cone $\widehat{\mathbb{V}}=\{\hat{\mathbf{v}}|\mathbf{S}\hat{\mathbf{v}}=0 \text{ and } \hat{v}_i\geq 0, i=1,\cdots,u^{\mathrm{irr}}+2u^{\mathrm{rev}}+w^{\mathrm{irr}}\}$, where $l_1\leq l_2$, $\mathbf{p}^1,\mathbf{p}^2,\cdots,\mathbf{p}^{l_1}$ are of type I and II, and $\mathbf{p}^{l_1+2},\cdots,\mathbf{p}^{l_2}$ are of type III. Then, given $\forall\, i=1,\cdots,u^{\mathrm{rev}}$ and $\forall\, k$, $k=1,\cdots,l_1$, we have $p^k_{u^{\mathrm{irr}}+2i-1}\times p^k_{u^{\mathrm{irr}}+2i}=\mathbf{0}$.

Proof. If $\exists\, i_0$ and k satisfy the condition that $\mathbf{p}^k\in\mathbb{P}$ and $p^k_{u^{\mathrm{irr}}+2i_0-1}\times p^k_{u^{\mathrm{irr}}+2i_0}\neq 0$, where $i_0\in\{1,\cdots,u^{\mathrm{rev}}\}$, $k\in\{1,\cdots,l_1\}$, then we can construct the vector $\mathbf{p}^{l_2+1}$ as follows: First, assign $\mathbf{p}^k$ to $\mathbf{p}^{l_2+1}$ and then overwrite the value of $p^{l_2+1}_{u^{\mathrm{irr}}+2i_0-1}$ with $\max\left(p^k_{u^{\mathrm{irr}}+2i_0-1}-p^k_{u^{\mathrm{irr}}+2i_0},\ 0\right)$ and the value of $p^{l_2+1}_{u^{\mathrm{irr}}+2i_0}$ with $\max\left(p^k_{u^{\mathrm{irr}}+2i_0}-p^k_{u^{\mathrm{irr}}+2i_0-1},\ 0\right)$. Since $\mathbf{p}^k$ is a type I or II extreme pathway, there exists an integer j between $u^{\mathrm{irr}}+2u^{\mathrm{rev}}+1$ and $u^{\mathrm{irr}}+2u^{\mathrm{rev}}+w$ that satisfies the condition that $p^k_j\neq 0$. Correspondingly, $p^{l_2+1}_j\neq 0$, meaning that $\mathbf{p}^{l_2+1}\neq\mathbf{0}$. Besides, it is obvious that $\mathbf{p}^{l_2+1}\in\widehat{\mathbb{V}}$ and $\mathbb{E}(\mathbf{p}^k)\subseteq\mathbb{E}(\mathbf{p}^{l_2+1})$. By Statement B, $\mathbb{P}\cup\{\mathbf{p}^{l_2+1}\}-\{\mathbf{p}^k\}$ still contains all the extreme pathways in $\widehat{\mathbb{V}}$. By the uniqueness of $\mathbb{P}$, $\mathbf{p}^k\in\mathbb{P}$ is not an extreme pathway of $\widehat{\mathbb{V}}$, which conflicts with the assumption that $\mathbb{P}$ is a set of the extreme pathways of $\widehat{\mathbb{V}}$. Therefore, given $\forall\, i$, $i=1,\cdots,u^{\mathrm{rev}}$和$\forall\, k$, $k=1,\cdots,l_1$, we have $p^k_{u^{\mathrm{irr}}+2i-1}\times p^k_{u^{\mathrm{irr}}+2i}=0$。 □

Statement D. An extreme pathway of type I or II, $\mathbf{p}^{\boldsymbol{k}}$ and its compact form $\mathbf{y}^{\boldsymbol{k}}$ fulfill the condition that for any reaction $\mathbf{r}_{\boldsymbol{i}}$ $(\boldsymbol{i}=\mathbf{1},\cdots,\boldsymbol{u}+\boldsymbol{w})$, $\mathbf{r}_{\boldsymbol{i}}\in\mathbf{y}^{\boldsymbol{k}}$ if and only if $\mathbf{r}_{\boldsymbol{i}}\in\mathbf{p}^{\boldsymbol{k}}$.

Proof. $\mathrm{r}_i\in\mathbf{y}^k$ means $y^k_i\neq 0$. $\forall i\in\{1,\cdots,u^{\mathrm{irr}}\}$, since $y^k_i=p^k_i$, $y^k_i\neq 0$ if and only if $p^k_i\neq 0$. Given $\forall i\in\{u+1,\cdots,u+w\}$, since $y^k_i=p^k_{i+u^{rev}}$, $y^k_i\neq 0$ if and only if $p^k_{i+u^{rev}}\neq 0$. Given $\forall i\in\{u^{\mathrm{irr}}+1,\cdots,u^{\mathrm{rev}}\}$, since we have $p^k_{u^{\mathrm{irr}}+2(i-u^{\mathrm{irr}})-1}\times p^k_{u^{\mathrm{irr}}+2(i-u^{\mathrm{irr}})}=0$ by Statement C, $y^k_i\neq 0$ if and only if $\left(p^k_{u^{\mathrm{irr}}+2(i-u^{\mathrm{irr}})-1}>0 \text{ and } p^k_{u^{\mathrm{irr}}+2(i-u^{\mathrm{irr}})}=0\right)$ or $\left(p^k_{u^{\mathrm{irr}}+2(i-u^{\mathrm{irr}})-1}=0 \text{ and } p^k_{u^{\mathrm{irr}}+2(i-u^{\mathrm{irr}})}>0\right)$. Therefore, $\mathrm{r}_i\in\mathbf{y}^k$ if and only if $\mathrm{r}_i\in\mathbf{p}^k$. □

Thus, we proved that converting from an extreme pathway to its compact form does not change the set of reactions it utilized.

The synchronous nature of the member reactions in an EqSet

Two reactions in the same EqSet participate either always in the same extreme pathways or not at all since their pairwise conditional entropy equals 0. From Statement F, we found that the former case is the most common.

Statement E. Suppose G is a metabolic network, $\mathbb{P}$ is the set of extreme pathways of G, and G' is a subnetwork of G that is obtained by deleting a set of internal or irreversible exchange reactions $\mathbb{R}^{\mathrm{D}}$, then the vector set $\mathbb{P}'$, $\mathbb{P}' = \{\mathbf{p}|\mathbf{p} \in \mathbb{P} \text{ and } \forall\, \mathrm{r} \in \mathbb{R}^{\mathrm{D}},\ \mathrm{r} \notin \mathbf{p}\}$, is the set of the extreme pathways of G'.

Proof. Let $\mathbb{V}$ be the space of all the feasible steady states of the metabolic network G, $\mathbb{V} = \{\hat{\mathbf{v}}|\hat{\mathbf{S}}\hat{\mathbf{v}} = \mathbf{0}, \hat{v}_i \geq 0, i = 1, \ldots, n\}$. Without loss of generality, assume $\mathbb{R}^{\mathrm{D}} = \{\mathrm{r}_k, \mathrm{r}_{k+1}, \ldots, \mathrm{r}_n\}$, where k is an integer between 1 and n. Let $\mathbb{V}'$ be the space of all the feasible steady states of the metabolic subnetwork G', $\mathbb{V}' = \{\mathbf{v}'|\hat{\mathbf{S}}\hat{\mathbf{v}}' = \mathbf{0}, \begin{cases}\hat{v}_i' \geq 0 & \mathrm{i} = 1, 2, \ldots, k-1 \\ \hat{v}_i' = 0 & \mathrm{i} = k, k+1, \ldots, n\end{cases}\}$; in other words, $\mathbb{V}' = \{\hat{\mathbf{v}}'|\hat{\mathbf{v}}' \in \mathbb{V} \text{ and } \hat{v}'_i = 0, i = k, k+1, \ldots, n\}$. Since $\mathbb{P}' = \{\mathbf{p}|\mathbf{p} \in \mathbb{P} \text{ and } \forall\, \mathrm{r} \in \mathbb{R}^{\mathrm{D}}$, $\mathrm{r} \notin \mathbf{p}\}$, we have $\mathbb{P}' = \mathbb{P} \cap \mathbb{V}'$.

We can prove by contradiction that $\forall \hat{\mathbf{v}}' \in \mathbb{V}$, $\hat{\mathbf{v}}'$ can be decomposed into the convex combination of the vectors in $\mathbb{P}'$: Assume that $\exists \hat{\mathbf{v}}' \in \mathbb{V}'$, $\hat{\mathbf{v}}'$ cannot be decomposed into the convex combination of the vectors in $\mathbb{P}'$, as $\hat{\mathbf{v}}' \in \mathbb{V}' \subseteq \mathbb{V}$, $\hat{\mathbf{v}}'$ can be decomposed into the convex combination of the vectors in $\mathbb{P}$. Therefore, $\exists \alpha_1, \alpha_2, \cdots, \alpha_{|\mathbb{P}|}$, which leads to

$$\hat{\mathbf{v}}' = \sum_{i=1}^{|\mathbb{P}|} \alpha_i \mathbf{p}^i, \mathbf{p}^i \in \mathbb{P}, \forall i, \alpha_i \geq 0.$$

Since $\hat{\mathbf{v}}'$ cannot be decomposed into the convex combination of the vectors in $\mathbb{P}'$, there must be some i satisfying the condition that $\alpha_i > 0$ and $\mathbf{p}^i \in \mathbb{P} - \mathbb{P}' = \mathbb{P} - \mathbb{V}'$; in other words, there exist $k \leq j \leq n$ such that $p_j^i > 0$. Therefore, we have $\hat{v}_j' \geq \alpha_i p_j^i > 0$, $k \leq j \leq n$, which contradicts the fact that $\hat{\mathbf{v}}' \in \mathbb{V}'$. So $\forall \hat{\mathbf{v}}' \in \mathbb{V}'$, $\hat{\mathbf{v}}'$ can be decomposed into the convex combination of the vectors in $\mathbb{P}'$ such that $\mathbb{P}' = \mathbb{P} \cap \mathbb{V}'$. On the other hand, $\mathbb{P}$ is the set of extreme pathways of G, so the vectors in $\mathbb{P}$ are systematically independent. As $\mathbb{P}' \subseteq \mathbb{P}$, the vectors in $\mathbb{P}'$ are systematically independent as well. Moreover, $\mathbb{P}' \subseteq \mathbb{V}'$. Therefore $\mathbb{P}'$ is the set of extreme pathways of G'.

□

Statement F. Let $\mathbb{V}$ be the space of all the feasible steady states of a metabolic network, $\mathbb{V} = \{\mathbf{v}|\mathbf{Sv} = \mathbf{0} \text{ and } \boldsymbol{v}_i \geq \mathbf{0}, \boldsymbol{i} \in \{\mathbf{1}, \cdots, \boldsymbol{u}^{irr}, \boldsymbol{u}^{irr} + \boldsymbol{u}^{rev} + \mathbf{1}, \cdots, \boldsymbol{u}^{irr} + \boldsymbol{u}^{rev} + \boldsymbol{w}^{irr} + \boldsymbol{w}^{rev}\}\}$, $\mathbb{Y}$ be the set of compact extreme pathways of $\mathbb{V}$, $\boldsymbol{u} = \boldsymbol{u}^{\mathrm{irr}} + \boldsymbol{u}^{\mathrm{rev}}$ and $\boldsymbol{w} = \boldsymbol{w}^{\mathrm{irr}} + \boldsymbol{w}^{\mathrm{rev}}$. If $\exists\, \mathbf{1} \leq \boldsymbol{i}, \boldsymbol{j} \leq \boldsymbol{u}$, $\forall\, \mathbf{y} \in \mathbb{Y}$, $\boldsymbol{y}_i \neq \mathbf{0}, \boldsymbol{y}_j = \mathbf{0}$ or $\boldsymbol{y}_i = \mathbf{0}, \boldsymbol{y}_j \neq \mathbf{0}$, then $\{\mathbf{v}|\mathbf{v} \in \mathbb{V}, \boldsymbol{v}_i = \mathbf{0}, \boldsymbol{v}_j = \mathbf{0}, \exists\, \boldsymbol{u} + \mathbf{1} \leq \boldsymbol{t} \leq \boldsymbol{u} + \boldsymbol{w}, \boldsymbol{v}_t \neq \mathbf{0}\} = \emptyset$。

Proof. Let $\mathbb{V}'$ be a subset of $\mathbb{V}$, $\mathbb{V}' = \{\mathbf{v}|\mathbf{v} \in \mathbb{V} \text{ and } \boldsymbol{v}_i = \mathbf{0}, \boldsymbol{v}_j = \mathbf{0}\}$, and $\mathbb{Y}'$ be the set of compact extreme pathways of $\mathbb{V}'$, $\mathbb{Y}' = \{\mathbf{y}^1, \mathbf{y}^2, \cdots, \mathbf{y}^l\}$. Obviously, $\forall\, \mathbf{y} \in \mathbb{Y}', \boldsymbol{y}_i = \mathbf{0} \text{ and } \boldsymbol{y}_j = \mathbf{0}$. By Statement E, we have $\mathbb{Y}' \subseteq \mathbb{Y}$. If $\exists\, \mathbf{v}^0 \in \{\mathbf{v}|\mathbf{v} \in \mathbb{V}, \boldsymbol{v}_i = \mathbf{0}, \boldsymbol{v}_j = \mathbf{0}, \exists\, \boldsymbol{u} + \mathbf{1} \leq \boldsymbol{t} \leq \boldsymbol{u} + \boldsymbol{w}, \boldsymbol{v}_t \neq \mathbf{0}\}$, obviously $\mathbf{v}^0 \in \mathbb{V}'$, thus $\exists \boldsymbol{\alpha}_1, \boldsymbol{\alpha}_2, \ldots \boldsymbol{\alpha}_l \geq \mathbf{0}$, which satisfies the condition that $\mathbf{v}^0 = \sum_{k=1}^{l} \boldsymbol{\alpha}_k \boldsymbol{y}^k$. On the other hand, $\exists\, \boldsymbol{u} + \mathbf{1} \leq \boldsymbol{t} \leq \boldsymbol{u} + \boldsymbol{w}, \boldsymbol{v}_t^0 \neq \mathbf{0}$, so $\exists\, \mathbf{1} \leq \boldsymbol{k} \leq \boldsymbol{l}, \boldsymbol{y}_t^k \neq \mathbf{0}$, meaning that $\mathbf{y}^k$ is not an extreme pathway of type III. In sum, if $\exists\, \mathbf{v}^0 \in \{\mathbf{v}|\mathbf{v} \in \mathbb{V}, \boldsymbol{v}_i = \mathbf{0}, \boldsymbol{v}_j = \mathbf{0}, \exists\, \boldsymbol{u} + \mathbf{1} \leq \boldsymbol{t} \leq \boldsymbol{u} + \boldsymbol{w}, \boldsymbol{v}_t \neq \mathbf{0}\}$, then $\exists\, \mathbf{y} \in \mathbb{Y}$, which is an extreme pathway of type I or II and $\boldsymbol{y}_i = \mathbf{0}, \boldsymbol{y}_j = \mathbf{0}$. This contradicts the previous assumption; therefore the statement is true. □

Intuitively, Statement F indicates that if the two reactions r_i and r_j in the same EqSet always participate in different extreme pathways, then deleting both of them will disrupt the entire

metabolic network, meaning that all the reactions will have fluxes of zero. However, it is well known that a metabolic system has high robustness, so the situation above is not likely to occur. Therefore, it is safe to conclude that an EqSet has a synchronous nature.

Calculating extreme pathways for target systems

The protocol for the extreme pathway calculation of a target subsystem is illustrated in Fig A. The process begins with the definition of a target subsystem. Here reactions v_1, v_2 and v_3 are assigned to the target subsystem and the others are assigned to the surrounding subsystem. The metabolites utilized by the reactions in both the target and the surrounding subsystems are each split into two. For example, metabolites A, B and C in Fig A(a) are split into A and A', B and B', C and C', respectively, where A, B and C are in the target subsystem while A', B' and C' are in the surrounding subsystem. A bidirectional reaction is added between the two split metabolites, which corresponds to $\mathrm{A} \leftrightarrow \mathrm{A}'$, $\mathrm{B} \leftrightarrow \mathrm{B}'$ and $\mathrm{C} \leftrightarrow \mathrm{C}'$**,** respectively, in Fig A(b) for A, B and C**.** It is proven in **Statement G** that adding such reactions with only one substrate and one product does not change the constraints of the flux distribution of steady states. Now the target subsystem connects the surroundings through only these newly added reactions. Then flux variability analysis (FVA) [2] is applied to calculate the flux scope of these connecting reactions, depending on which feasible directions are determined. For example, the flux scope of $\mathrm{A} \leftrightarrow \mathrm{A}'$ is zero to negative, so the feasible direction is $\mathrm{A} \leftarrow \mathrm{A}'$**;** the flux scope of $\mathrm{B} \leftrightarrow \mathrm{B}'$is negative to positive, so both directions are feasible; and the flux scope of $\mathrm{C} \leftrightarrow \mathrm{C}'$ is zero to positive, so the feasible direction is $\mathrm{C} \rightarrow \mathrm{C}'$ (Fig A(c))**.** The corresponding relation between the flux scope and the feasible direction is listed in Table 6 of the paper. The next step is to convert the connecting reactions to the exchange reaction of the target subsystem by removing the metabolite in the surrounding subsystem. In Fig A(d), the connecting reaction $\mathrm{A} \leftarrow \mathrm{A}'$ is converted to the exchange reaction $\mathrm{A} \leftarrow$, $\mathrm{B} \leftrightarrow \mathrm{B}'$ is converted to $\mathrm{B} \leftrightarrow$, and $\mathrm{C} \rightarrow \mathrm{C}'$ is converted to $\mathrm{C} \rightarrow$**.** Finally, the extreme pathways of the target subsystem are calculated. There are altogether 3 extreme pathways in the example target subsystem shown in Fig A(d).

Statement G. Let $\mathbb{V}$ be the space of all the feasible steady states of a metabolic network with m metabolites and n reactions, $\mathbb{V} = \{\mathbf{v}|\mathbf{S}\mathbf{v} = \mathbf{0} \text{ and } v_i^{\boldsymbol{min}} \le \boldsymbol{v_i} \le v_i^{\boldsymbol{max}}, i = 1,2,\dots,n\}$, where $\mathbf{S} = \begin{bmatrix} s_{11} & \cdots & s_{1n} \\ \vdots & \ddots & \vdots \\ s_{m1} & \cdots & s_{mn} \end{bmatrix}$. Adding reaction $\mathrm{M} \leftrightarrow \mathrm{M}'$ to connect the target subsystem and the surrounding system does not change the constraints of $v_i, i = 1,2,\dots,n$.

Proof. Without losing generality, we assume that the mth line of the stoichiometric matrix $\mathbf{S}$ corresponds to metabolite M and the first k columns of $\mathbf{S}$ correspond to the reaction of the target subsystem. The reaction $\mathrm{M} \leftrightarrow \mathrm{M}'$ is added to connect the target subsystem and the surrounding system by augmenting $\mathbf{S}$ to $\mathbf{S}'$ and $\mathbf{v}$ to $\mathbf{v}'$, respectively. Here, $\mathbf{S}'$ is a $m+1$ by $n+1$ matrix, $\mathbf{S}' = \begin{bmatrix} s_{11} & \cdots & s_{1k} & s_{1(k+1)} & \cdots & s_{1n} & 0 \\ \vdots & \ddots & \vdots & \vdots & \ddots & \vdots & \vdots \\ s_{m1} & \cdots & s_{mk} & 0 & \cdots & 0 & -1 \\ 0 & \cdots & 0 & s_{m(k+1)} & \cdots & s_{mn} & 1 \end{bmatrix}$, $s'_{i(n+1)} = 0$ for $i = 1,2,\dots,n-1$. The $(m+1)$th row of $\mathbf{S}'$ corresponds to the metabolite M', and the $(n+1)$th column of $\mathbf{S}'$ corresponds to

the reaction $\mathrm{M} \leftrightarrow \mathrm{M}'$. Thus, the space of feasible steady states of the augmented metabolic network is $\mathbb{V}' = \{\mathbf{v}|\mathbf{S}'\mathbf{v}' = \mathbf{0} \text{ and } v_i^{min} \leq \boldsymbol{v_i} \leq v_i^{max}, i = 1,2, \dots, n\}$.
$\mathbf{Sv} = \mathbf{0}$ can be equally written as $\sum_{j=1}^{n} s_{ij} v_j = 0, \text{for i} = 1, \dots, m$. Similarly, $\mathbf{S}'\mathbf{v}' = \mathbf{0}$ can be equally written as $\sum_{j=1}^{n+1} s'_{ij} v'_j = 0$, for $\mathrm{i} = 1, \dots, m+1$. When $i = 1,2, \dots, m-1$, we have $\sum_{j=1}^{n} s'_{ij} v'_j = 0$. When $i = m$, we have $\sum_{j=1}^{k} s_{mj} v_j - v'_{n+1} = 0$. When $i = m+1$, we have $\sum_{j=k+1}^{n} s_{mj} v_j + v'_{n+1} = 0$. Therefore, $\sum_{j=1}^{k} s_{mj} v_j - v'_{n+1} + \sum_{j=k+1}^{n} s_{mj} v_j + v'_{n+1} = \sum_{j=1}^{n} s_{mj} v_j = 0$. In other word, $\sum_{j=1}^{n} s_{ij} v_j = 0, \text{for i} = 1, \dots, m$, which is equivalent to $\mathbf{Sv} = \mathbf{0}$. Therefore, the constraints remain unchanged for reaction $v_i, i = 1,2, \dots, n$. □

A similar protocol for calculating the extreme pathways of subsystems in a metabolic network was developed by Schilling and Palsson[3]. The main idea of that protocol is to divide the metabolic network into several subsystems and then specify the exchange flux constraints for all common metabolites of each subsystem by iteratively calculating the extreme pathway of the subsystems and determining the production and consumption characteristics of each common metabolite from the extreme pathways in all the subsystems. The final set of extreme pathways is obtained when all the exchange flux constraints are unchangeable.

Compared to the protocol of Schilling and Palsson, our protocol has the following advantages. First, by subdividing a metabolic network into target and surrounding subsystems, the scheme of subdivision can be more versatile and flexible. For example, a reaction can be placed in several target subsystems that represent different functions of the metabolic system. Since some reactions are involved in several metabolic processes, such as those in the citric acid cycle that play important roles in both glycometabolism and lipometabolism, placing them in multiple target subsystems will be more biologically meaningful. Second, the extreme pathways calculated for the target subsystem by our approach are unique. However, those calculated for a subsystem based on Schilling and Palsson's protocol may vary with the subdivision of the rest of the metabolic network. This may be a bit confusing to researchers who use these extreme pathways to study the structural properties and metabolic capabilities of the subsystems. An example is given in Fig B. Last, but not least, our approach is more effective than Schilling and Palsson's. In Schilling and Palsson's protocol, the extreme pathway calculation is run for each subsystem for several iterations, which is time consuming. In contrast, our protocol calculates the extreme pathways only once. The cost for this is dozens or hundreds of times of linear optimization required in FVA, which is much faster than the extreme pathway calculation.

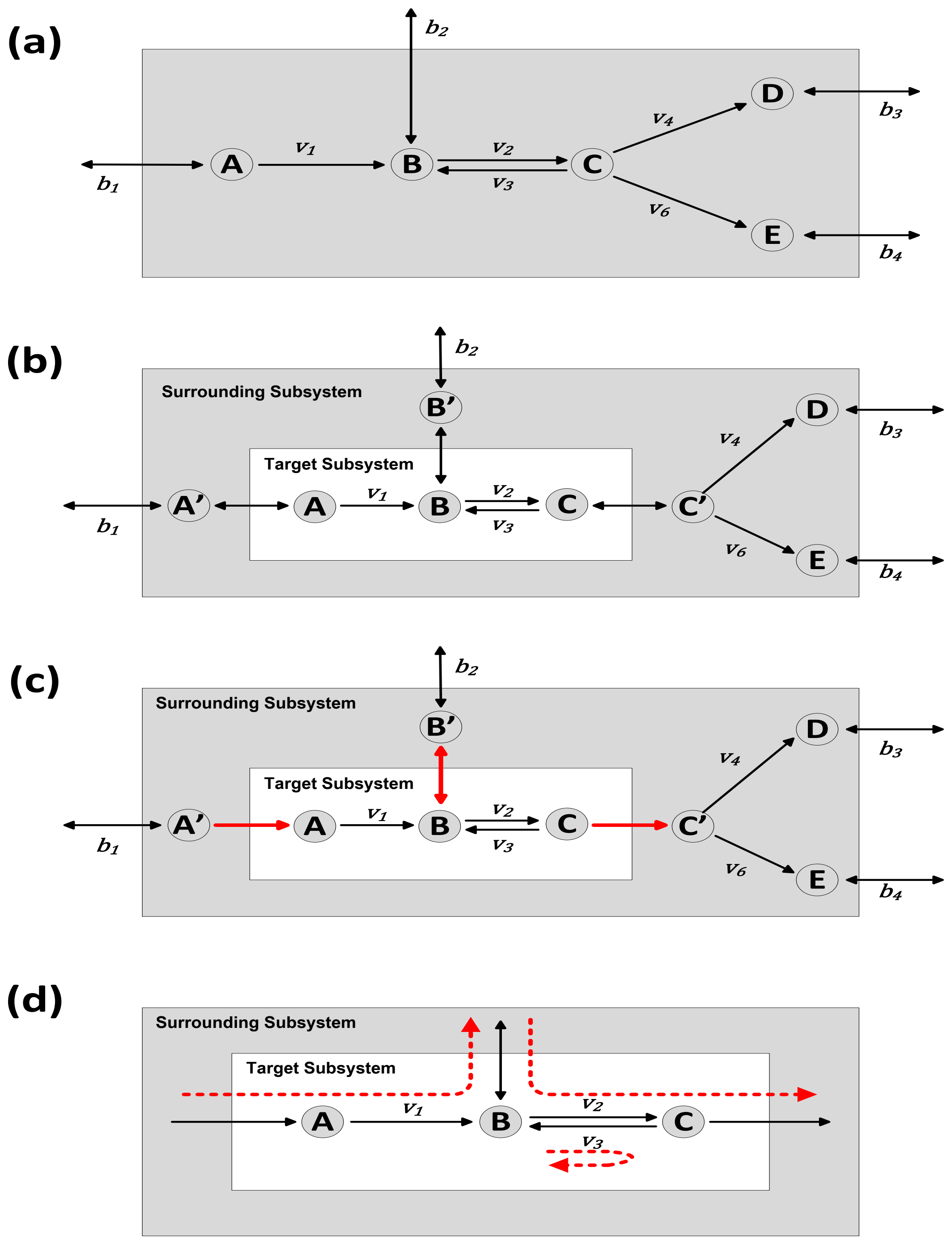

Fig A. Protocol for the extreme pathway calculation of a target subsystem.

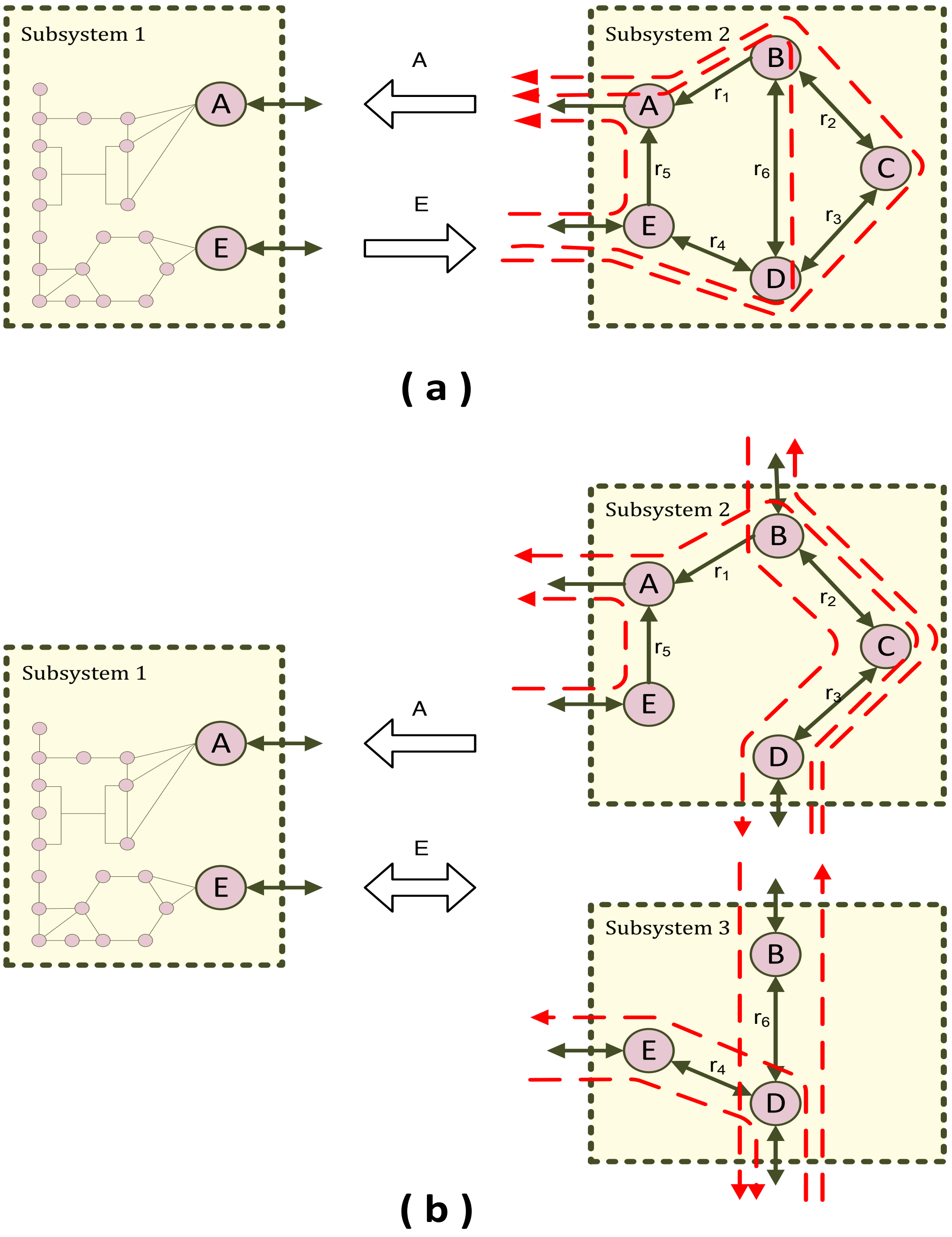

Fig B. An example shows that the extreme pathways calculated by Schilling and Palsson's protocol may vary with the subdivision of the rest of the network. Subsystem 1 has the potential to both produce and consume the metabolites A and E. The red and dotted lines with arrows present the extreme pathways of a subsystem, and the arrows indicate the flow direction of the extreme pathways. (a) If the reactions not belonging to subsystem 1 are placed in the same subsystem, the constraints of the exchange fluxes of A and E in subsystem 1 will be negative and positive, respectively. (b) However, if the reactions other than those in subsystem 1 are subdivided into two subsystems, as shown here, the constraints of the exchange fluxes of A and E in subsystem 1 will be negative and unconstrained. The difference in the constraints may result in different sets of extreme pathways.

The algorithm for sorting EqSets by their regulatory importance

Algorithm A shows how the EqSets are sorted. It takes six inputs and gives a sequence of the internal EqSets $\mathbb{Q}$, in which the EqSets ranked higher in the sequence are of higher regulatory importance. The inputs include $\widehat{\mathbf{Y}}$, $\mathbf{D}$, τ, ρ_e, ρ_s and μ, where $\widehat{\mathbf{Y}}$ is the matrix of binary compact extreme pathways; $\widehat{\mathrm{Y}}_{i,j} = \begin{cases} 0, & \text{if } y_i^j = 0 \\ 1, & \text{if } y_i^j \neq 0 \end{cases}$, $\mathbf{D}$ is the matrix of the regulatory distance between EqSets; the element $\mathrm{D}_{i,j} = D(\mathbb{X}_i, \mathbb{X}_j)$ is the regulatory distance between $\mathbb{X}_i$ and $\mathbb{X}_j$; τ is the size of the sliding window; ρ_e and ρ_s are the effective radii, of which the former designates the maximum regulatory distance from a reaction to its nearest counterpart in the same EqSet and the latter designates the maximum regulatory distance that an EqSet influences; and μ is a parameter controlling the strength when the regulatory importance of an EqSet is adjusted according to its size.

The algorithm begins with a pool $\mathbb{P}$ containing all the EqSets and an empty queue of predicted regulatory EqSets $\mathbb{Q}$. Then, it iteratively picks up the EqSet with the highest regulatory importance from $\mathbb{P}$ and moves it to the end of $\mathbb{Q}$. The iteration stops when $\mathbb{P}$ is empty or the regulatory importance of any EqSet remaining in $\mathbb{P}$ equals zero. At last, the algorithm removes the EqSets composed of exchange reactions from $\mathbb{Q}$. The regulatory importance h_i of the EqSet $\mathbb{X}_i$ is evaluated by $w_i\left(h_i^{(1)} + h_i^{(2)}\right)$, where $h_i^{(1)} = \mathrm{H}(\mathbb{X}_i | \cup_{\mathbb{X}\in\mathbb{N}_i} \mathbb{X})$, and $h_i^{(2)} = \min_{\mathbb{X}_u \in \mathbb{C}_i} (\mathrm{H}(\mathbb{X}_i | \cup_{\mathbb{X}\in\mathbb{N}_i} \mathbb{X} \cup \mathbb{X}_u))$, and $w_i = 1 + \mu(|\mathbb{X}_i| - 1)$. $\mathbb{N}_i$ is a sub-queue of $\mathbb{Q}$, $\mathbb{N}_i = \begin{cases} \{\mathbb{X}_{x_1}, \mathbb{X}_{x_2}, \cdots, \mathbb{X}_{x_t}\}, & if\ t \leq \tau \\ \{\mathbb{X}_{x_{t-\tau+1}}, \mathbb{X}_{x_{t-\tau+2}}, \cdots, \mathbb{X}_{x_t}\}, & if\ t > \tau \end{cases}$, $\forall \mathbb{X} \in \mathbb{N}_i$, we have $\mathrm{D}(\mathbb{X}, \mathbb{X}_i) \leq \rho_s$. And $\mathbb{C}_i$ is a set of EqSets such that $\mathbb{C}_i = \{\mathbb{X}_u | \mathbb{X}_u \notin \mathbb{Q}, \mathrm{u} \neq i \text{ and } \mathrm{D}(\mathbb{X}_u, \mathbb{X}_i) \leq \rho_s\}$.

Input: $\widehat{\mathbf{Y}} = [\hat{\mathbf{y}}^1, \hat{\mathbf{y}}^2, \cdots, \hat{\mathbf{y}}^l]$, $\mathbf{D}$, τ, ρ_e, ρ_s, μ
Output: $\mathbb{Q}$, a sequence of internal EqSets ordered by regulatory importance

/* Calculating Equivalent Reaction Sets on Entropy (EqSets) */
$\mathbb{R} := $ all reactions in metabolic network
$\mathbb{X} := \emptyset$ // EqSets
while $\mathbb{R} \neq \emptyset$
    Pick a reaction r_i from $\mathbb{R}$, let $\mathbb{X}_i := \{\mathrm{r}_i\}$
    while $\exists \mathrm{r}_j \in \mathbb{X}_i, \mathrm{r}_k \in \mathbb{R} - \mathbb{X}_i, \mathrm{s.t.}, \mathrm{H}(\mathrm{r}_j|\mathrm{r}_k) = \mathrm{H}(\mathrm{r}_k|\mathrm{r}_j) = 0, d_{j,k} \le \rho_e$
        $\mathbb{X}_i = \mathbb{X}_i \cup \{\mathrm{r}_k\}$ // an EqSet
    end
    $\mathbb{R} = \mathbb{R} - \mathbb{X}_i$
    $\mathbb{X} = \mathbb{X} \cup \{\mathbb{X}_i\}$
end

/* Sorting EqSets by their regulatory importance*/
$\mathbb{P} := \mathbb{X}$
$\mathbb{Q} := []$
while $\mathbb{R}^{\mathrm{cad}} \neq \emptyset$
    for each reaction $\mathbb{X}_i \in \mathbb{P}$
        $\mathbb{R}_i^{\mathrm{ngh}} = \{\mathbb{X}_j | \mathbb{X}_j \in \mathbb{Q}, D(\mathbb{X}_i, \mathbb{X}_j) \le \rho_s\}$
        $\mathbb{R}_i^{\mathrm{win}} := \{\text{The latest } \tau \text{ EqSets added into } \mathbb{R}_i^{\mathrm{ngh}}\}$
        $\mathbb{R}_i^{\mathrm{cmp}} = \{\mathbb{X}_j | \mathbb{X}_j \in \mathbb{P}, D(\mathbb{X}_i, \mathbb{X}_j) \le \rho_s\}$
        $w_i = 1 + \mu(|\mathbb{X}_i| - 1)$
        $h_i = w_i\left(h_i^{(1)} + h_i^{(2)}\right)$
    end
    if $\forall \mathbb{X}_k \in \mathbb{P}$ that $h_k = 0$
        break;
    end
    Pick an EqSet $\mathbb{X}_k$ such that $\forall \mathbb{X}_j \in \mathbb{P}, h_j \le h_k$
    $\mathbb{Q} = \mathbb{Q} \cup \{\mathbb{X}_k\}$; $\mathbb{P} = \mathbb{P} - \{\mathbb{X}_k\}$
end

Remove the EqSets composed of exchange reactions from $\mathbb{Q}$

Algorithm A. Sorting EqSets by their regulatory importance

Evaluation of the internal EqSet sequence and calculation of the corresponding p-value

A biologically meaningful EqSet is defined as one that contains reactions that impact the organism, i.e., reactions related to regulatory functions or disease processes. Algorithm A aims to sort the internal EqSets by their regulatory importance or relationship with disease (for the human metabolic network), so the lower the rank of the known biologically meaningful EqSets, the more meaningful the resulting sequence is in biology. On the other hand, as there are quite a few biologically meaningful EqSets in the sequence and their relative importance is unclear, we assume that swapping any two of them in the sequence will not affect the biological impact of the sequence. Thus, we evaluated the resulting sequence of Algorithm A by an evaluation score σ,

$$\sigma = \sum_{\substack{i=1,\cdots,s \\ \mathbb{X}_i \cap \mathbb{R}^{bm} \neq \emptyset}} \mathrm{rank}(\mathbb{X}_i) + d(s + \frac{l+1}{2})$$

where $\mathbb{Q}$ is a sequence of internal EqSets, $\mathbb{Q} = [\mathbb{X}_1, \mathbb{X}_2, \cdots \mathbb{X}_s]$, $\mathbb{R}^{bm}$ is the set of biologically meaningful reactions, i.e. regulatory or disease-related, reactions, $\mathrm{rank}(\mathbb{X}_i)$ is the rank of EqSet $\mathbb{X}_i$ in $\mathbb{Q}$, l is the number of the lost internal EqSets, and d is the number of the lost but biologically meaningful EqSets. This marking scheme assumes that the lost EqSets are placed at the end of sequence $\mathbb{Q}$ in a random order, which corresponds with the fact that we consider the EqSets absent from $\mathbb{Q}$ to be least important for regulation. From the next statement, we can easily deduce that $d(s + \frac{l+1}{2})$ equals the expectation of the rank summation of the lost EqSets that are biologically meaningful.

Statement H. Let $\mathbf{p}$ be a random permutation of $\boldsymbol{s}$ internal EqSets, $\mathbb{X}_1, \mathbb{X}_2, \cdots, \mathbb{X}_s$, $\boldsymbol{c}$ of which are biologically meaningful, and $\mathbf{E}(\boldsymbol{\sigma})$ be the expectation of the evaluation score for $\mathbf{p}$. Then,

$$\mathrm{E}(\sigma) = \frac{c}{s} \sum_{i=1}^{s} i = c(s+1)/2$$

Proof. Without loss of generality, we assume that the biologically meaningful EqSets are $\mathbb{X}_1, \mathbb{X}_2, \cdots, \mathbb{X}_c$. Let $\sigma_{\mathbf{p}}$ be the summation of the ranks of $\mathbb{X}_1, \mathbb{X}_2, \cdots, \mathbb{X}_c$ on $\mathbf{p}$. Since there is no EqSet absent from $\mathbf{p}$, d and l defined above equal 0 and $\sigma_{\mathbf{p}} = \sum_{i=1}^{c} \mathrm{rank}_{\mathbf{p}}(\mathbb{X}_i)$. Therefore, we have

$$
\begin{aligned}
\mathrm{E}(\sigma) &= \frac{\sum_{\mathbf{p}} \sigma_{\mathbf{p}}}{s!} \\
&= \frac{\sum_{\mathbf{p}} \sum_{i=1}^{c} \mathrm{rank}_{\mathbf{p}}(\mathbb{X}_i)}{s!} \\
&= \frac{\sum_{i=1}^{c} \sum_{\mathbf{p}} \mathrm{rank}_{\mathbf{p}}(\mathbb{X}_i)}{s!} \\
&= \frac{\sum_{\mathbf{p}} \mathrm{rank}_{\mathbf{p}}(\mathbb{X}_1) + \sum_{\mathbf{p}} \mathrm{rank}_{\mathbf{p}}(\mathbb{X}_2) + \ldots + \sum_{\mathbf{p}} \mathrm{rank}_{\mathbf{p}}(\mathbb{X}_c)}{s!}
\end{aligned}
$$

where $\mathrm{rank}_{\mathbf{p}}(\mathbb{X}_i)$ denotes the rank of $\mathbb{X}_i$ in $\mathbf{p}$. $\forall i = 1, \cdots, c$, $\sum_{\mathbf{p}} \mathrm{rank}_{\mathbf{p}}(\mathbb{X}_i)$ can also be represented as $\sum_{k=1}^{s} k \times \left|\left\{\{\mathbf{p} | \mathrm{rank}_{\mathbf{p}}(\mathbb{X}_i) = k\right\}\right|$, where $\left|\left\{\{\mathbf{p} | \mathrm{rank}_{\mathbf{p}}(\mathbb{X}_i) = k\right\}\right|$ denotes the number of permutations satisfying the condition that $\mathbb{X}_i$ is placed on the kth position. Since the set $\{\mathbf{p} | \mathrm{rank}_{\mathbf{p}}(\mathbb{X}_i) = k\}$ is composed of the permutations in which $\mathbb{X}_i$ is in the kth position and the remaining $s-1$ EqSets are randomly arranged, we have $\left|\{\mathbf{p} | \mathrm{rank}_{\mathbf{p}}(\mathbb{X}_i) = k\right\}| = (s-1)!$. Therefore, $\sum_{\mathbf{p}} \mathrm{rank}_{\mathbf{p}}(\mathbb{X}_i) = \sum_{k=1}^{s} k\,(s-1)! = (s+1)!/2$

and $\mathrm{E}(\sigma) = \frac{\sum_{i=1}^{c}(s+1)!/2}{s!} = c(s+1)/2$. □

The p-value of sequence $\mathbb{Q}$ of the internal EqSets, whose evaluation score equals σ_0, is defined as the probability that a randomly arranged sequence of the same internal EqSets has an evaluation score that is no higher than σ_0.

Statement I. Suppose $\mathbf{p}$ is a random permutation of the internal EqSets $\mathbb{X}_1, \mathbb{X}_2, \ldots, \mathbb{X}_s$, in which $\mathbb{X}_1, \mathbb{X}_2, \ldots, \mathbb{X}_c$ are biologically meaningful, and let $\boldsymbol{p}(\boldsymbol{\sigma_0})$ be the probability that the evaluation score of $\mathbf{p}$ equals $\boldsymbol{\sigma_0}$, then we have

$$
\boldsymbol{p}(\boldsymbol{\sigma_0}) = \frac{\boldsymbol{N}(\boldsymbol{s}, \boldsymbol{c}, \boldsymbol{\sigma_0})}{\binom{\boldsymbol{s}}{\boldsymbol{c}}}
$$

where $\boldsymbol{N}(\boldsymbol{s}, \boldsymbol{c}, \boldsymbol{\sigma_0})$ is the number of the subsets of $\{\mathbf{1}, \mathbf{2}, \ldots, \boldsymbol{s}\}$ whose size equals c and the summation whose members add up to $\boldsymbol{\sigma_0}$.

Proof. Let $P(s, c, \sigma_0)$ be the number of permutations whose evaluation scores are σ_0, then obviously

$$
p(\sigma) = \frac{P(s, c, \sigma_0)}{s!} .
$$

The evaluation score of $\mathbf{p}$ is determined by the ranks of the internal EqSets $\mathbb{X}_1, \mathbb{X}_2, \ldots, \mathbb{X}_c$, denoted as $p_1, p_2, \ldots, p_c$. Furthermore, the permutations obtained by swapping among $\mathbb{X}_1, \mathbb{X}_2, \ldots, \mathbb{X}_c$ or among $\mathbb{X}_{c+1}, \mathbb{X}_{c+2}, \ldots, \mathbb{X}_s$ from $\mathbf{p}$ also have the evaluation score of σ_0. Therefore, we have $P(s, c, \sigma_0) = c!\,(s-c)!\,N(s, c, \sigma_0)$, where $c!$ equals the number of the permutations of $\mathbb{X}_1, \mathbb{X}_2, \ldots, \mathbb{X}_c$ and $(s-c)!$ equals the number of the permutations of $\mathbb{X}_{c+1}, \mathbb{X}_{c+2}, \ldots, \mathbb{X}_s$. In sum, the probability $p(\sigma)$ is defined as

$$p(\sigma) = \frac{P(s,c,\sigma_0)}{s!} = \frac{N(s,c,\sigma_0)}{\frac{s!}{c!\,(s-c)!}} = \frac{N(s,c,\sigma_0)}{\binom{s}{c}} \qquad \square$$

Suppose there are altogether s internal EqSets in a given metabolic network, in which c are biologically meaningful, and $\mathbb{Q}$ is a sequence of the internal EqSets whose evaluation score is σ_0, then the corresponding *p*-value, denoted as P, is

$$\mathrm{P} = \sum_{\sigma \le \sigma_0} p(\sigma_0) = \frac{1}{\binom{s}{c}} \sum_{\sigma \le \sigma_0} N(s,c,\sigma_0)$$

According to the definition of $N(s,c,\sigma_0)$, the constraints of s, c and σ_0 are as follows:

$$s \ge c \ge 1\,, m^{(c,s)} \le \sigma_0 \le M^{(c,s)},$$

where $m^{(c,s)} = c(c+1)/2$ and $M^{(c,s)} = c(2s-c+1)/2$, corresponding to the summation of the subsets $\{1,2,\ldots,c\}$ and $\{s-c+1, s-c+2,\ldots,s\}$, respectively. If $c=1$, then $N(s,c,\sigma_0)=1$. If $c>1$, the subsets of $\{1,2,\ldots,s\}$, whose members add up to σ_0, can be divided into two classes: those containing s and those not containing s. Therefore, the former class has $N(s-1,c-1,\sigma_0-s)$ substrates and the latter has $N(k-1,c,\sigma_0)$. In sum, for any positive integers s, c and σ_0, $N(s,c,\sigma_0)$ can be recursively defined as

$$N(s,c,\sigma_0) = \begin{cases} 0 & s < c, \text{or } \sigma_0 < m^{(c,s)}, \text{or } \sigma_0 > M^{(c,s)} \\ 1 & s \ge c, m^{(c,s)} \le \sigma_0 \le M^{(c,s)}, c = 1 \\ N(s-1,c-1,\sigma_0-s) + N(s-1,c,\sigma_0) & s \ge c, m^{(c,s)} \le \sigma_0 \le M^{(c,s)}, c > 1 \end{cases}$$

Thus, the value of $N(s,c,\sigma_0)$ can be calculated by dynamic programming, as shown in Algorithm B. In Algorithm B, the 3-dimension table $\mathbf{T}$ has $s \times c \times \sigma_0$ elements, each of which can be filled in $\mathrm{O}(1)$ times. Therefore, the algorithm runs in $\mathrm{O}(s \times \mathrm{c} \times \sigma_0)$ as the worst-case time. Since commonly $c \le s$ and $\sigma \le M^{(c,s)} = c(2s-c+1)/2$, we can restate the running time as $\mathrm{O}(s^4)$. Note that the algorithm is not polynomial, because the inputs are composed of three positive integers that can be encoded only by $\mathrm{O}(\log(s))$ bits, meaning that the instance of the problem it solves is of size $\mathrm{O}(\log(s))$ rather than $\mathrm{O}(\mathrm{s})$.

```
Input: s, c, σ_0, which are positive integers.
Output: N(s, c, σ_0)

/* Dealing with the simple cases. */
if s < c or σ_0 < m^(c,s) or σ_0 > M^(c,s)  then
      N(s, c, σ_0) = 0
      return
end

/* Initialization. */
T := a 3 dimension table of size s × c × σ_0, initialized to all 0
for k  from 1 to s
      for σ  from 1 to k
            T(k, 1, σ) = 1        /* Cases that  c=1 */
      end
end

/* Filling table by recursive definition. */
for k from 1 to s
      for n from 2 to min(k, c)
            for σ from m^(n,k) to min(M^(n,k), σ_0)
                  T(k, n, σ ) = T(k − 1, n − 1, σ − k) + T(k − 1, n, σ )
            end
      end
end

N(s, c, σ_0) =  T(s, c, σ_0)
```

Algorithm B. Calculating $N(s, c, \sigma_0)$ by dynamic programming.

The generation of artificial metabolic pathways

Algorithm C shows how a set of artificial metabolic pathways is generated. The algorithm takes three parameters: $\mathbf{Y} = [\mathbf{y}^1, \cdots, \mathbf{y}^l]$ is a matrix of compact extreme pathways, p is the probability that an extreme pathway in $\mathbf{Y}$ contributes to an artificial pathway, and t is the number of times that an artificial pathway is generated. Each pathway in $\mathbf{Y}$ **is** picked for a subset $\mathbb{I}$ under the probability p. Then, an artificial pathway $\mathbf{a}$ is obtained by summing all the pathways in $\mathbb{I}$. Therefore, the extreme pathways in $\mathbb{I}$ are said to contribute to the artificial pathway $\mathbf{a}$. The process above is repeated t times, which results in a set of t artificial pathways. Then the redundant ones are removed from the set and the remaining ones are transformed to binary forms. At last, the binary compact extreme pathways are also added to this set.

The artificial metabolic pathways can be treated as perturbations of the extreme pathways. The larger p is, the more the extreme pathways will be expected to contribute to an artificial pathway, and therefore the more divergent the artificial one will be from a single extreme pathway. On the other hand, for a given p, the larger t is, the greater the number of unique artificial pathways that will be obtained; therefore, the higher the proportion of artificial pathways that will be in the resulting set. In sum, the larger p and t are, the more perturbations there will be, and vice versa.

Input: $\mathbf{Y} = [\mathbf{y}^1, \mathbf{y}^2, \cdots, \mathbf{y}^l]$, p, t.
/* $\mathbf{Y}$ is a matrix of compact ExPas;
p is the probability that $\mathbf{y}^i$ contributes to a certain artificial pathway, where $i = 1, \cdots, l$;
t is the number of times that the generation of a single artificial pathway repeats */

Output: $\widehat{\mathbf{A}}$.
/* $\widehat{\mathbf{A}}$ is a matrix of binary artificial pathways generated from $\mathbf{Y}$ */

/* Generating artificial pathways */
for $k := 1$ **to** t
$\boldsymbol{\omega} := [\omega_1, \omega_2, \cdots, \omega_l]^{\mathrm{T}}$, where ω_i is a random variable, such that $P(\omega_i = 1) = p$ and $P(\omega_i = 0) = 1 - p$.
$\mathbf{a}^k := \mathbf{Y} \times \boldsymbol{\omega}$ // $\mathbf{a}^k$ is an artificial pathway
end
$\mathbf{A} = [\mathbf{a}^1, \mathbf{a}^2, \ldots, \mathbf{a}^t]$
Remove duplicated $\mathbf{a}^i$s from $\mathbf{A}$

/* Transforming $\mathbf{A}$ into its binary form*/
for $k := 1$ **to** $|\mathbf{A}|$
/* Denote the kth pathway in $\mathbf{A}$ as $\mathbf{a}^k = [x_1, x_2, \ldots, x_n]$, where n is the length of $\mathbf{a}^k$. */
$\hat{\mathbf{a}}^k := [\hat{x}_1, \hat{x}_2, \cdots, \hat{x}_n]$, where $\hat{x}_i = \begin{cases} 0, & x_i = 0 \\ 1, & x_i \neq 0 \end{cases}, i = 1,2, \ldots, n$
end

/* combine artificial pathways with compact ExPas*/
$\widehat{\mathbf{A}} := [\hat{\mathbf{a}}^1, \hat{\mathbf{a}}^2, \ldots, \hat{\mathbf{a}}^q, \hat{\mathbf{y}}^1, \hat{\mathbf{y}}^2, \cdots, \hat{\mathbf{y}}^l]$, where $[\hat{\mathbf{y}}^1, \hat{\mathbf{y}}^2, \cdots, \hat{\mathbf{y}}^l]$ is the binary form of $[\mathbf{y}^1, \mathbf{y}^2, \cdots, \mathbf{y}^l]$.

Algorithm C. Generating a set of artificial metabolic pathways from compact extreme pathways. ExPas, extreme pathways.

Identifying the reactions of transcriptional regulation in the E.coli metabolic network

We used the metabolic network iJR904 as the model of *E.coli* metabolism, in which 873 internal reactions have logical expressions, referred to gene-protein-reaction (GPR) associations, that represent the related coding gene of a reaction or an enzyme. Four types of representative GPR associations are shown in Fig C: In type one, one coding gene relates to one internal reaction, such as the gene b0003 and the reaction *HSK*. In type two, one coding gene relates to multiple reactions, such as the gene b0347 and the reactions *3HCINNMH* and *3HPPPNH*. In type three, multiple coding genes relate to one internal reaction, which can be further classified into two subtypes. In one subtype, the transcription and translation product of any of the related coding genes can catalyze the reaction, such as the genes b2914 and b4090 and the reaction *RPI;* while in the other subtype, the transcription and translation products of all the related coding genes are required to work together to catalyze the reaction, such as the genes b0615, b0616, and b0617 and the reaction *CITL*. In the fourth type of GPR association, multiple genes relate to multiple reactions; thus, this type is virtually a combination of the three formal types, seen in the example of genes b0997, b0996, b1873, and b1872 and the reactions *TMAOR1e* and *TMAOR1e*.

We adopted the rules of transcriptional regulation in the *E.coli* model iMC1010v1[4] as the blueprint for identifying the regulatory genes since the two models, iJR904 and iMC1010v1, are almost identical in the depiction of *E.coli* metabolism[4]. The regulatory rules in iMC1010v1 are obtained from the literature and are represented by logical expressions. A regulatory gene is the one that encodes metabolic enzymes in iJR904 and expresses according to the regulatory rules in iMC1010v1. For example, the gene b0997 in Fig C encodes the enzyme *TorI* and expresses according to a regulatory rule $(TorR\ \mathbf{OR}\ (\mathbf{Not}\ NarL))$, so it is a regulatory gene. There are 481 genes identified as regulatory ones in the iJR904 model.

Finally, we combined the rules of GPR associations with the regulatory genes above to identify the regulatory reactions that are controlled by the genes' transcriptional regulation. Any internal metabolic reaction will be identified as a regulatory reaction if any gene participating in its GPR association is regulated in transcription. For example, all the reactions in Fig C are regulatory reactions since there is at least one regulatory gene participating in the GPR association. Table S4 lists the internal reactions in model iJR904 and whether they are regulatory reactions.

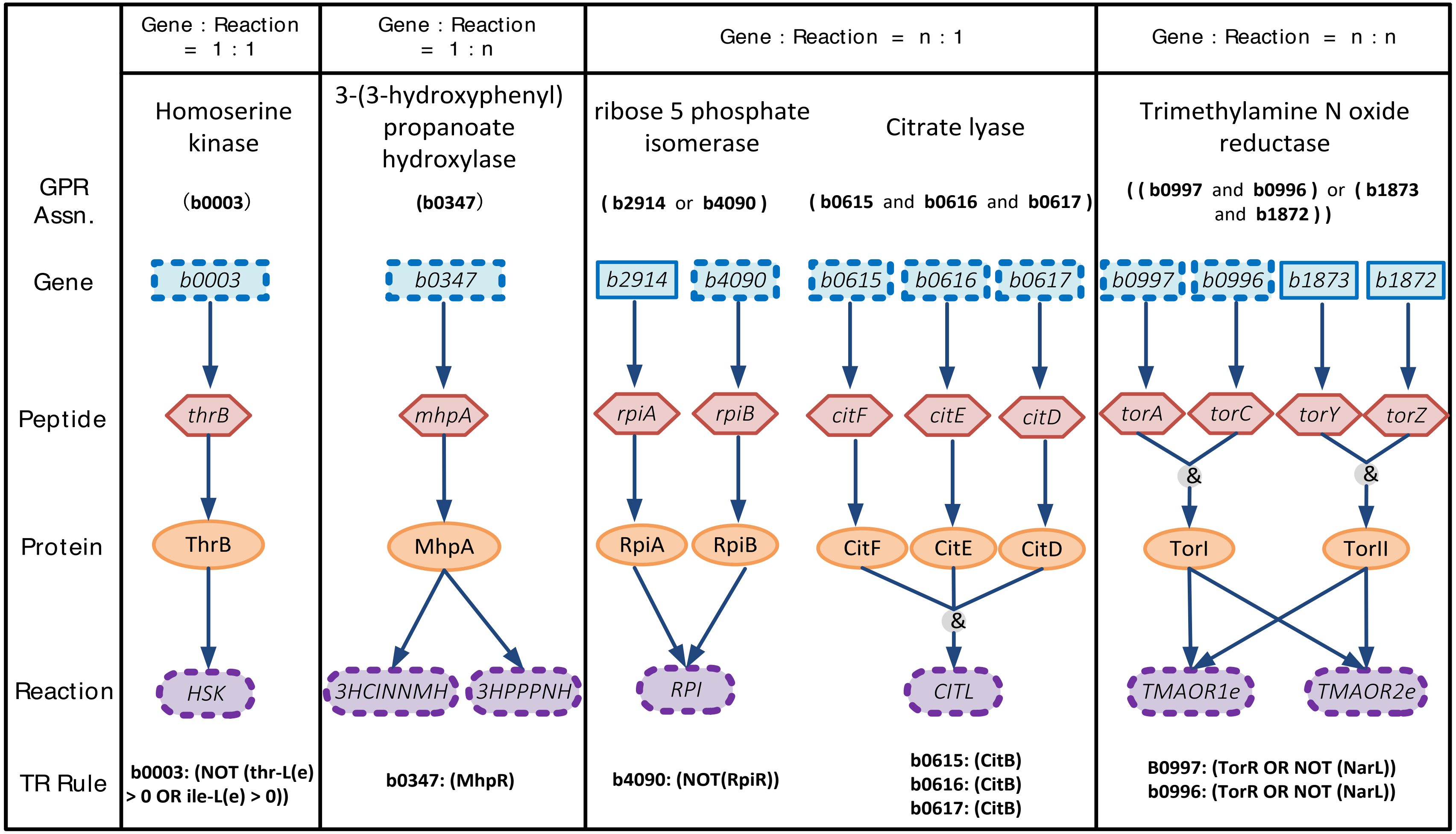

Fig C. Identifying the reactions of transcriptional regulation in the *E.coli* iRJ904 model from its gene-protein-reaction associations and transcriptional regulation rules. The blue, dashed rectangles denote the genes involved in transcriptional regulation; the eggplant, dashed ovals denote the regulatory reactions downstream. GPR Assn., gene-protein-reaction association, TR Rule, transcriptional regulation rule. In creating this figure, we followed the format established by Reed et al.[5], which appears under the Open Access policy of BioMed Central.

The relationship between degree of connection of reactions and metabolic regulation

A metabolic network can be transformed to a graph, in which a node represents an individual reaction and an edge indicates that the two reactions at the ends involve a common metabolite. An important topological property of the graph is the degree of each node [6], which is defined as the number of edges that connect with a specific node. The degree of reaction r_i is denoted as $\theta(r_i)$. The reactions that have high degrees are termed hub reactions and are regarded as important for analysis. We tried to determine whether there is a close relationship between the degree of connection in reactions and the distribution of regulatory points in a metabolic network.

First, we sorted the internal reactions of the human red blood cell (hRBC) metabolic network in ascending and descending order of their degree of connection, respectively, and calculated the evaluating score and the p-value of the resulting reaction sequence. For the reactions that had equal degrees of connection, we placed the known regulatory reactions in front of the others so as to ensure that the evaluation of the sequence was as good as possible. We carried out the same analysis for the *E.coli* metabolic network. The results are shown in Fig K(a) for the hRBC metabolic network and in Fig L for the three target subsystems of *E.coli*, in which we found no significant relation between the degree of connection and the distribution of regulatory reactions.

Second, we removed from the network the metabolites that participate in a large proportion of internal reactions, termed currency metabolites, as suggested in previous research[6]. Then, we recounted the degrees of connection, with which we sorted the reactions as stated above. We found the currency metabolites of the hRBC metabolic network to be H, H_2O, ATP, PI and ADP, which was relatively easy because the network is quite simple. In comparison, the metabolic network of *E.coli* contains thousands of reactions and metabolites and thus is much more complex. Clearly identifying the currency metabolites in even the subsystems of this metabolic network is challenging. Therefore, we sorted all the metabolites in the descending order of their participation rates, which are defined as the proportion of the reactions in which a metabolite participates. Then, we regarded the first k metabolites as currency metabolites, where k is a non-negative integer. Obviously, when more metabolites are removed from the network, the degree of connection will decline and become less diverse. The results are shown in Fig K(b) for the hRBC and in Fig M for the three target subsystems of *E.coli*. Again, it is difficult to recognize the regulatory reactions by only their degrees of connection.

A reasonable explanation for these results is that the regulatory architecture of a metabolic network is determined by the wide contact throughout the entire network rather than a local property, such as the degree of connection, which depicts only the interaction between a reaction and its neighbors.

Heuristic search for the optimal setting of the parameters

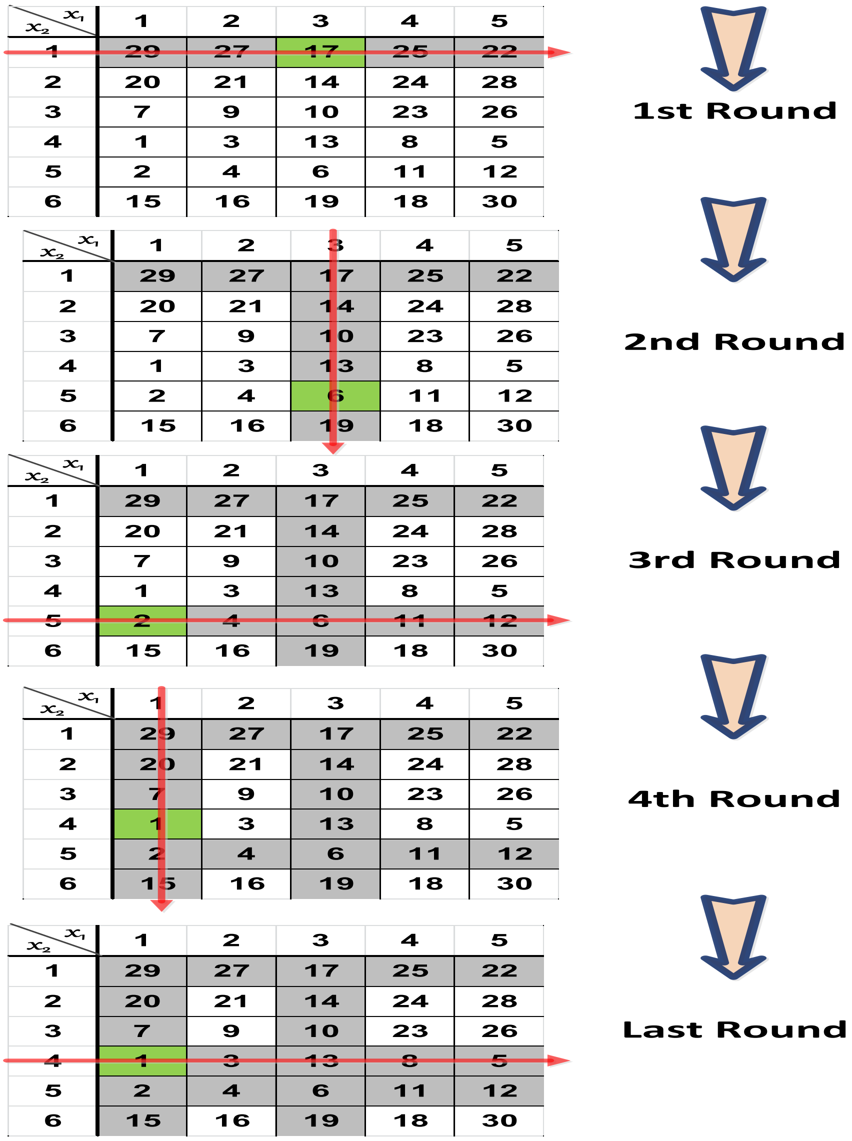

x_2 \ x_1	1	2	3	4	5
1	29	27	17	25	22
2	20	21	14	24	28
3	7	9	10	23	26
4	1	3	13	8	5
5	2	4	6	11	12
6	15	16	19	18	30

Fig D. The heuristic search algorithm for the optimum parameters on an example function. The function has two parameters, x_1 and x_2, which range from 1 to 5 and 6, respectively. The values relate to the parameters are listed in the tables. The algorithm aims to find the optimum parameter that corresponds to the lowest value of the function. It starts from $x_1 = \mathbf{2}$ and $x_2 = \mathbf{1}$**,** which are randomly selected. In round one, all the possible values of x_1 are tested for the function values when x_2 is fixed and x_1 is changed to the optimal value, 3. In the next round, all the possible values of x_2 are tested when x_1 equals 3 and x_2 is changed to the optimal value, 5. The next two rounds test and change x_1 and x_2 again, sequentially as stated above. Then x_1 and x_2 are set to 1 and 4, respectively. In the last round, x_1 is unchanged, which means a local optimum has been found for the parameters. Therefore, the algorithm stops. The shaded cells of the table denote the values of x_1 and x_2 which have been tested by the algorithm. The green cells indicate the lowest function value and the corresponding parameter values found in each round.

Supplementary Data

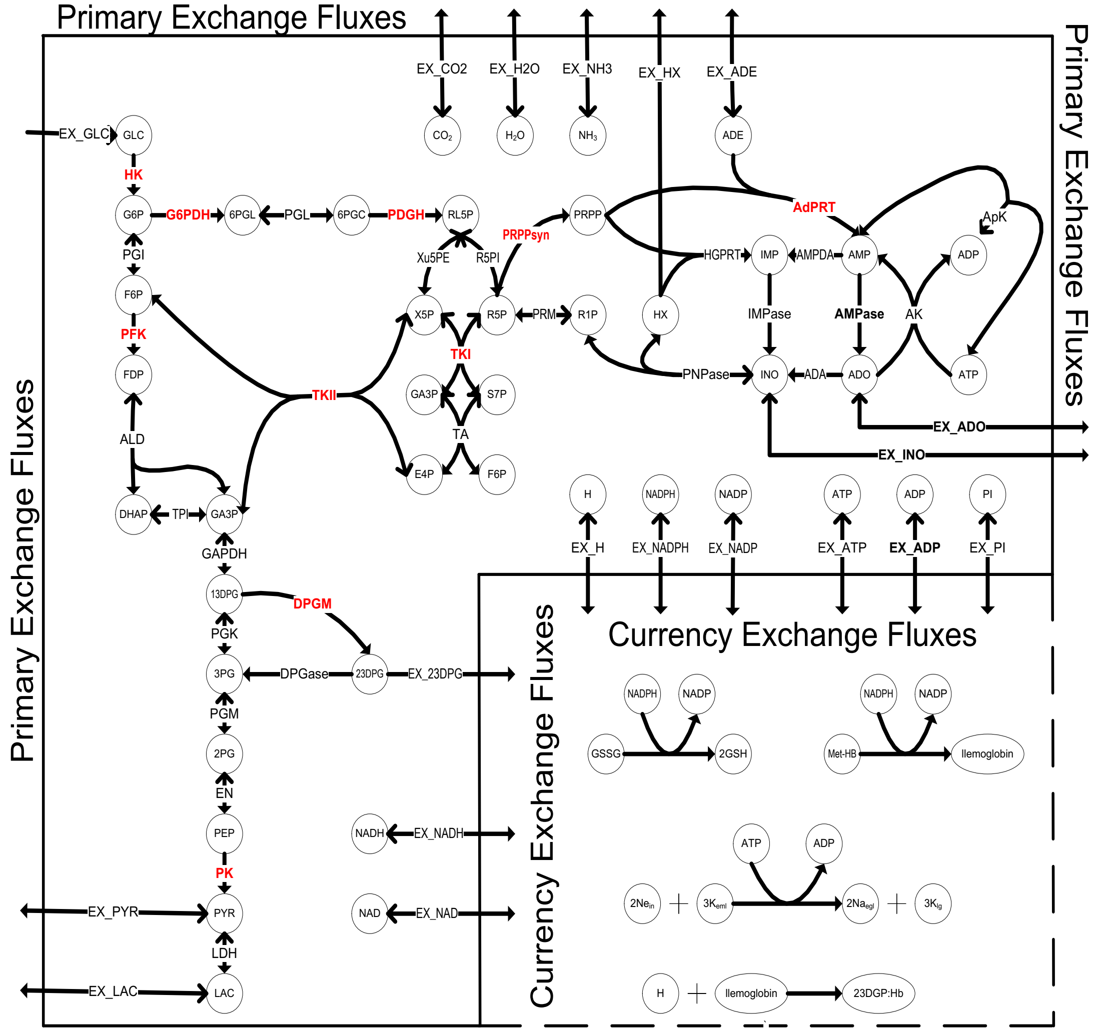

Fig E. A map depicting the metabolic network of a human red blood cell. The regulatory reactions are marked in red. This figure has been redrawn as a likeness of Figure 1 from the work of Wiback and Palsson[7].

Table A. Metabolites in the hRBC metabolic network [7]

Abbreviation	Full Name	Chemical formula
GLC	Glucose	C6 H12 O6
G6P	Glucose-6-phosphate	C6 H11 O9 P1
F6P	Fructose-6-phosphate	C6 H11 O9 P1
FDP	Fructose-1,6-phosphate	C6 H10 O12 P2
DHAP	Dihydroxyacetone phosphate	C3 H5 O6 P1
GA3P	Glyceraldehyde-3-phosphate	C3 H5 O6 P1
13DPG	1,3-Diphosphoglycerate	C3 H4 O10 P2
23DPG	2,3-Diphosphoglycerate	C3 H3 P2 O10
3PG	3-Phosphoglycerate	C3 H4 O7 P1
2PG	2-Phosphoglycerate	C3 H4 O7 P1
PEP	Phosphoenolpyruvate	C3 H2 O6 P1
PYR	Pyruvate	C3 H3 O3
LAC	Lactate	C3 H5 O3
6PGL	6-Phosphogluco-lactone	C6 H9 O9 P1
6PGC	6-Phosphogluconate	C6 H10 O10 P1
RL5P	Ribulose-5-phosphate	C5 H9 O8 P1
X5P	Xylulose-5-phosphate	C5 H9 O8 P1
R5P	Ribose-5-phosphate	C5 H9 O8 P1
S7P	Sedoheptulose-7-phosphate	C7 H13 O10 P1
E4P	Erythrose-4-phosphate	C4 H7 O7 P1
PRPP	5-Phosphoribosyl-1-pyrophosphate	C5 H8 O14 P3
IMP	Inosine monophosphate	C10 N4 H12 O8 P1
R1P	Ribose-1-phosphate	C5 H9 O8 P1
HX	Hypoxanthine	C5 N4 H4 O1
INO	Inosine	C10 H12 N4 O5
ADE	Adenine	C5 H5 N5
ADO	Adenosine	C10 H13 N5 O4
AMP	Adenosine monophosphate	C10 N5 H13 O7 P1
ADP	Adenosine diphosphate	C10 N5 H13 O10 P2
ATP	Adenosine triphosphate	C10 N5 H13 O13 P3
NAD	Nicotinamide adenine dinucleotide	NAD
NADH	Nicotinamide adenine dinucleotide(R)	NAD H1
NADP	Nicotinamide adenine dinucleotide phosphate	NADP
NADPH	Nicotinamide adenine dinucleotide phosphate(R)	NADP H1
H	Hydrogen ion	H1
Pi	Inorganic phosphate	H1O4P1
NH_3	Ammonia	N1 H3
CO_2	Carbon dioxide	C1 O2
H_2O	Water	H2 O1

Table B. Reactions in the hRBC metabolic network [7]

Abbreviation	Full Name	Chemical equation
HK	Hexokinase	GLU + ATP -> G6P + ADP + H
PGI	Phosphoglucoisomerase	G6P <=> F6P
PFK	Phosphofructokinase	F6P + ATP -> FDP + ADP + H
ALD	Aldolase	FDP <=> GA3P + DHAP
TPI	Triosephosphate isomerase	DHAP <=> GA3P
GAPDH	Glyceraldehyde phosphate dehydrogenase	GA3P + NAD + Pi <=> 13DPG + NADH + H
PGK	Phosphoglycerate kinase	13DPG + ADP <=> 3PG + ATP
DPGM	Diphosphoglyceromutase	13DPG -> 23DPG + H
DPGase	Diphosphoglycerate phosphatase	23DPG + H2O -> 3PG + Pi
PGM	Phosphoglyceromutase	3PG <=> 2PG
EN	Enolase	2PG <=> PEP + H2O
PK	Pyruvate kinase	PEP + ADP + H -> PYR + ATP
LD	Lactate dehydrogenase	PYR + NADH + H <=> LAC + NAD
G6PDH	Glucose-6-phosphate dehydrogenase	G6P + NADP -> 6PGL + NADPH + H
PGL	6-phosphoglyconolactonase	6PGL + H2O <=> 6PGC + H
PDGH	6-phosphoglycononate dehydrogenase	6PGC + NADP -> RL5P + NADPH + CO2
RPI	Ribose-5-phosphate isomerase	RL5P <=> R5P
XPI	Xylulose-5-phosphate epimerase	RL5P <=> X5P
TKI	Transketolase	X5P + R5P <=> S7P + GA3P
TA	Transaldolase	GA3P + S7P <=> E4P + F6P
TKII	Transketolase	X5P + E4P <=> F6P + GA3P
PRPPsyn	Phosphoribosyl pyrophosphate synthetase	R5P + ATP -> PRPP + AMP
PRM	Phosphoribomutase	R1P <=> R5P
HGPRT	Hypoxanthine guanine phosphoryl transferase	PRPP + HX + H2O -> IMP + 2Pi
AdPRT	Adenine phosphoribosyl transferase	PRPP + ADE + H2O -> AMP + 2Pi
PNPase	Purine nucleoside phosphorylase	INO + Pi <=> HX + R1P
IMPase	Inosine monophosphatase	IMP + H2O -> INO + Pi + H
AMPDA	Adenosine monophosphate deaminase	AMP + H2O -> IMP + NH3
AMPase	Adenosine monophosphate phosphohydrolase	AMP + H2O -> ADO + Pi + H
ADA	Adenosine deaminase	ADO + H2O -> INO + NH3
AK	Adenosine kinase	ADO + ATP -> ADP + AMP
ApK	Adenylate kinase	2 ADP <=> ATP + AMP

Continued.

Abbreviation	Full Name	Chemical equation
EX_GLC	Glucose exchange	GLC <-
EX_23DPG	2,3-Diphosphoglycerate exchange	23DPG ->
EX_PYR	Pyruvate exchange	PYR <=>
EX_LAC	Lactate exchange	LAC <=>
EX_HX	Hypoxanthine exchange	HX ->
EX_ADE	Adenine exchange	ADE <=>
EX_ADO	Adenosine exchange	ADO <=>
EX_INO	Inosine exchange	INO <=>
EX_ADP	Adenosine diphosphate exchange	ADP <=>
EX_ATP	Adenosine triphosphate exchange	ATP <=>
EX_NAD	Nicotinamide adenine dinucleotide exchange	NAD <=>
EX_NADH	Nicotinamide adenine dinucleotide (R) exchange	NADH <=>
EX_NADP	Nicotinamide adenine dinucleotide phosphate exchange	NADP <=>
EX_NADPH	Nicotinamide adenine dinucleotide phosphate (R) exchange	NADPH <=>
EX_PI	Inorganic phosphate exchange	PI <=>
EX_CO_2	Carbon dioxide exchange	CO2 <=>
EX_H	Hydrogen ion exchange	H <=>
EX_NH_3	Ammonia exchange	NH3 <=>
EX_H_2O	Water exchange	H2O <=>

Table C. Regulatory reactions in the hRBC metabolic network

Reaction	Regulatory mechanism	Reference
HK	HK (hexokinase) catalyzes the irreversible reaction: GLU + ATP -> G6P + ADP + H, which is the first step in glycolysis. HK is allosterically inhibited by its products, G6P and ADP. There are four important mammalian HK isozymes. The isozyme in human erythrocytes is HK I, which is so sensitive to inorganic phosphate that a small quantity of inorganic phosphate will release HK I from the inhibition of G6P. HK I is considered a “housekeeping enzyme,” and is unaffected by most physiological, hormonal, and metabolic changes.	[8, 9]
PFK	PFK (phosphofructokinase) is an allosteric enzyme that catalyzes the irreversible reaction: F6P + ATP -> FDP + ADP + H, which is the third step in glycolysis. PFK is the major rate-limiting enzyme in mammalian glycolysis. It is inhibited by ATP, while AMP releases the inhibition. When the pH declines, PFK will be inhibited by protons. It is an important mechanism of regulation that prevents the organism from experiencing acidosis by blocking glycolysis and stopping the production of lactate.	[8, 10, 11]
DPGM	DPGM (bisphosphoglycerate mutase) is an enzyme that is unique to erythrocytes and placental cells. It acts in glycolysis by catalyzing the reaction: 13DPG -> 23DPG + H. The product of this reaction, 23DPG, is a strong competitive inhibitor of DPGM.	[8]

PK	PK (pyruvate kinase) catalyzes the irreversible reaction: PEP + ADP + H -> PYR + ATP, which is the penultimate step in glycolysis. PK is an important allosteric regulatory enzyme in glycolysis that controls the production of PYR. Metabolites PEP and FDP enhance PK's enzymatic activity. Thus, glycolysis is driven to operate faster when more substrate is present. ATP is a negative allosteric inhibitor of PK, which slows glycolysis when the cell has enough energy. Alanine is also a negative allosteric modulator of PK.	8
G6PDH	G6PDH (glucose-6-phosphate dehydrogenase) catalyzes the irreversible reaction: G6P + NADP -> 6PGL + NADPH + H, which is the first step in the pentose phosphate pathway. G6PDH is the rate-limiting enzyme of the pentose phosphate pathway. G6PDH is stimulated by its substrate, G6P, and competitively inhibited by its product, NADPH.	8, 12, 13
PDGH	PDGH (6-phosphogluconate dehydrogenase) catalyzes the irreversible reaction: 6PGC + NADP -> RL5P + NADPH + CO2, which is the final step in the pentose phosphate pathway. PDGH is a regulatory enzyme of the pentose phosphate pathway. Similar to G6PDH, it is competitively inhibited by NADPH. On the other hand, it is stimulated by its substrate, 6PGC, in the absence of NADPH such that 6PGC can be metabolized quickly.	8, 14
TK	TK (transketolase) catalyzes two reversible reactions: X5P + R5P <=> S7P + GA3P (denoted as TKI) and X5P + E4P <=> F6P + GA3P (denoted as TKII), which connect the glycolytic and pentose phosphate pathways. TK is inhibited by inorganic phosphate and stimulated by both thiamine and ethanol. It balances the fluxes through the glycolytic and pentose phosphate pathways to respond to a cell's varying demand on the ratio of NADP and ATP produced.	8, 15, 16
AdPRT	AdPRT (adenine phosphoribosyltransferase) catalyzes the reversible reaction: PRPP + ADE + H2O -> AMP + 2Pi, which is involved in the purine nucleotide salvage pathway. The product of this reaction, AMP, is the competitive inhibitor of AdPRT with respect to PRPP.	8, 17, 18
PRPPsyn	PRPPsyn (phosphoribosyl pyrophosphate synthetase) catalyzes the reaction: R5P + ATP -> PRPP + AMP, which is the first step as well as the control site for adenosine nucleotide synthesis. PRPPsyn is allosterically regulated by ADP and GDP.	8

Supplementary Results

Table D. The entropy of each internal reaction in the hRBC metabolic network. The regulatory reactions are marked in red and underlined.

Rank	Reactions	Entropy ($\mathbf{H}(\mathbb{X})$)
**1**	**HK**	**0.9957**
2	GAPDH	0.9957
**3**	Xu5PE, **TKI**, **TKII**, TA	**0.9957**
**4**	**PFK**, ALD, TPI	**0.9612**
**5**	**G6PDH**, PGL, **PDGH**	**0.9612**
6	R5PI	0.9612
7	PNPase, PRM	0.9612
**8**	**DPGM**	**0.9183**
9	PGI	0.8905
**10**	PGM, EN, **PK**	**0.8905**
**11**	**PRPPsyn**	**0.8905**
**12**	**AdPRT**	**0.7793**
13	IMPase	0.7321
14	PGK	0.6790
15	DPGase	0.6790
16	ApK	0.6790
17	AMPase	0.6194
18	AMPDA	0.6194
19	AK	0.3912
20	HGPRT	0.3912
21	LDH	0.2918
22	ADA	0.1720

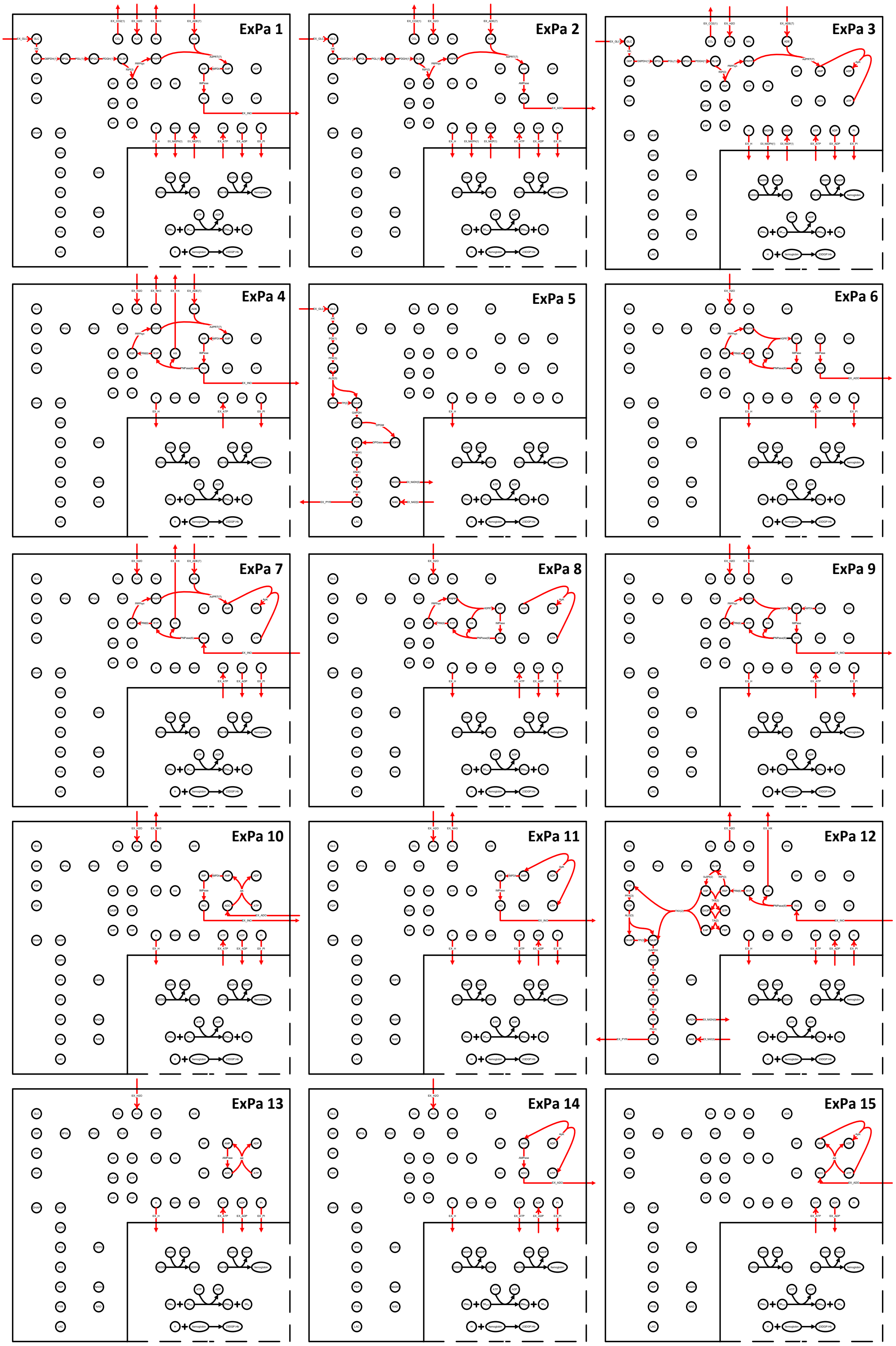

Fig F. Types I and II extreme pathways of the hRBC metabolic network (No.1-15)

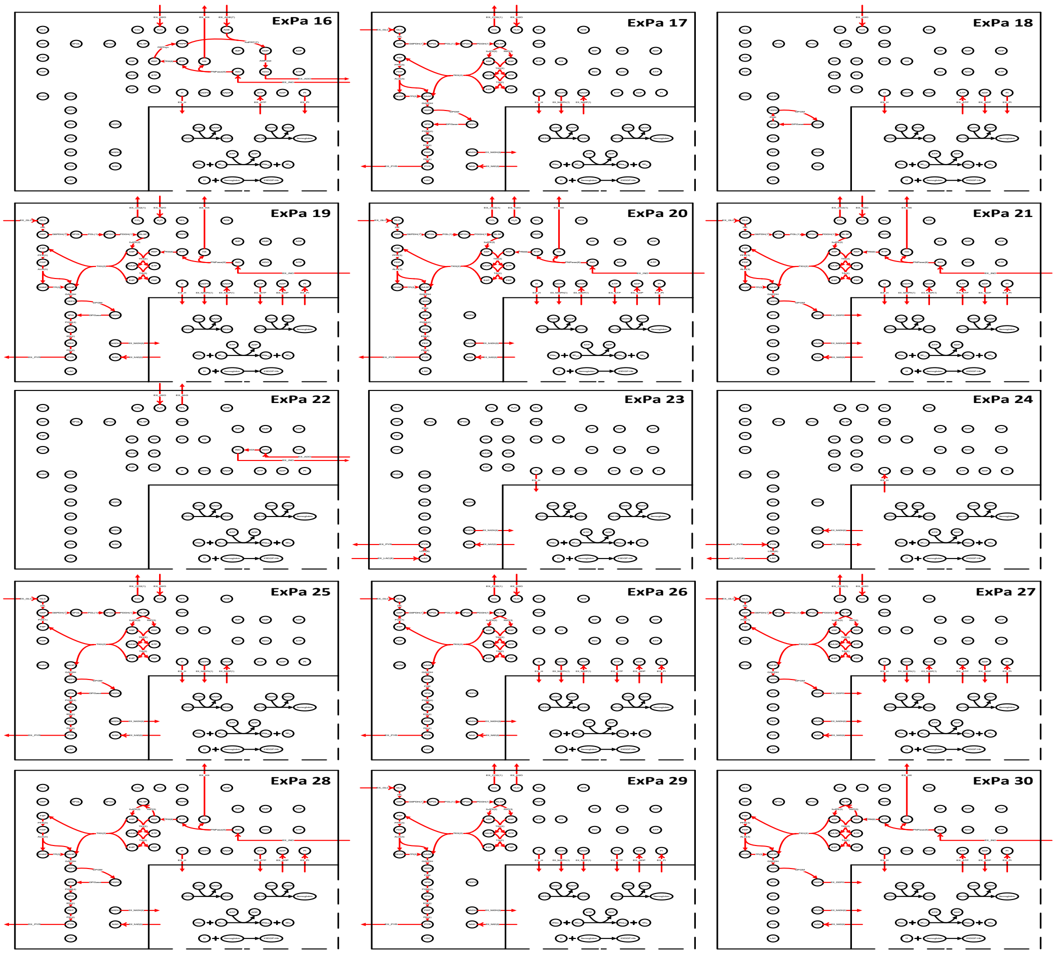

Fig G. Types I and II extreme pathways of the hRBC metabolic network (No.16-30)

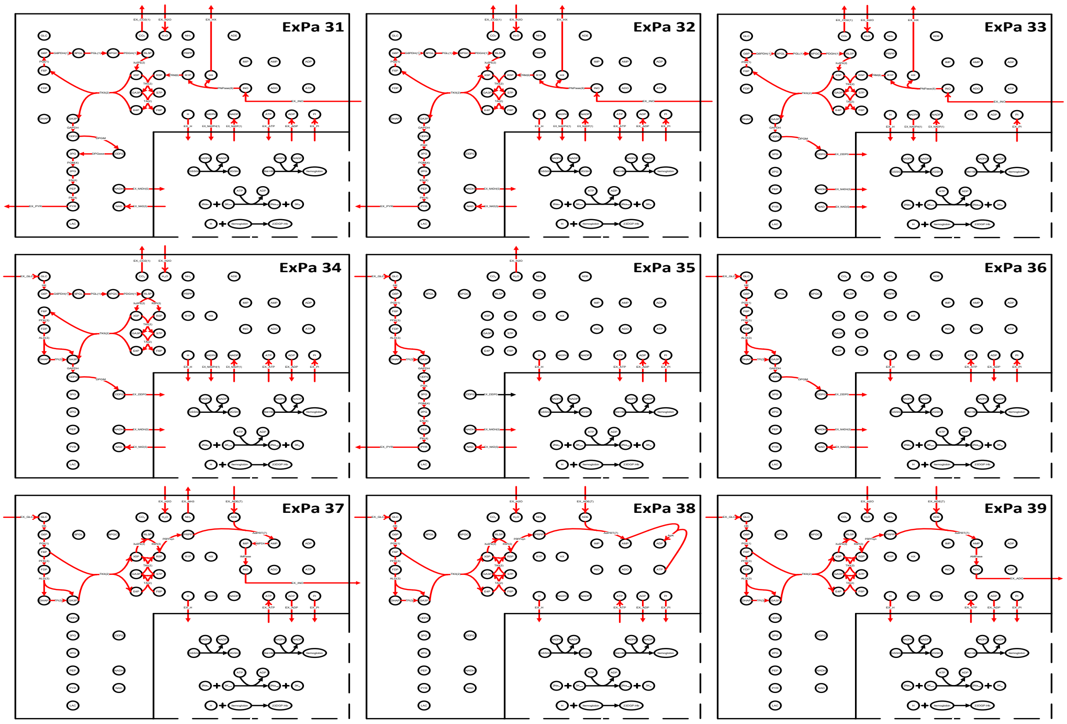

Fig H. Types I and II extreme pathways of the hRBC metabolic network (No.30-39)

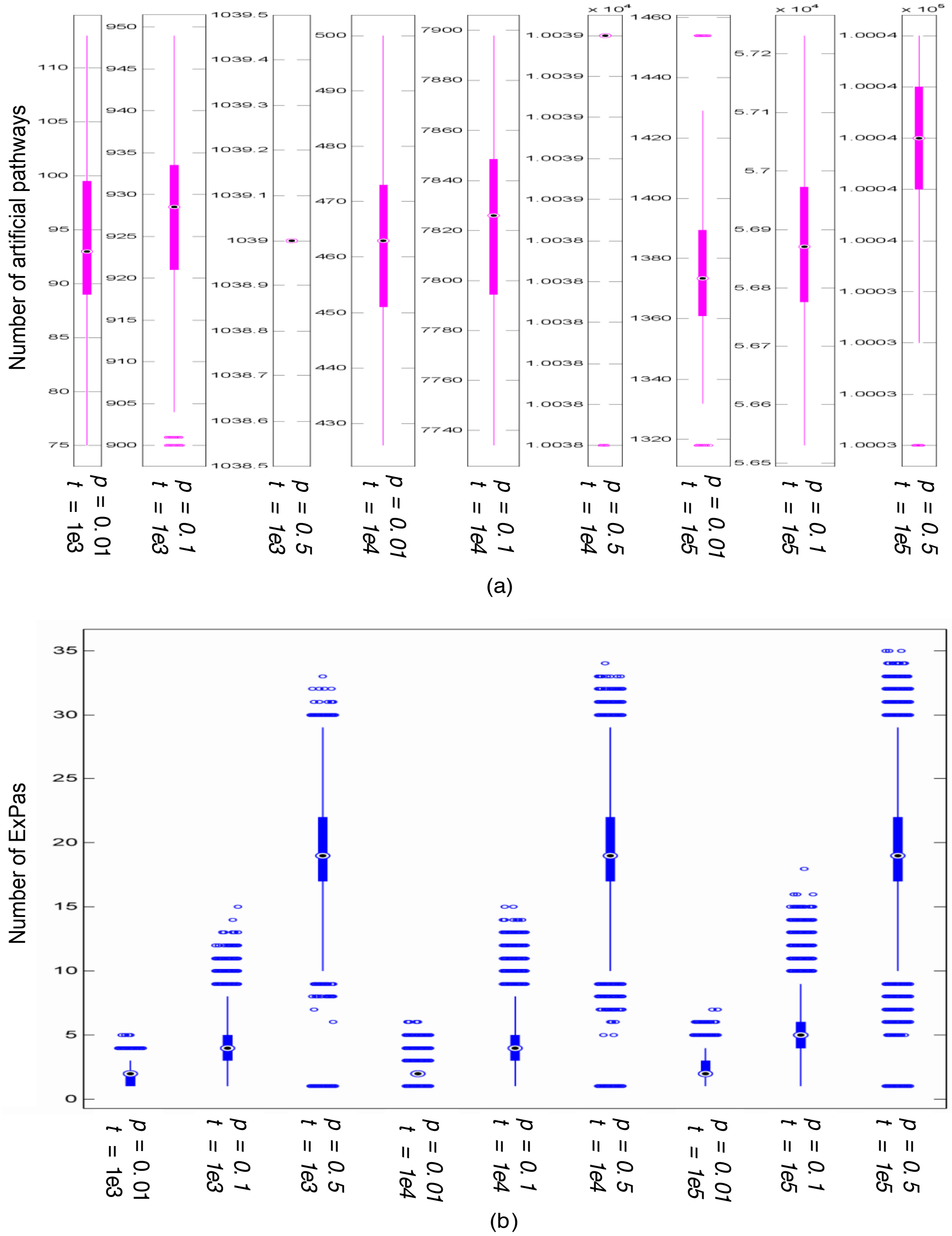

Fig I. Perturbation of the artificial metabolic pathways. The parameter p takes the value 0.01, 0.1, or 0.5 and t takes the value 1000, 10000, or 100000, the combination of which leads to 9 parameter tuples of different values. For each tuple, we ran **Algorithm C** 100 times and obtained 100 sets of randomly generated artificial metabolic pathways. (a) Boxplots show the distributions of size of the artificial pathway set. (b) Boxplots show the distribution of the numbers of compact extreme pathways contributing to an artificial pathway. The values of p and t are marked under each boxplot. It is clear that the size of the artificial pathway set and the number of compact extreme pathways contributing to an artificial pathway rise with p and t. Therefore, we can conclude that the higher the values of p and t, the more divergent the artificial pathway set will be from that of the compact extreme pathways, meaning that the perturbation brought by the artificial metabolic pathways will be stronger.

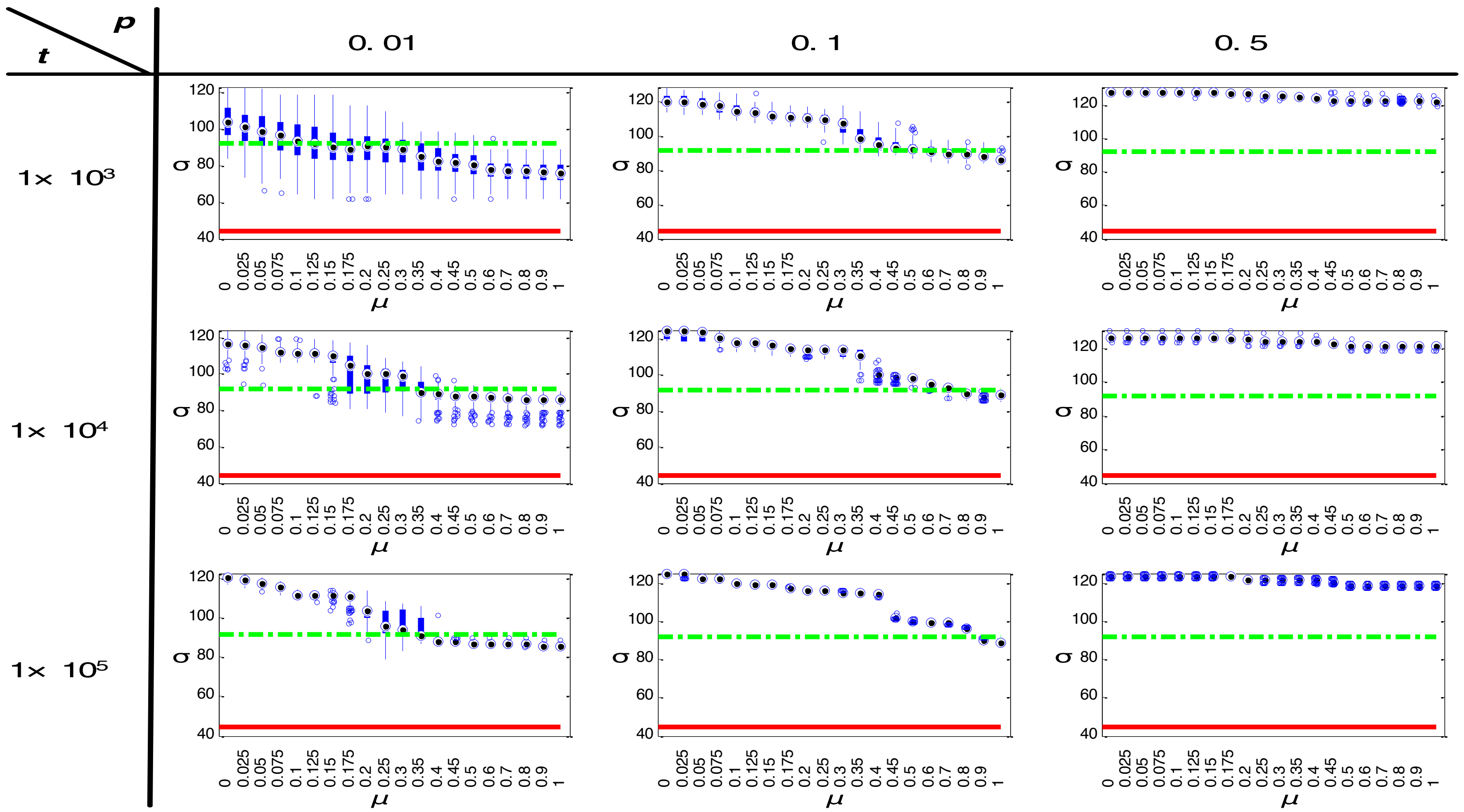

Fig J. Evaluation score distribution of the EqSet sequences calculated from artificial metabolic pathways. A set of artificial metabolic pathways are built as follows: (1) An artificial pathway is a summation of several randomly selected compact extreme pathways (EPs). (2) The probability of an EP being selected is ***p***. (3) Altogether, ***t*** artificial pathways are generated and the unique ones form the set. One hundred different artificial pathway sets are built for certain values of ***p*** and ***t***. The green dashed and dotted line indicates the expectation of the evaluation score and the solid red line indicates the evaluation score of the EqSet sequences calculated by our approach, namely **Algorithm A.**

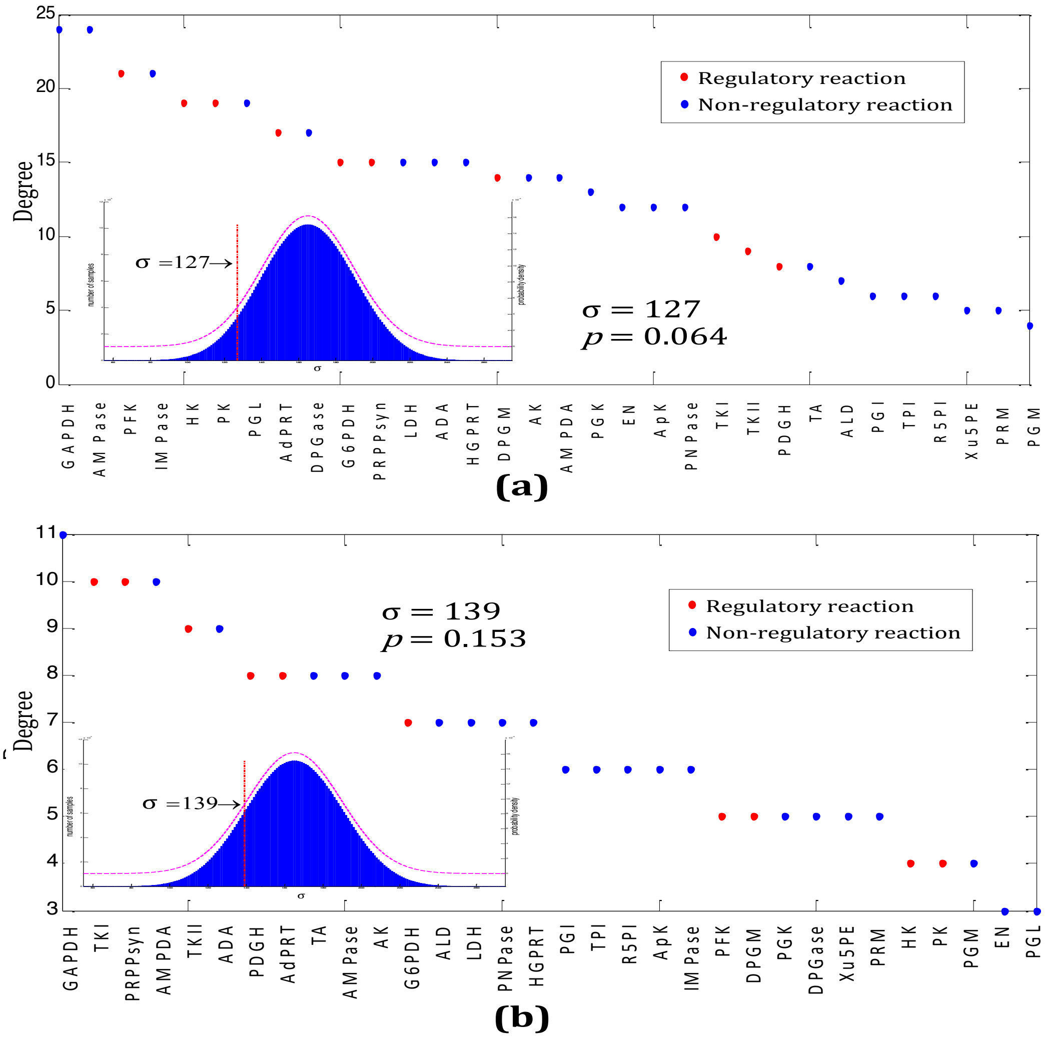

Fig K. Degrees of connection of the internal reactions in the hRBC metabolic network. All the internal reactions are sorted in the descending order of their degrees of connection. The regulatory reactions are placed in front of the reactions whose degrees of connection are equal. A regulatory reaction is denoted by a red dot and a non-regulatory one is denoted by a blue dot. The subplot in each graph shows the distributions of the evaluation scores for random sequences of 32 reactions, in which 10 are regulatory reactions, and the dotted vertical bars indicate the position of the resulting sequence in the distribution. (a) The degrees of connection were counted when all the metabolites were kept in the network. The evaluation score of the resulting sequence of the internal reactions is 127 and the corresponding *p*-value is 0.064 when the reactions were sorted in the descending order of the degrees of connection. If the reactions were sorted in the ascending order of connectedness, then the resulting sequence would have 190 as its evaluation score and 0.847 as the *p*-value. (b) The degrees of connection were counted after removing the currency metabolites, H, H_2O, ATP, PI and ADP, from the network. The evaluation score of the resulting sequence of the internal reactions is 139 and the corresponding *p*-value is 0.153 when the reactions were sorted in the descending order of the degrees of connection. If the reactions were sorted in the ascending order of connectedness, then the resulting sequence would have 168 as its evaluation score and 0.556 as the *p*-value. The regulatory reactions show no significant tendency of ranking higher or lower in these sequences.

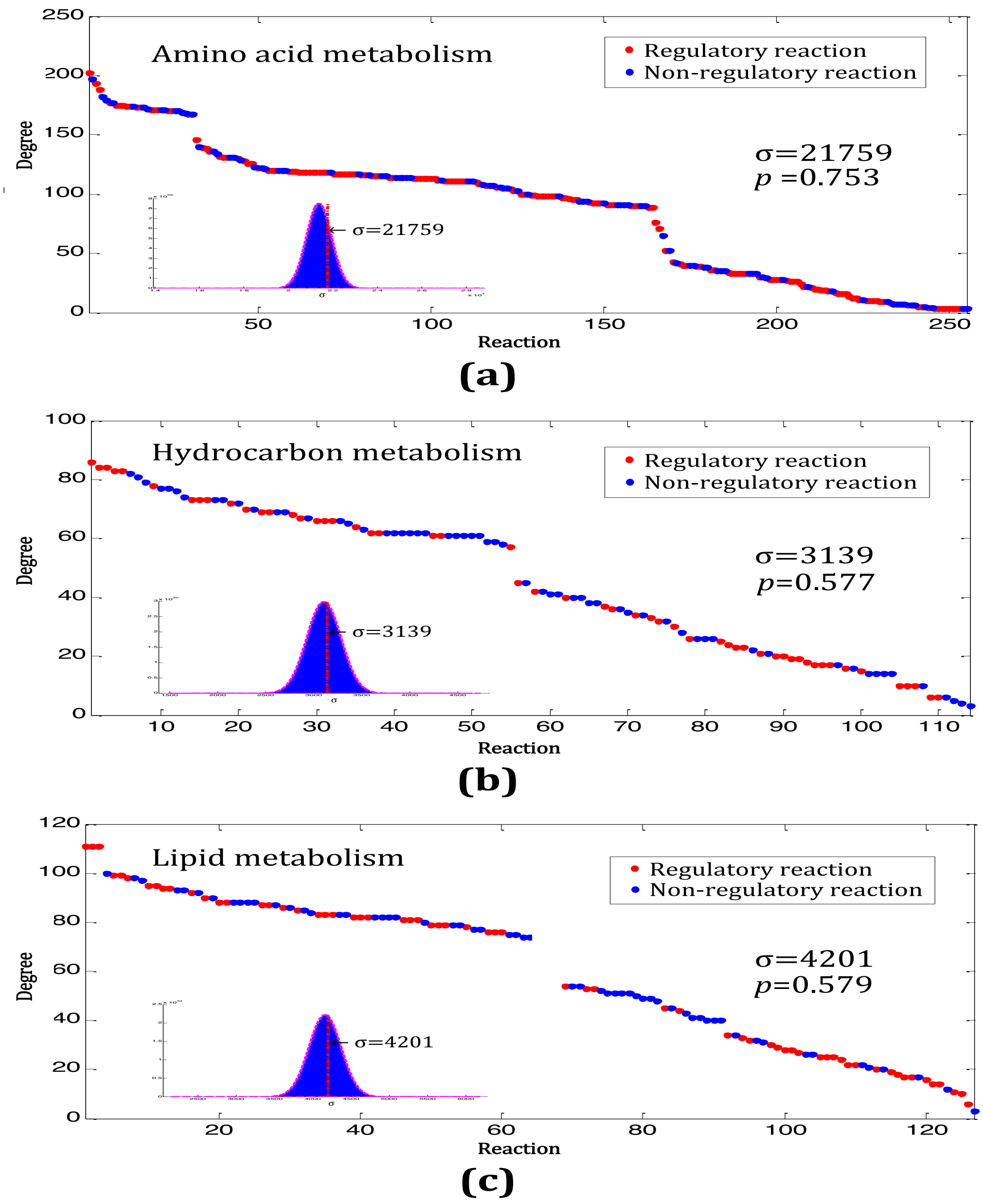

Fig L. Degrees of connection of the internal reactions in the three target subsystems of the *E.coli* metabolic network. All the internal reactions are sorted in the descending order of their degrees of connection. The regulatory reactions are placed in front of those whose degrees of connection are equal. A regulatory reaction is denoted by a red dot and a non-regulatory one is denoted by a blue dot. The subplot in each graph shows the distributions of the evaluation scores for random sequences of the internal reactions in each subsystem, and the dotted vertical bars indicate the position of the resulting sequence in the distribution. The reactions were sorted in the descending order of the degrees of connection. (a) The reaction sequence of the amino acid metabolic subsystem. (b) The reaction sequence of the hydrocarbon metabolic subsystem. (c) The reaction sequence of the lipid metabolic subsystem. If the reactions were sorted in the ascending order of connectedness, then the resulting sequences would have 0.165, 0.302 and 0.334 as the *p*-values for the subsystems, amino acid metabolism, hydrocarbon metabolism and lipid metabolism, respectively. The results suggest that the regulatory reactions show no significant tendency of ranking higher or lower in these sequences.

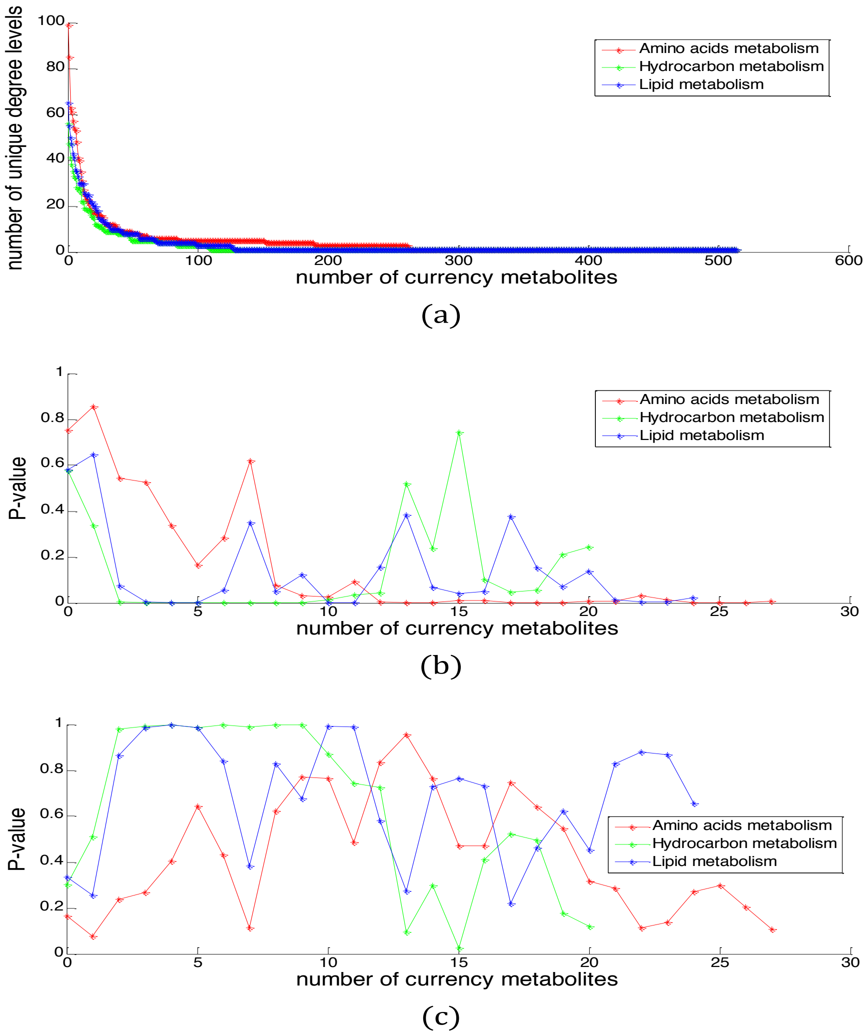

Fig M. *P*-values of the sequences as functions of the number of currency metabolites neglected in the counting of the degrees of connection. (a) Plot of the number of different values for the degree of connection (the vertical axis) versus the number of currency metabolites (the horizontal axis). As the number of currency metabolites increases, the number of unique levels of connectedness falls sharply, which means the reactions will quickly lose diversity in the degree of connection. Therefore, the number of currency metabolites was constrained from 0 to 28, 21, and 25 in (b) and (c) for the subsystems of amino acids metabolism, hydrocarbon metabolism and lipid metabolism, respectively, to ensure that the number of unique levels of connectedness of each subsystem is higher than 15. (b) P-value (the vertical axis) of the reaction sequences in the descending order of connectedness as a function of the number of currency metabolites (the horizontal axis). (c) P-value (the vertical axis) of the reaction sequences in the ascending order of connectedness as a function of the number of currency metabolites (the horizontal axis). There is no evident tendency shown in the variation of p-value in (b) and (c). By comparing (b) and (c), we may find that the reactions with higher degrees of connection are more likely to be regulatory ones. Neglecting some currency metabolites apparently decreases the p-values of the reaction sequences; however, two big reasons for the low p-value are that many reactions have the same value of connectedness and among them the known regulatory reactions were placed in front of the others. In summary, it is difficult to recognize the regulatory reactions by only their degrees of connection.

Supplementary References

1. Schilling, C.H., Letscher, D. & Palsson, B.O. Theory for the systemic definition of metabolic pathways and their use in interpreting metabolic function from a pathway-oriented perspective. *Journal of Theoretical Biology* **203**, 229-248 (2000).
2. Mahadevan, R. & Schilling, C.H. The effects of alternate optimal solutions in constraint-based genome-scale metabolic models. *Metabolic Engineering* **5**, 264-276 (2003).
3. Schilling, C.H. & Palsson, B.O. Assessment of the metabolic capabilities of Haemophilus influenzae Rd through a genome-scale pathway analysis. *Journal of Theoretical Biology* **203**, 249-283 (2000).
4. Covert, M.W., Knight, E.M., Reed, J.L., Herrgard, M.J. & Palsson, B.O. Integrating high-throughput and computational data elucidates bacterial networks. *Nature* **429**, 92 - 96 (2004).
5. Reed, J.L., Vo, T.D., Schilling, C.H. & Palsson, B.O. An expanded genome-scale model of Escherichia coli K-12 (iJR904 GSM/GPR). *Genome Biology* **4**, R54 (2003).
6. Barabasi, A.L. & Oltvai, Z.N. Network biology: understanding the cell's functional organization. *Nat Rev Genet* **5**, 101-113 (2004).
7. Wiback, S.J. & Palsson, B.O. Extreme pathway analysis of human red blood cell metabolism. *Biophysical Journal* **83**, 808-818 (2002).
8. Nelson, D.L. & Cox, M.M. Lehninger Principles of Biochemistry (fourth edition), (W.H. Freeman, 2005).
9. Wilson, J.E. Distinguishing the type I and type II isozymes of hexokinase: the need for a reexamination of past practice. *Diabetes* **47**, 1544-1548 (1998).
10. Lubert, S., Mark, B.J. & L, T.J. Biochemistry (Sixth edition). (W.H. Freeman, San Francisco; 2007).
11. Dunaway, G.A., Kasten, T.P., Sebo, T. & Trapp, R. Analysis of the phosphofructokinase subunits and isoenzymes in human tissues. *Biochem J* **251**, 677-683 (1988).
12. Luzzatto, L. Regulation of the activity of glucose-6-phosphate dehydrogenase by NADP+ and NADPH. *Biochim Biophys Acta* **146**, 18-25 (1967).
13. Yoshida, A. & Lin, M. Regulation of glucose-6-phosphate dehydrogenase activity in red blood cells from hemolytic and nonhemolytic variant subjects. *Blood* **41**, 877-891 (1973).
14. Rippa, M., Giovannini, P.P., Barrett, M.P., Dallocchio, F. & Hanau, S. 6-Phosphogluconate dehydrogenase: the mechanism of action investigated by a comparison of the enzyme from different species. *Biochim Biophys Acta* **1429**, 83-92 (1998).
15. Takeuchi, T., Nishino, K. & Itokawa, Y. Purification and characterization of, and preparation of an antibody to, transketolase from human red blood cells. *Biochim Biophys Acta* **872**, 24-32 (1986).
16. Lonsdale, D. Three case reports to illustrate clinical applications in the use of erythrocyte transketolase. *Evidence Based Complementary and Alternative Medicine* **4**, 247-250 (2007).
17. Crespillo, J., Llorente, P., Argomaniz, L. & Montero, C. APRT from erythrocytes of HGPRT deficient patients: kinetic, regulatory and thermostability properties. *Mol Cell Biochem* **254**, 359-363 (2003).
18. Arnold, W.J. & Kelley, W.N. Adenine phosphoribosyltransferase. *Methods Enzymol* **51**, 568-574 (1978).
